# Public health impact and cost effectiveness of routine and catch-up vaccination of girls and women with a nine-valent HPV vaccine in Japan: a model-based study

**DOI:** 10.1186/s12879-020-05632-0

**Published:** 2021-01-06

**Authors:** Palmer Cody, Keisuke Tobe, Machiko Abe, Elamin H. Elbasha

**Affiliations:** 1 Merck &Co., Inc., Kenilworth, NJ USA; 2 Center for Observational and Real-world Evidence (CORE), Merck & Co., Inc., WP37A-150, PO Box 1000, West Point, PA 19486 USA; 3 MSD.K.K., Tokyo, Japan

**Keywords:** Human papillomavirus, Intraepithelial neoplasia, Cervical, Vulvar, Vaginal, Anal, Oral cavity, And oropharyngeal cancer, Condylomata acuminate, Genital warts, Recurrent respiratory papillomatoses, Japan, Vaccines, Disease transmission models, Herd protection/immunity, Cost effectiveness analysis, Epidemiology

## Abstract

**Background:**

Combined with cancer screening programs, vaccination against human papillomavirus (HPV) can significantly reduce the high health and economic burden of HPV-related disease in Japan. The objective of this study was to assess the health impact and cost effectiveness of routine and catch-up vaccination of girls and women aged 11–26 years with a 4-valent (4vHPV) or 9-valent HPV (9vHPV) vaccine in Japan compared with no vaccination.

**Methods:**

We used a mathematical model adapted to the population and healthcare settings in Japan. We compared no vaccination and routine vaccination of 12–16-year old girls with 1) 4vHPV vaccine, 2) 9vHPV vaccine, and 3) 9vHPV vaccine in addition to a temporary catch-up vaccination of 17–26 years old girls and women with 9vHPV. We estimated the expected number of disease cases and deaths, discounted (at 2% per year) future costs (in 2020 ¥) and discounted quality-adjusted life years (QALY), and incremental cost effectiveness ratios (ICER) of each strategy over a time horizon of 100 years. To test the robustness of the conclusions, we conducted scenario and sensitivity analyses.

**Results:**

Over 100 years, compared with no vaccination, 9vHPV vaccination was projected to reduce the incidence of 9vHPV-related cervical cancer by 86% (from 15.24 new cases per 100,000 women in 2021 to 2.02 in 2121). A greater number of cervical cancer cases (484,248) and cancer-related deaths (50,102) were avoided through the described catch-up vaccination program. Routine HPV vaccination with 4vHPV or 9vHPV vaccine prevented 5,521,000 cases of anogenital warts among women and men. Around 23,520 and 21,400 diagnosed non-cervical cancers are prevented by catch-up vaccination among women and men, respectively. Compared with no vaccination, the ICER of 4vHPV vaccination was ¥975,364/QALY. Compared to 4vHPV, 9vHPV + Catch-up had an ICER of ¥1,534,493/QALY.

**Conclusions:**

A vaccination program with a 9-valent vaccine targeting 12 to 16 year-old girls together with a temporary catchup program will avert significant numbers of cases of HPV-related diseases among both men and women. Furthermore, such a program was the most cost effective among the vaccination strategies we considered, with an ICER well below a threshold of ¥5000,000/QALY.

## Background

A substantial burden of disease results from persistent human papillomavirus (HPV) infections. For example, there were approximately 11,000 new cases of HPV-caused cervical cancer (CC) and 2800 deaths in Japan in 2017 [1]. Evidence from population-based cancer registries from three Japanese prefectures (Yamagata, Fukui, and Nagasaki) suggests that age-standardized CC incidence has been increasing since 1997 at a rate of 2.6% per year [2]. HPV also causes genital warts and recurrent respiratory papillomatosis (RRP) and cancers of the vagina, vulva, penis, anus, and oropharynx [3]. For example, 61 per 100,000 new cases of genital warts among men and women were diagnosed in 2015 in Japan [4]. Age-standardized incidence of other HPV-related cancers in 2018 in Japan ranged from 0.1 to 1.5 per 100,000 [5] . The economic burden associated with HPV disease in Japan is high and increasing over time. For example, the cost of illness from cervical cancer increased by 66%, from ¥96.1 billion in 1996 to ¥159.9 billion in 2011 [6].

There are two types of interventions to prevent HPV-related diseases: secondary prevention through cancer screening (i.e., population-based or opportunistic cervical or vaginal cancer screening) or primary prevention through HPV vaccination. Cervical screening of women aged over 30 years once a year has been practiced in Japan since 1982. The program was modified in 2004 to allow women of age 20 years old and over to receive cervical screening test every other year [7]. However, uptake rate of cervical cancer screening is relatively low, estimated at 35 to 40% [7, 8].

A bivalent human papillomavirus (HPV) vaccine (HPV types 16 and 18) was licensed in Japan in 2009. In 2011, a quadrivalent (4vHPV) vaccine (HPV 6, 11, 16, and 18) was approved and indicated for use in girls and women for the prevention of cervical cancer and precursor lesions, vulvar and vaginal intraepithelial neoplasia, and genital warts. However, a 9-valent (9vHPV) HPV vaccine (HPV 6, 11, 16, 18, 31, 33, 45, 52, and 58), introduced in many countries since 2014, was recently approved in Japan [9]. Also, none of these HPV vaccines are currently available for use in males in Japan.

Routine vaccination of girls aged 12–16 years against HPV was included in the national immunization program (NIP) in April of 2013. However, following unconfirmed reports of adverse events in the media, the Japanese Ministry of Health, Labour, and Welfare (MHLW) suspended proactive recommendations for routine use of the HPV vaccine in NIP in June 2013 [10, 11]. Notwithstanding the conclusion of the Vaccine Adverse Reactions Review Committee of no causal association between the HPV vaccine and the reported adverse events after vaccination in January 2014, MHLW has not yet reinstated proactive recommendations for the routine HPV vaccination. The combined effect of news media reports and the suspension of the MHLW’s recommendation on HPV vaccination rate is dramatic: a sharp decline from over 70% in 2013 to a current rate of less than 1% among girls and young women [12].

The Japanese government recently approved the use of the 9vHPV vaccine [9]. There are also calls to vaccinate older women and introduce the 9-valent HPV vaccine in Japan [13]. Previous studies have established the cost effectiveness of 4vHPV vaccine in in Japan [14, 15]. Information on the public health impact and cost effectiveness of various HPV vaccination strategies will be paramount in informing future policy decision makers in Japan.

The cost effectiveness of the 9vHPV vaccine has been evaluated in many countries, particularly those with established 4vHPV vaccine programs [16]. In the United States, cost effectiveness of the 9vHPV vaccine compared with the 2vHPV and 4vHPV vaccines has been demonstrated using established vaccine prices [17, 18] In the United Kingdom the cost effectiveness of 9vHPV was analyzed, though it was not explicitly compared to the 4vHPV (4vHPV girls only vaccination was compared to 4vHPV gender neutral vaccination, and 9vHPV girls only vaccination was compared to 9vHPV gender neutral vaccination) . Incremental price thresholds for the 9vHPV vaccine compared to 4vHPV have been investigated in several other countries [19–21].. In a majority of GAVI-eligible countries with negotiated vaccine prices, it was found that the 9vHPV vaccine would be cost effective compared to 2vHPV vaccination, even under favorable cross-protection assumptions for 2vHPV [22]. Information on the public health impact and cost-effectiveness of various HPV vaccination strategies of will be useful to more properly inform future policy decision makers in Japan. To our knowledge, no previous study has considered the cost effectiveness of the 9vHPV vaccine in Japan. In the United Kingdom the cost effectiveness of 9vHPV was analyzed, though it was not explicitly compared to the 4vHPV (4vHPV girls only vaccination was compared to 4vHPV gender neutral vaccination, and 9vHPV girls only vaccination was compared to 9vHPV gender neutral vaccination) [23]. In a majority of GAVI-eligible countries with negotiated vaccine prices, it was found that the 9vHPV vaccine would be cost effective compared to 2vHPV vaccination, even under favorable cross-protection assumptions for 2vHPV [22]. To our knowledge, no previous study has considered the cost effectiveness of the 9vHPV vaccine in Japan.

In this study, we evaluated population-level health impact and cost effectiveness of routine and catch-up vaccination of girls and women aged 11–26 years with a 4-valent or 9-valent HPV vaccine in Japan compared with no vaccination.

## Methods

We updated and adapted a previously published mathematical platform of HPV disease transmission to the Japanese setting [24, 25]. The platform consists of a collection of individual continuous-time compartmental, deterministic population-based disease transmission models structured by age, sex, and sexual activity. It was designed to capture the direct and indirect ‘herd protection/immunity’ effects of vaccination and assess cost-effectiveness analysis of HPV vaccination programs. All data used to parameterize this model came from publicly available sources which are provided here and in the supplementary information.

### Demographic, behavioral, and epidemiological models

The demographic model divides the two-sex, heterosexually mixing population into 23 age groups (0 to < 1, 1–8, 9–11, 12, 13–14, 15–16, 17–18, 19, 20–24, 25–26, 27–29, 30–34, 35–39, 40–44, 45–49, 50–54, 55–59, 60–64, 65–69, 70–74, 75–79, 80–84, and 85 or older). Same-sex partnerships are not modeled here. The behavioral model defines risk of HPV transmission according to sex, age, sexual activity (low, medium, and high), and rates of new sex partners per year, all based on sexual activity data from Japan [26, 27] which are used to construct a sexual mixing matrix as in [24, 25].

The epidemiological models simulate the transmission of the nine vaccine-related HPV types (6, 11, 16, 18, 31, 33, 45, 52, 58) and development of disease using the following 7 groups: cervical, vaginal, vulvar pre-cancers and cancers in women; penile pre-cancer and cancer in men; anal pre-cancer and cancer and head/neck cancer in both men and women; and low-risk cervical intraepithelial neoplasia (CIN) in women, and genital warts (GW) and recurrent respiratory papillomatosis (RRP) in men and women. For simplicity, we developed separate and independent models for each HPV-type/disease group combination. For example, there is a separate model HPV 16 CIN and cervical cancer and a model for HPV 18 anal intraepithelial neoplasia (AIN) and anal cancer. By using this structure, we are assuming that the spread of the various virus types are independent, and that infection by one strain neither increases or decreases the chances of being infected with another strain. In total, the current analysis utilized 44 (7 HPV types × 6 cancers + 2 HPV types X 1 GW/CIN1/RRP) models.

Solutions of these epidemiological models for each vaccination strategy consist of numerical functions that describes the number of individuals in each compartment over time. The solved functions are then aggregated into familiar epidemiological variables such as new cases and number of individuals living with cancer over time.

### Economic model

The continuous-time economic model uses output from the epidemiological models such as new cases to calculate various types of costs for each vaccination strategy. For example, new cases of cervical cancer at each time are multiplied by lifetime discounted cost per case to obtain healthcare costs of cervical cancer. Similarly, multiplication of number of doses by cost per dose gives total vaccination cost for a given strategy. Numerical functions representing the number of individuals in each compartment, whether living with disease or not, are multiplied by corresponding health utilities and integrated over time to obtain quality-adjusted life years (QALYs). Cost effectiveness of a vaccination strategy relative to a comparator is evaluated using the incremental cost-effectiveness ratio (ICER) obtained by dividing incremental total discounted costs by the incremental total discounted number of QALYs.

### Screening and vaccination strategies

We considered and assumed no future changes in disease management practices and evaluated the following primary prevention (through HPV vaccination) and secondary prevention (through cervical cancer screening) strategies:

1. Cervical cancer screening only and no vaccination (Screen)
2. Cervical cancer screening and routine vaccination of 12–16-year old girls with 4vHPV vaccine (Routine GOV-4vHPV) from 2021 onwards
3. Cervical cancer screening and routine vaccination of 12–16-year old girls with 9vHPV vaccine (Routine GOV-9vHPV) from 2021 onwards
4. Cervical cancer screening and routine vaccination of 12–16-year old girls from 2021 onwards and temporary catch-up vaccination of 17–26 years old girls and women with 9vHPV until 2025 (Routine + catch-up GOV-9vHPV)

Due to the recent approval of 9vHPV and given the additional benefit of the 9vHPV vaccine, we do not expect that the routine vaccination plus catchup with 4vHPV to be seriously considered as a viable strategy. We have included routine vaccination with 4vHPV, since it represents the state of the program when the government rescinded its active recommendation.

### Model parameters: values and sources

All model input parameters were either calibrated or derived from published sources as described in the supplemental material. Table 1 includes selected demographic parameters, cost, quality of life weightings, and vaccine input values used in the base-case analysis.

**Table 1:** Selected input values used in the base-case analysis

Parameter	Value		Reference
Demographic parameters	**Males**	**Females**	[28]
Population size	61,765,503	65,167,269	
Birth rate (births/year)	780,581	723,233	
Mean number of sexual partners per year by sex and age			[26, 27]
13–14	1.28	1.18	
15–17	1.08	1.09	
18–24	1.10	1.08	
25–34	1.70	1.13	
35–44	1.72	1.05	
≥ 45	1.17	1.04	
Percent of the population in each sexual activity categories			[29]
Low (mean number of sexual partners per year: 0–1)	76.7	89.1	
Medium (mean number of sexual partners per year: 2–4)	19.0	9.5	
High (mean number of sexual partners per year: 5+)	4.2	1.3	
Cost of screening, diagnosis, and treatment (2020 ¥, single outpatient/inpatient cost)			[30]
Cervical screening and visit	3730		
Coloscopy	5100		
Biopsy	7000		
Episode of CIN 1	76,000		
Episode of CIN 2 or worse	276,400		
Episode of VaIN 1	76,000.0		
Episode of VaIN 2 or worse	533,700.0		
Case of cervical cancer			
Localized	2,181,900		
Regional	2,914,300		
Distant	4,020,800		
Case of vaginal cancer			
Localized	991,400		
Regional	1,823,400		
Distant	1,977,400		
Case of vulvar cancer			
Localized	818,500		
Regional	1,694,800		
Distant	1,847,200		
Case of penile cancer			
Localized	647,000		
Regional	1,165,000		
Distant	1,249,600		
Case of anal cancer	**Males**	**Females**	
Localized	1,183,000	1,183,000	
Regional	1,809,200	1,809,200	
Distant	2,659,300	2,659,300	
Case of oropharyngeal cancer			
Localized	1,169,110	1,169,110	
Regional	3,695,570	3,695,570	
Distant	5,794,276	5,794,276	
Case of condyloma acuminatum (anogenital warts)	11,246	11,356	
Case of recurrent respiratory papillomatosis	1,144,296	1,144,296	
Cost of vaccination (2020 ¥)			
3-dose series per dose, 4vHPV	12,000		
3-dose series per dose, 9vHPV	20,000		
Administration fee per dose	3718		
Discount rate per year (%): future costs and effectiveness	2		

*4vHPV* 4-valent human papillomavirus (HPV) vaccine, *9vHPV* 9-valent human papillomavirus (HPV) vaccine, *CIN 1* Cervical intraepithelial neoplasia grade 1, *CIN 2* Cervical intraepithelial neoplasia grade 2, *¥* Japanese Yen, *VaIN 1* Vaginal intraepithelial neoplasia grade 1, *VaIN 2* Vaginal intraepithelial neoplasia grade 2

#### Demographic parameters

Sex- and age-specific population size and annual all-cause mortality rates in 2017 were extracted from Japanese Government Statistics [31] (Supplemental material Tables 1–2).

#### Sexual behavior parameters

Sexual behavior is stratified by age and sexual activity class. The population is separated into low, medium, and high sexual activity classes. Data on the number of partners by age and sexual activity class, the proportion of the population in each sexual activity class, and the degree of sexual mixing across age and class were extracted from two surveys [26, 27] (Supplemental material Tables 3–6).

#### Clinical parameters

Parameters related to the natural history of HPV-related diseases for the various sites were either taken from the literature, calibrated, or taken from previous model calibrations (Supplemental material Section 2.5).

#### Vaccine uptake, series completion, and efficacy parameters

The base case assumed that up to 70% of all 12-year-old Japanese girls would complete the 3-dose series [3]. Uptake was assumed to occur gradually beginning at 42% in 2021 and reaching 70% in 2025 and continuing thereafter. Girls aged 13–16 years were also assumed to experience similar temporary gradual vaccine uptake during the period 2021–2025. After 2025, all the 70% coverage among the primary cohort of girls aged 12–16 years was assumed to result from vaccinating 12-year old girls. A gradual annual uptake was assumed for the temporary catch-up program for 17–26-year-old girls and women reaching a target coverage of 50% by 2025 (Supplemental material Fig. 2).

#### Health utilities

Values of health utilities associated with the various health states in the model were derived from published studies (Supplementary Material) and adjusted to the Japanese population norm using the mean EQ-5D-3L quality of life score [32].

#### Costs

Costs were based on 2020 prices and are evaluated from a public healthcare payer’s perspective. The base-case vaccine cost acquisition cost per dose of 4vHPV was ¥12,000. The vaccine cost acquisition cost per dose of 9vHPV was assumed to be ¥20,000 (range: ¥18,000–¥22,000, Table 1). The technical administration fee per dose was set at ¥3718.

#### Time horizon and discounting parameters

Because it may take a long time before the impact of HPV vaccination on important clinical outcomes such as HPV-related cancers to materialize, the analysis time horizon was chosen to be 100 years. Based on Guideline for Preparing Cost-Effectiveness Evaluation to the Central Social Insurance Medical Council, a 2% annual discount rate was applied to all future costs and QALYs [33].

#### Model calibration and validation

Model calibration is described in detail within the technical appendix. Bayesian history matching [34] was used to identify non-implausible portions of the parameter space. These non-implausible regions were sampled using a posterior likelihood to produce a Maximum Likelihood Estimator (MLE), and confidence intervals for the calibrated parameters.

### Base-case, scenario, and sensitivity analysis

We used the base-case values of parameters to estimate the per capita total costs and QALYs associated with each vaccination strategy, and ICER per additional QALY of strategies compared incrementally relative to no vaccination. In addition, we projected the incidence of cervical and other HPV-related cancers and deaths, pre-cancers, genital warts, and cases of disease avoided as result of vaccination. Total cases and deaths averted are computed assuming a total population of 126,932,722 people in Japan.

Finally, we performed scenario and one-way sensitivity analyses to test sensitivity of outcomes to uncertainty in epidemiology, vaccination coverage, health utilities, discount rates, and cost inputs [33]. We varied the parameters below as follows:

1. Target vaccine coverage in the primary cohort (60, 80, and 100%).
2. Adherence to prescribed number of doses (80 and 100%).
3. Duration of protection of the vaccine (Lifetime vs 10 years for 1-dose, 20 years for 2 doses, and 30 years for 3 doses).
4. Healthy utility weights (±20%, up to a maximum of 1).
5. Disease utility weights (±20%, up to a maximum of 1).
6. Disease costs (±20%).
7. Varying the price of 9vHPV only (¥18,000 and 22,000).
8. Discount Rate (0, 3, and 4%)

## Results

### Public heath impact of vaccination strategies

Over 100 years, the incidence of cervical cancer is expected to drop dramatically, particularly in scenarios where 9vHPV vaccine is used. The estimated public health impact of the various vaccination strategies on other HPV-related diseases is also profound.

#### Reduction in cervical cancer

**Fig. 1:**
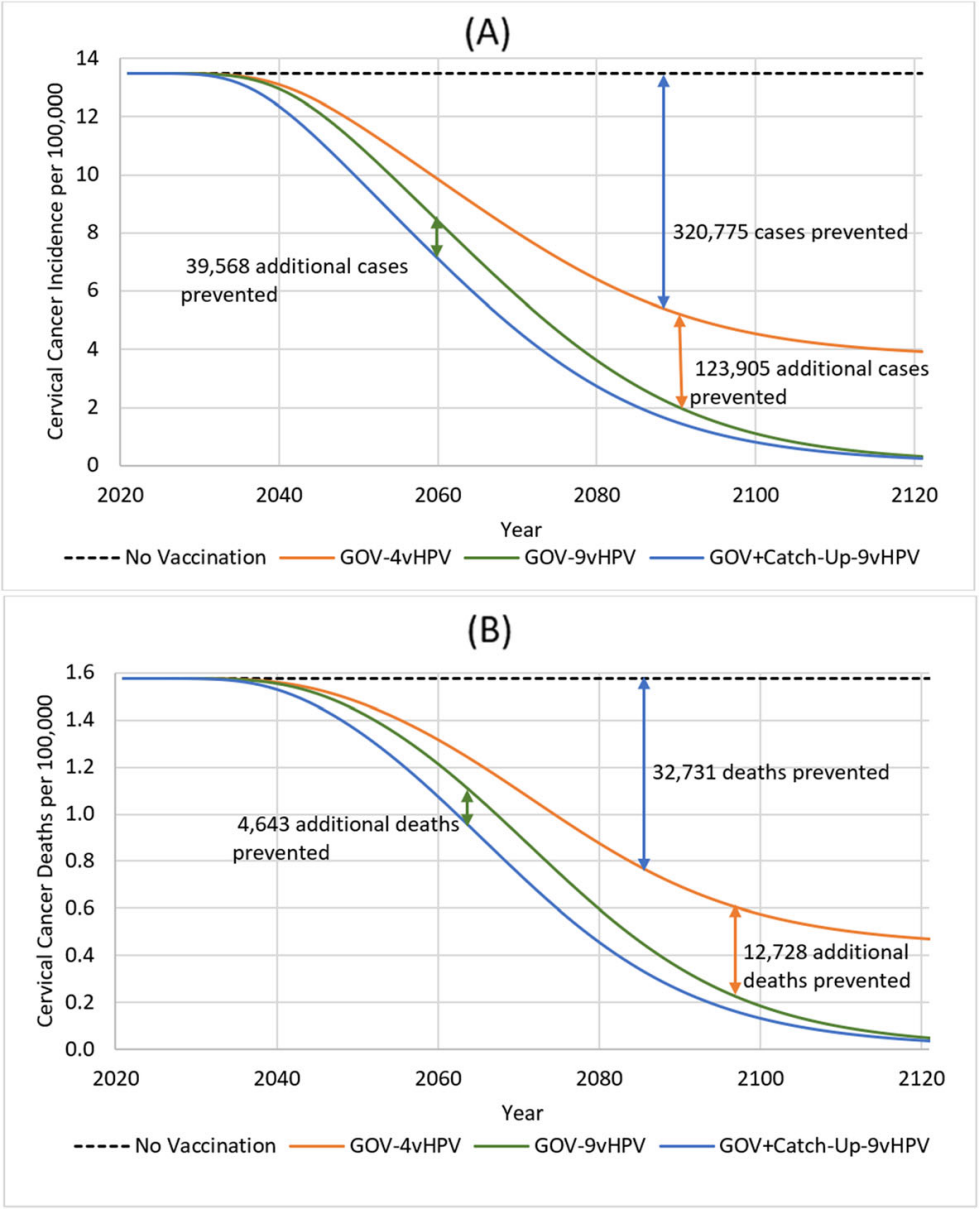
Population-level health impact of routine and alternative strategies of vaccinating girls and women aged 12 through 26 years on 9-valent HPV-related (**a**) cervical cancer and (**b**) cervical cancer death. GOV-4vHPV included routine vaccination of 12–16-year old girls with 4vHPV from 2021 onwards; GOV-9vHPV included routine vaccination of 12–16-year old girls with 9vHPV; GOV + Catch-Up-9vHPV consisted of routine vaccination of 12–16-year old girls from 2021 onwards and temporary catch-up vaccination of 17–26 years old girls and women with 9vHPV until 2025. All vaccination strategies were combined with cervical cancer screening

Given the relatively high incidence of cervical cancer in Japan, HPV vaccination can prevent a large number of cervical cancer cases. Over the 100-year time horizon, there was large drops in the incidence of cancer attributable to the 9 types covered by 9vHPV vaccine, and the overall incidence of cervical cancer (Fig. 1). Compared with screening only and no vaccination, GOV-4vHPV reduced 9-valent-related cervical cancer by 77%, thereby averting 320,775 cervical cancer cases. With 9vHPV vaccination, the overall cancer incidence decreased by over 86% (from 15.24 per 100,000 women in 2021 to 2.02 per 100,000 women in 2121). Earlier and greater reductions in incidence were achieved through catch-up vaccination programs. As a result, the cumulative incremental impact of the GOV-9vHPV catch-up program relative to GOV-9vHPV alone was significant (preventing 39,568 additional cases of cervical cancer over 100 years). Consequently, up to 484,248 cases of cervical cancer were avoided if a 9vHPV vaccination catch-up is used compared with no vaccination (Fig. 1).

The reduction in the number of cases of cervical cancer was associated with a significant reduction in the number of cervical cancer deaths (Fig. 1). Compared with no vaccination, up to 50,102 deaths are averted if a GOV-9vHPV catch-up program is implemented.

#### Reduction in other HPV-related disease

**Fig. 2:**
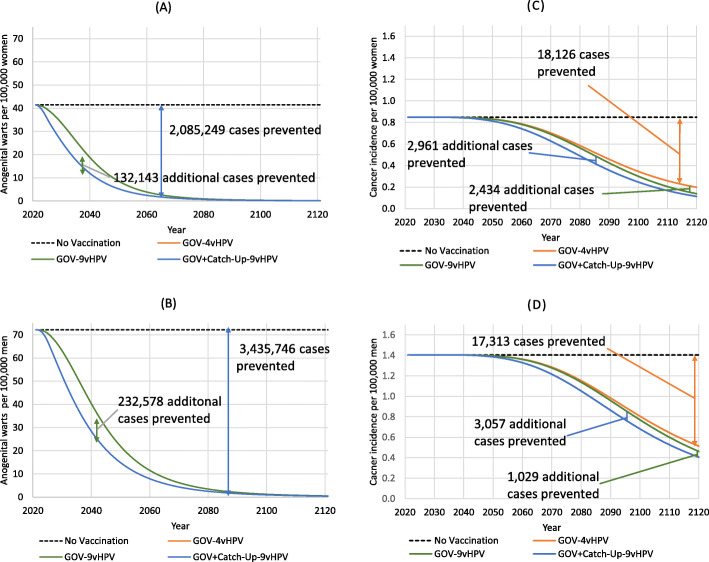
Population-level health impact of routine and alternative strategies of vaccinating girls and women aged 12 through 26 years on 9-valent HPV-related anogenital warts and other non-cervical cancers. **a** Diagnosed anogenital warts among women (arrows spanning the curves represent the incremental benefit between strategies). **b** Diagnosed anogenital warts among men. **c** Diagnosed non-cervical cancer among women **d** Diagnosed cancer among men.GOV-4vHPV included routine vaccination of 12–16-year old girls with 4vHPV from 2021 onwards; GOV-9vHPV included routine vaccination of 12–16-year old girls with 9vHPV; GOV + Catch-Up-9vHPV consisted of routine vaccination of 12–16-year old girls from 2021 onwards and temporary catch-up vaccination of 17–26 years old girls and women with 9vHPV until 2025. All vaccination strategies were combined with cervical cancer screening

In addition to cervical disease, we have also quantified the impact of vaccination on other HPV-related disease. The impact of vaccination on the incidence of genital warts was significant (Fig. 2 A,B). Compared with no vaccination, routine HPV vaccination with 4vHPV or 9vHPV vaccine lasting 100 years prevented 2,085,249 and 3,435,746 cases of anogenital warts among women and men, respectively. An additional 364,721 number of genital warts cases among both women and men was prevented by the catch-up program. While many of other HPV-caused cancers have lower incidence compared with cervical cancer, they cause significant burden of disease. Consequently, approximately 23,520 and 21,400 diagnosed cancers are prevented by catch-up vaccination among women and men, respectively (Fig. 2 C, D). Detailed breakdown of cases and deaths averted by disease and vaccination strategy, over the 100-year time horizon are given in Table 2. By considering these other diseases, an additional 11,653 deaths will be averted on top of the averted deaths from cervical cancer.

**Table 2:** Cumulative cases and deaths of 9-valent HPV-related diseases in the Japanese population prevented, by strategy

Strategy	Anal Cancer		Oropharyngeal Cancer		Penile Cancer	Vaginal Cancer	Vulvar Cancer	JORRP	
	Male	Female	Male	Female	Male	Female	Female	Male	Female
Cases									
4vHPV	5664	7775	11,414	5392	235	3152	1807	4587	3193
9vHPV	6147	8355	11,784	5618	411	4264	2323	4587	3193
9vHPV + Catch Up	7105	9541	13,824	6455	470	4856	2669	4875	3393
Deaths									
4vHPV	778	960	4114	1760	42	897	290	206	143
9vHPV	845	1032	4247	1834	70	1220	377	206	143
9vHPV + Catch Up	983	1186	5041	2137	81	1412	441	220	153

Girls-only vaccination with 4vHPV included routine vaccination of 12–16-year old girls with 4vHPV from 2021 onwards; Girls-only vaccination with 9vHPV included routine vaccination of 12–16-year old girls with 9vHPV; Girls-only routine and catch-up vaccination with 9vHPV consisted of routine vaccination of 12–16-year old girls from 2021 onwards and temporary catch-up vaccination of 17–26 years old girls and women with 9vHPV until 2025. All vaccination strategies were combined with cervical cancer screening

### Cost effectiveness of vaccination strategies

The cost effectiveness of each strategy was considered using a standard ICER-based decision rule to determine the cost-effectiveness frontier. Ordered from least effective (no vaccination) at the top to most effective (GOV-9vHPV + catch-up vaccination) at the bottom, costs, QALYs, and the ICER for each vaccination strategy are shown in Table 3 . Compared with no vaccination, GOV-4vHPV was more costly and more effective. The ICER of 4vHPV vaccination compared with no vaccination was ¥975,364/QALY. When compared to 4vHPV vaccination, 9vHPV + Catch up vaccination had an ICER of ¥1,534,493/QALY. The strategy with 9vHPV vaccination without catchup was weakly dominated, meaning that the ICER compared to 4vHPV vaccination (¥1,966,136/QALY) was higher that the ICER for 4vHPV vaccination versus 9vHPV with catchup, and is thus not on the cost-effectiveness frontier.

**Table 3:** Total discounted costs and QALYs per capita, and the ICER associated with each vaccination strategy

Vaccination Strategy	Discounted screening and treatment costs (¥)	Discounted vaccination costs (¥)	Total Discounted Costs (¥)^*^	Total Discounted QALYs (years)	ICER (¥/QALYs)
No vaccination, screening only	36,455	–	36,455	39.71150	–
Girls-only routine vaccination with 4vHPV	33,116	8711	41,827	39.71701	975,364
Girls-only routine vaccination with 9vHPV	31,981	13,145	45,126	39.71868	Weakly dominated^a^
Girls-only routine and catch-up vaccination with 9vHPV	31,335	14,824	46,160	39.71983	1,534,493

*ICER* Incremental cost-effectiveness ratio, *HPV* Human papillomavirus, *¥* Japanese Yen, *QALY* Quality-adjusted life years. Girls-only vaccination with 4vHPV included routine vaccination of 12–16-year old girls with 4vHPV from 2021 onwards; Girls-only vaccination with 9vHPV included routine vaccination of 12–16-year old girls with 9vHPV; Girls-only routine and catch-up vaccination with 9vHPV consisted of routine vaccination of 12–16-year old girls from 2021 onwards and temporary catch-up vaccination of 17–26 years old girls and women with 9vHPV until 2025. All vaccination strategies were combined with cervical cancer screening

^a^Girls-only routine vaccination with 9vHPV is weakly dominated (and shouldn’t be implemented) because it has an ICER of ¥1,966,138/QALYs which is greater than that of the more effective strategy of Girls-only routine and catch-up vaccination with 9vHPV

When using only the diseases as indicated in Japan, the cost-effectiveness results still held, but the ICERs did increase somewhat (Table 4). No vaccination vs 4vHPV vaccination had an ICER of ¥1,081,087/QALY whereas 9vHPV + Catch up vs 4vHPV vaccination had an ICER of ¥1,616,830/QALY. Once again, 9vHPV vaccination without catch up was weakly dominated, since the ICER compared to 4vHPV vaccination was ¥2,005,810 /QALY.

**Table 4:** Incremental cost-effectiveness ratio (ICER) of vaccination strategies: sensitivity and scenario analyses

Scenario	Routine 4vHPV vaccination	Routine 9vHPV vaccination	Routine + catch-up 9vHPV vaccination
Base case	975,364	Weakly dominated	1,534,493
Disease consistent with indication			
Cervical disease only	1,349,813	Weakly dominated	1,696,915
+ anogenital warts	1,075,713	Weakly dominated	1,615,134
+ VaIN & VIN^a^	1,075,713	Weakly dominated	1,615,134
+ Other HPV-associated disease			
+ Vulvar cancer	1,072,211	Weakly dominated	1,609,922
+ Vaginal cancer	1,064,445	Weakly dominated	1,597,465
+ Anal cancer	1,037,847	Weakly dominated	1,575,198
+ Head/neck cancer	987,668	Weakly dominated	1,540,035
+ Penile cancer	987,282	Weakly dominated	1,538,902
+ Recurrent respiratory papillomatoses	975,364	Weakly dominated	1,534,493
Perfect coverage (i.e., 100% among 12–16 y)	1,349,381	Weakly dominated	2,200,881
Higher vaccine coverage (i.e., 80% among 12–16 y)	1,096,548	Weakly dominated	1,757,646
Lower vaccine coverage (i.e., 60% among 12–16 y)	861,404	Weakly dominated	1,313,380
Lower adherence to the 3-dose series (i.e., 80%)	777,574	Weakly dominated	1,219,281
Shorter duration of protection for the vaccine (i.e. 10 years for 1-dose, 20 years for 2 doses, and 30 years for 3 doses)	1,507,122	Weakly dominated	1,887,830
Low healthy quality of life weights (i.e., all reduced 20%)	597,871	Weakly dominated	1,009,087
High healthy quality of life weights (i.e., all increased 20%, up to 1)	2,236,971	Weakly dominated	3,089,664
Low disease quality of life weights (i.e., all set to their low values)	591,713.32	Weakly dominated	1,006,573
High disease quality of life weights (i.e., all increased 20%, up to 1)	874,541.87	Weakly dominated	1,360,391
Low disease costs (i.e., all reduced by 20%)	1,096,628.38	Weakly dominated	1,660,622
High disease costs (i.e., all increased by 20%)	854,098.76	Weakly dominated	1,408,364
Lower discount rate per year (0%)	3840	Weakly dominated	6283
Higher discount rate per year (3%)	1,575,569.94	Weakly dominated	2,433,779
Higher discount rate per year (4%)	2,444,532	Weakly dominated	3,740,011
Lower vaccine price (i.e. 9vHPV price decreased 10%)	975,364	Weakly dominated	1,091,729
Higher vaccine price (i.e. 9vHPV price increased 10%)	975,364	Weakly dominated	1,977,259

*HPV* Human papillomavirus, *ICER* Incremental cost-effectiveness ratio, *VIN* Vulvar intraepithelial neoplasia grade 1/2/3, *VaIN* Vaginal intraepithelial neoplasia grade 1/2/3. Routine vaccination with 4vHPV included routine vaccination of 12–16-year old girls with 4vHPV from 2021 onwards; Routine vaccination with 9vHPV included routine vaccination of 12–16-year old girls with 9vHPV; Routine and catch-up vaccination with 9vHPV consisted of routine vaccination of 12–16-year old girls from 2021 onwards and temporary catch-up vaccination of 17–26 years old girls and women with 9vHPV until 2025. All vaccination strategies were combined with cervical cancer screening. ^a^Assumes no vaginal screening is taking place

### Scenario and sensitivity analyses

**Fig. 3:**
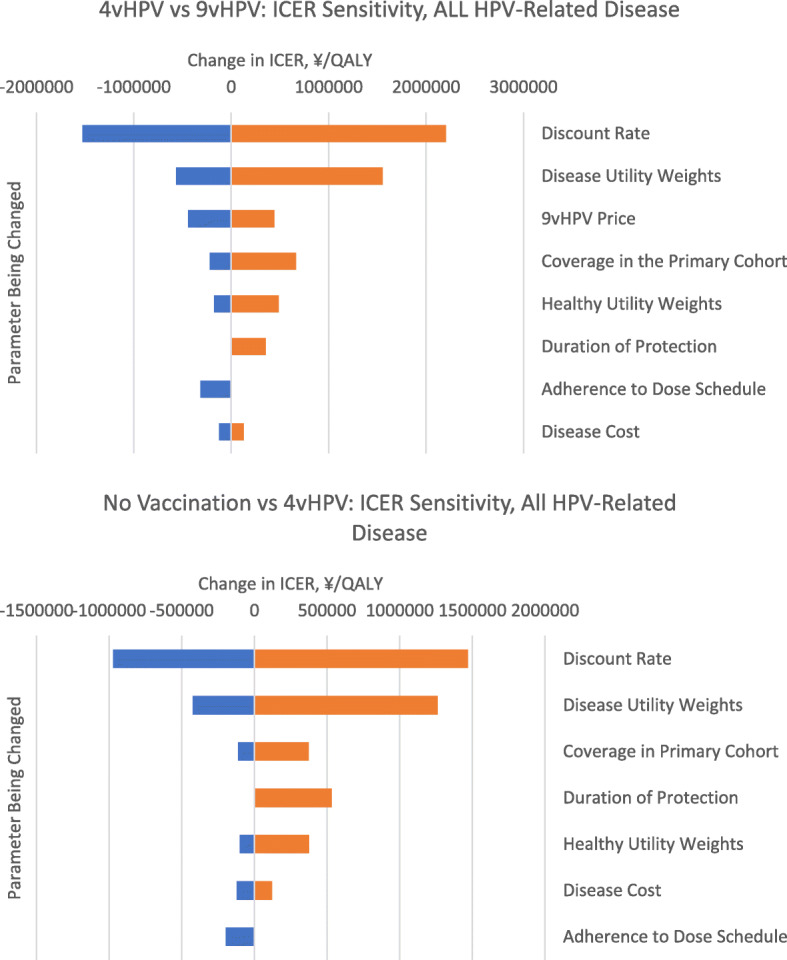
Tornado diagrams for the ICERs, including all HPV-related disease. ICER = incremental cost-effectiveness ratio; HPV = human papillomavirus; ¥ = Japanese Yen. Girls-only vaccination with 4vHPV included routine vaccination of 12–16-year old girls with 4vHPV from 2021 onwards; Girls-only routine and catch-up vaccination with 9vHPV consisted of routine vaccination of 12–16-year old girls from 2021 onwards and temporary catch-up vaccination of 17–26 years old girls and women with 9vHPV until 2025. All vaccination strategies were combined with cervical cancer screening

The above results were very robust in a deterministic sensitivity analysis. We varied scenario-related parameters to quantify this robustness. Detailed outcomes of the sensitivity analyses can be found in the technical appendix, and are summarized here, and in Table 4. Tornado diagrams for the no vaccination vs Routine 4vHPV vaccine ICER, and Routine 4vHPV vaccine vs Routine + Catch up 9vHPV vaccine ICER, when accounting for all disease can be found in Fig. 3. Tornado diagrams for only the diseases indicated in Japan can be found in the technical appendix and are like those shown in Fig. 3.

Results of other scenario or sensitivity analysis are summarized in Table 4. In short, when varying these parameters one-at-a-time, the qualitative results were unchanged; ICERs for no vaccination vs 4vHPV, and for Routine 4vHPV vs Routine + Catch Up 9vHPV, never exceeded ¥5000,000 /QALY. Varying the discount rate had the biggest impact on both ICERs, with disease utilities coming next. Disease costs impacted the ICER less, suggesting that the low ICER was driven more so by quality-of-life benefits, rather than averting treatment costs of disease (Fig. 3).

## Discussion

This analysis showed that vaccination against HPV will have a significant public health impact in Japan, resulting substantial reductions in the cases of 9-valent HPV-caused cervical and non-cervical cancers and anogenital warts. At the expected cost of vaccination, HPV vaccination is highly cost effective at a cost-effectiveness threshold of 5000,000 ¥/QALY. We note that there is not a consensus on the local threshold value for Japan, but the threshold we use here is generally used in cost-effectiveness analyses [35]. We note that there is not a consensus on the local threshold value for Japan, but the threshold we use here is generally used in cost-effectiveness analyses [35]. We note that there is not a consensus on the local threshold value for Japan, but the threshold we use here is generally used in cost-effectivecost-effectiveness analyses [35].

Routine vaccination with a 9-valent vaccine with a catch up to age 26 among girls was found to be the most cost-effective strategy, averting many cancers and deaths and improving the quality of life of both men and women in Japan. Even when considering only the diseases for which the HPV vaccine is indicated in Japan, vaccination was still found to be highly cost effective. We also found that strategies that did not include a catch-up program had higher ICERs than more effective strategies (weakly dominated). These results were robust in sensitivity analyses. We can thus conclude that the 9vHPV vaccine with catch up is the most cost-effective strategy, whether we consider all diseases, or only the diseases as indicated for 9vHPV vaccine in Japan.

Furthermore, these results are consistent with other work that has been published. Konno et al. [36] assessed the clinical impact and cost effectiveness of HPV vaccination at age 12 using a Markov model. The estimated ICER ranged from ¥1.8 million/QALY when vaccinating only 12-year old girls to ¥2.8 million per QALY when vaccinating all 10- to 45-year-old girls and women. Compared to ours, this study did not include herd protection effects and only included vaccine impact on HPV16/18-related cervical cancer and pre-cancer lesions.

Similarly, Yamamoto [15] used a Markov model to estimate the cost effectiveness of HPV vaccination and increased screening coverage to prevent cervical cancer in Japan. They found vaccinating girls aged 11 years and increasing screening coverage to 50% was cost effective when using a willingness-to-pay (WTP) threshold of ¥4.5 million.

Yamabe et al. [14] also assessed the epidemiological and economic impact of the quadrivalent HPV vaccination on 2 strategies, only 12-year old girls vaccination and 12-year old girls vaccination with catch-up program for 12- to 24-year old in Japan using a transmission dynamic model. This study concluded that catch-up program as the most cost-effective strategy, with an ICER of ¥1,205,800/QALY when considering WTP threshold of ¥5000,000/QALY. However, this study did not consider 9vHPV vaccination nor prevention of non-cervical cancers.

Simms et al. [37] quantified the impact of HPV vaccine hesitancy crisis from 2013 to 2019 and the potential health gains if coverage can be restored in Japan by using a dynamic transmission model, the Policy1-Cervix modelling platform. They found that the crisis to date was estimated to result in around 5000 deaths from cervical cancer and many of these deaths could still be prevented if vaccination coverage with extended catch-up can be rapidly restored. Compared with the current study, Simms et al. focused only on the impact of vaccination on cervical cancer incidence and mortality and did not consider prevention of non-cervical cancers and genital warts nor assess the economic impact of HPV vaccine hesitancy crisis.

As with any modeling study, the results are subject to some limitations. First, we assumed that the system had reached an equilibrium state, and as a result cancer incidence is at a constant state before vaccination. However, there is some evidence to suggest that cervical cancer incidence is increasing in Japan [2]. Increasing cancer incidence will tend to increase the value of the vaccine compared with what we assessed here. We also did not consider changes in screening behavior and coverage over the time-horizon. Considering the range of possible options for future screening methodologies, and uncertainty surrounding future adherence to screening guidelines, analyses surrounding screening are beyond the scope of this paper but can be considered in future work. In addition to screening considerations, future research will focus on investigating the impact and value of expanding the accessibility of the vaccine to other populations such as men who have sex with men, boys, and older cohorts.

We have also assumed that there is no coverage of the vaccine before 2020, while in actuality some girls were vaccinated in 2013 [38]. However, the vaccine coverage rate is low and drops off rapidly after 2013. But there are some vaccinated individuals in catchup cohorts that we consider unvaccinated in the model, which could overestimate the value of catch-up vaccination. This does not impact the value of routine vaccination strategies without catchup.

Despite these limitations, our analyses were based on a model that has been extensively validated against existing epidemiological data. The analysis relied heavily on Japanese-specific inputs utilizing data from various sources and published literature. It is reassuring that the model reproduced all baseline incidence of diseases (supplemental material, Figs. 3–16). The approach is also comprehensive in assessing the potential impact of the vaccine on cervical diseases, genital warts, and other diseases that have been linked to HPV 6/11/16/18/31/33/45/52/58 infection such as RRP and cancers of the anus, penis, vagina, vulva, and head and neck among women and men.

## Conclusion

This study showed that vaccination against HPV in Japan has substantial public health and economic benefits. A vaccination program with 9vHPV vaccine and that includes a temporary catch-up program for females up to age 26 was the most cost effective among the strategies that we considered. Such a vaccination program does not only benefit females, but also males with significant reductions in the incidence of HPV-related anogenital warts and cancers among males.

## Supplementary Information

**Additional file 1: Appendix A**. Supplementary material. Supplementary material associated with this article can be found in the Model Technical Report (Technical_report_CEA_G9_Japan.docx).

## Data Availability

All data generated or analyzed during this study are included in this published article [and its supplementary information files].

## Abbreviations

CIN: Cervical intraepithelial neoplasia
HPV: Human papillomavirus
