# Supplementary material for: Public health impact and cost effectiveness of routine and catch-up vaccination of girls and women with a nine-valent HPV vaccine in Japan: a model-based study
Source: BMC Infect Dis. 2021 Jan 6;21:11. doi: 10.1186/s12879-020-05632-0 (PMC7789539; doi:10.1186/s12879-020-05632-0)
Supplement: Supplementary file 1 — Additional file 1: Appendix A. Supplementary material. Supplementary material associated with this article can be found in the Model Technical Report (Technical_report_CEA_G9_Japan.docx). [file 12879_2020_5632_MOESM1_ESM.docx]

**Model Technical Report Accompanying “Public health impact and cost effectiveness of routine and catch-up vaccination of girls and women with a nine-valent HPV vaccine in Japan: a model-based study”**

# Table of Contents

[Table of Contents 2](#_Toc44958110)

[Tables 3](#_Toc44958111)

[Figures 4](#_Toc44958112)

[Abbreviations 6](#_Toc44958113)

[1 Introduction 8](#_Toc44958114)

[1.1 Human papillomavirus 8](#_Toc44958115)

[1.2 Prevention of HPV-related diseases 9](#_Toc44958116)

[1.2.1 Screening 9](#_Toc44958117)

[1.2.2 Vaccination 9](#_Toc44958118)

[1.3 Model-based health and economic evidence of HPV vaccination 10](#_Toc44958119)

[1.4 Study objectives 11](#_Toc44958120)

[2 Methods 12](#_Toc44958121)

[2.1 Model description 12](#_Toc44958122)

[2.2 Model outcomes 14](#_Toc44958123)

[2.3 Model parameters 15](#_Toc44958124)

[2.3.1 Demographics 15](#_Toc44958125)

[2.3.2 Sexual behavior data 16](#_Toc44958126)

[2.4 Screening parameters 18](#_Toc44958127)

[2.4.1 Cervical cancer screening information 18](#_Toc44958128)

[2.5 Natural history of disease 22](#_Toc44958129)

[3 Treatment Patterns 28](#_Toc44958130)

[4 Cancer Mortality 30](#_Toc44958131)

[5 Vaccine Properties 32](#_Toc44958132)

[5.1 Efficacy against infection vs efficacy against disease 32](#_Toc44958133)

[5.2 Efficacy against non-cervical cancers 32](#_Toc44958134)

[5.3 Efficacy for partial vaccine series completion 32](#_Toc44958135)

[6 Vaccination Strategy 36](#_Toc44958136)

[7 Costs 36](#_Toc44958137)

[7.1 Cost of vaccine administration 37](#_Toc44958138)

[7.2 Cost of vaccination 37](#_Toc44958139)

[7.3 Cost per episode of care 37](#_Toc44958140)

[7.4 Cost of screening and diagnostic tests 38](#_Toc44958141)

[8 Health-Related Quality of Life 38](#_Toc44958142)

[9 Calibration Process 40](#_Toc44958143)

[9.1 Data sources 40](#_Toc44958144)

[10 Calibration Results 43](#_Toc44958145)

[11 Sensitivity Analyses 58](#_Toc44958146)

[11.1 Baseline Scenario 58](#_Toc44958147)

[11.2 Removal of CIN2+ 59](#_Toc44958148)

[11.3 Vaccine Doses Adherence 60](#_Toc44958149)

[11.4 Duration of Protection 62](#_Toc44958150)

[11.5 Varied Vaccine Uptake in the Primary Cohort 63](#_Toc44958151)

[11.6 Utilities 65](#_Toc44958152)

[11.7 Discount Rates 69](#_Toc44958153)

[11.8 Price 70](#_Toc44958154)

[References 74](#_Toc44958155)

## Tables

[Table 1 Population Size by Gender 16](#_Toc45542767)

[Table 2: Annual all-cause mortality rates for the general population 16](#_Toc45542768)

[Table 3: Percent of the population in each of the following sexual activity categories 17](#_Toc45542769)

[Table 4: Mean number of sexual partners per year by activity category and gender 17](#_Toc45542770)

[Table 5: Mean number of sexual partners per year by age group and gender 17](#_Toc45542771)

[Table 6: Sexual mixing 18](#_Toc45542772)

[Table 7: Cervical cancer screening information Cervical 20](#_Toc45542773)

[Table 8: Percentage of females screened for cervical cancer in the past year 20](#_Toc45542774)

[Table 9: Diagnostic performance of screening tests by cervical disease (International) 21](#_Toc45542775)

[Table 10: Probability of transmitting HPV infection per sexual partnership, by anatomical site, sex and HPV genotype 22](#_Toc45542776)

[Table 11: Recurrence rate of treated CIN by stage 23](#_Toc45542777)

[Table 12: Rate of cancer progression (Same for all diseases) 23](#_Toc45542778)

[Table 13: Parameters of natural history of cervical disease, 23](#_Toc45542779)

[Table 14:Parameters for Natural History of Vaginal Disease 24](#_Toc45542780)

[Table 15: Parameters for Natural History of Vulvar Disease 25](#_Toc45542781)

[Table 16: Natural History Parameters for Anal Disease 25](#_Toc45542782)

[Table 17: Natural History Parameters for Head and Neck Cancer 26](#_Toc45542783)

[Table 18: Natural history Parameters for Penile Cancer 27](#_Toc45542784)

[Table 19: Women receiving hysterectomy over the course of 1 year 28](#_Toc45542785)

[Table 20: Treatment patterns of all stages of diseases 29](#_Toc45542786)

[Table 21: Annual cancer-associated mortality by site, age and stage 30](#_Toc45542787)

[Table 22: Vaccine efficacy assumptions (international) for HPV-related cancers 34](#_Toc45542788)

[Table 23: Vaccine efficacy assumption (international) for genital warts 35](#_Toc45542789)

[Table 24: Costs of diagnosing and treating diseases caused by HPV infection 37](#_Toc45542790)

[Table 25: Screening and diagnostic tests for cervical and vaginal cancers 39](#_Toc45542791)

[Table 26: Age-specific utilities in healthy population 39](#_Toc45542792)

[Table 27: Utilities in population with HPV-related diseases 40](#_Toc45542793)

[Table 28: Cancer Incidence (cases per 100,000) Data for Females 40](#_Toc45542794)

[Table 29: Cancer Incidence (cases per 100,000) Data for Males 41](#_Toc45542795)

[Table 30 Cervical Cancer Mortality Data 41](#_Toc45542796)

[Table 31: HPV Type Attributions for Male Disease 42](#_Toc45542797)

[Table 32: HPV Type Attribution for Female Disease 42](#_Toc45542798)

[Table 33: Genital Warts Incidence (cases per 100,000) in Japan 43](#_Toc45542799)

[Table 34 Costs/QALYs and ICERs for the baseline scenario. 62](#_Toc45542800)

[Table 35 Costs, QALYs, and ICERs for the baseline scenario without CIN2+. 63](#_Toc45542801)

[Table 36 Costs, QALYs, and ICERs when adherence to 2 and 3 doses is lowered to 90%. 64](#_Toc45542802)

[Table 37 Costs, QALYs, and ICERS when adherence to 2 and 3 doses is lowered to 80%. 65](#_Toc45542803)

[Table 38 Costs, QALYs, and ICERs when the duration of protection is lowered. 66](#_Toc45542804)

[Table 39 costs, QALYs, and ICERs when the uptake in the primary cohort is lowered to 60% 67](#_Toc45542805)

[Table 40 Costs, QALYs, and ICERs when the uptake in the primary cohort is raised to 80% 68](#_Toc45542806)

[Table 41 Costs, QALYs, and ICERs when the uptake in the primary cohort is increased to 100% 68](#_Toc45542807)

[Table 42 Costs, QALYs, and ICERs when the healthy utilities are decreased by 20%. 69](#_Toc45542808)

[Table 43 Costs, QALYs, and ICERS when the disease utilities are increased 20% 70](#_Toc45542809)

[Table 44 Costs, QALYs, and ICERs when the healthy utilities are increased by 20% 71](#_Toc45542810)

[Table 45 Costs, QALYs, and ICERS when the disease utilities are decreased 20% 72](#_Toc45542811)

[Table 46 Cost, QALYS, and ICERs when the discount rate is raised to 4% 73](#_Toc45542812)

[Table 47 Cost, QALYS, and ICERs when the discount rate is raised to 3% 74](#_Toc45542813)

[Table 48 Cost, QALYs, and ICERs when discount rate is decreased to 0% 75](#_Toc45542814)

[Table 49 Costs, QALYs, and ICERs when the price is increased 10% 75](#_Toc45542815)

[Table 50 Costs, QALYs, and ICERs when the price is decreased 10% 76](#_Toc45542816)

[Table 51 Costs, QALYs, and ICERs when the cost of disease is lowered 20% 77](#_Toc45542817)

[Table 52 Costs, QALYs, and ICERs when the cost of disease is raised 20% 78](#_Toc45542818)

## Figures

[Figure 1: A simplified schematic diagram of the pre-vaccination model compartments for HPV infection and disease 14](#_Toc45522978)

[Figure 2. Vaccine coverage 37](#_Toc45522979)

[Figure 3 HPV16 related cervical disease, calibrated fits. Red represents the data, blue the output of the model from 100 posterior draws 46](#_Toc45522980)

[Figure 4 HPV18- related cervical disease, calibrated fits. Red represents the data, blue the output of the model from 100 posterior draws. The dramatic overestimation of CIN2+ prevalence is investigated in sensitivity analyses (Section 11) 47](#_Toc45522981)

[Figure 5 HPV31 related cervical disease, calibrated fits. Red represents the data, blue the output of the model from 100 posterior draws. 48](#_Toc45522982)

[Figure 6 HPV16 related anal cancer for males and females, calibrated fits. Red represents the data, blue the output of the model from 100 posterior draws. 49](#_Toc45522983)

[Figure 7 HPV18 related anal cancer for men and women, calibrated fits. Red represents the data, blue the output of the model from 100 posterior draws. Sparsity is due to repeatedly sampling parameter sets. The likelihood here was tightly peaked, so when weighted resampling of parameter sets was done to create an approximate posterior, the MLE was repeatedly sampled most of the time. 50](#_Toc45522984)

[Figure 8 HPV31+ related anal cancer in men and women. Due to the relatively small incidence, the model calibration would admit the disease-free state. In this case we opted to just apply the MLE, which is what is plotted here. 51](#_Toc45522985)

[Figure 9 HPV16 related oropharyngeal cancer among men and women, calibrated fits. Red represents the data, blue the output of the model from 100 posterior draws 52](#_Toc45522986)

[Figure 10 HPV18 related oropharyngeal cancer in males and females, calibrated fit. Due to the relatively small incidence, the model calibration would admit the disease-free state. In this case we opted to just use the MLE, which is what is plotted here. 53](#_Toc45522987)

[Figure 11 HPV31 related oropharyngeal cancer in males and females, calibrated fits. Red is the data, blue are the model outputs from 100 posterior draws. 54](#_Toc45522988)

[Figure 12 HPV related penile cancer in men. Red is the data, blue are the model outputs from 100 posterior draws. 55](#_Toc45522989)

[Figure 13 HPV related vaginal cancer. Red is the data, and blue is the output of the model for 100 posterior draws. Sparsity in the middle figure is due to a tightly peaked likelihood, so that weighted resampling predominately drew the MLE. 56](#_Toc45522990)

[Figure 14 HPV related vulvar cancer in women. Red is the data, and blue is the output of the model for 100 posterior draws. Due to the relatively small incidence, the model calibration would admit the disease-free state for HPV18 and HPV31+. In this case we opted to just use the MLE, which is what is plotted here. 57](#_Toc45522991)

[Figure 15 HPV 6 and 11 attributable genital warts. Data is in orange, and the output from the model for 100 posterior draws in in blue. 58](#_Toc45522992)

[Figure 16 JORRP incidence. Data is in orange, and model output for 100 posterior draws is shown in blue. 59](#_Toc45522993)

[Figure 17 ICER sensitivities when only considering cervical disease and genital warts. 60](#_Toc45522994)

[Figure 18 ICER sensitivities when all diseases are included. 61](#_Toc45522995)

# Abbreviations

AC Anal cancer

ACIP Advisory Committee on Immunization Practices

CC Cervical Cancer

C-E Cost-Effective

CEA Cost-Effectiveness Analysis

CHMP Committee for Medicinal Products for Human Use

CIN Cervical Intraepithelial Neoplasia

CIS Carcinoma In Situ

EMA European Medicine Agency

EU Europe

EUROCARE European Cancer Registry

EUROGIN European Research Organisation on Genital Infection and Neoplasia

GUM Genito-Urinary Medicine

GW Genital Warts (condyloma acuminata)

FDA Food and Drug Administration of the United States

H&N Head and Neck

HEOR Health Economics and Outcomes Research

HES Hospital Episodes Statistics

HIV Human immunodeficiency virus

HPA Health Protection Agency

HPV Human Papillomavirus

ICER Incremental Cost-Effectiveness Ratio

IPV International Papillomavirus conference

JCVI Joint Committee on Vaccination and Immunisation

MSM Men who have Sex with Men

NATSAL National Survey of Sexual Attitudes and Lifestyles

NHS National Health Service

NICE National Institute for Health and Care Excellence

ONS Office for National Statistics

OR Odds Ratio

Pap Papanicolaou Smear

PC Penile cancer

QALY Quality-Adjusted Life Year

QoL Quality of Life

RRP Recurrent Respiratory Papillomatosis

SPMSD Sanofi Pasteur MSD

TNM Tumour-Node-Metastasis

TTO Time trade-off

UBC United BioSource Corporation

US United States

VaC Vaginal Cancer

VaIN Vaginal Intraepithelial Neoplasia

VC Vulvar Cancer

VIN Vulvar Intraepithelial Neoplasia

US United States of America

# Introduction

In this report, we present technical details and some results from the model used to assess the health and economic impact of a 9-valent vaccine (9vHPV) in Japan. To do so, an existing updated mathematical dynamic disease transmission model developed was used to simulate the natural history of HPV-related infections (i.e., transmission, disease progression/regression) and included economic parameters over a 100-year time horizon [1, 2]. For this analysis, population characteristics, screening & treatment patterns, vaccination assumptions, vaccination strategies, costs and health utilities were obtained for Japan setting following a detailed data collection––the model was informed with Japanese-specific parameters when available.

In cases where the specific data were not available, alternative values from other comparable countries were used. Additional epidemiological data was collected in order to calibrate the model to data from Japan. To adequately capture the epidemiology of all the diseases, the model was calibrated (the process of finding the best model fit for a set of input values chosen from specified plausible ranges) to match current published data on incidence/prevalence for each disease considered.

## Human papillomavirus

Human papillomavirus (HPV) is the major causal organism that infects epithelial tissues of the skin, cervix, anus, mouth and throat.[3-5] Transmission of HPV largely occurs through sexual contact (vaginal anal and oral) or vertical transmission from mother to child during pregnancy and/or delivery.[6-8] It is estimated that 8 out of 10 people will be infected with HPV at some point in their lives, however, because a high majority do not cause cancer, are asymptomatic and the body’s immune system is able to fight them off, infected people may never know they ever had it.[9, 10] Recent global estimates indicate that there were in excess of 630,000 new cases (570,000 in females; 60,000 in males) of cancers attributable to HPV in 2018.[11] The most common HPV-related cancers worldwide were cervical cancer (528,000 cases), followed by Head and Neck (H&N) cancer (37,500 cases), anal cancer (35,000 cases), penile cancer (13,000 cases), vaginal cancer (12,000 cases) and vulvar (8,500 cases) cancer.[12]

In Japan, cervical cancer is the second most cancer in women after breast cancer. Based on the National Cancer Registry[13], there were 11,000 new cases and 2,800 deaths were reported, most of which were considered as HPV-related. With regard to other cancers, Japan specific data on incidence was collected from GLOBOCAN[14].

More than 200 different types of HPV have been identified. Types 6 and 11 affect the genital area and cause 85% of genital warts cases.[15, 16] However they (6 and 11) do not cause cervical cancer. As a result, they are considered low-risk HPV. High risk (HR) HPV types are those known to increase the risk of developing cancers. The HR HPV group includes types 16, 18, 31 and 45––types 16 and 18 are known to account for 70% of high-grade cervical intraepithelial neoplasia (CIN) and invasive cervical cancer, while types 31 and 45 are the next most important contributors[17]. The National Cancer Institute estimates that HPV causes virtually all cervical cancers, over 90% of anal cancers, 70% of oropharyngeal cancers, 75% of vaginal cancers, 70% of vulvar cancers, and 60% of penile cancers.[18]

## Prevention of HPV-related diseases

### Screening

Because of the asymptomatic nature of HPV infections, the most effective way of preventions is identification and adequate treatment. Therefore, regular screening to detect the infection is an important first step. As a result, many developed counties have introduced cervical screening programs aimed at detecting the precursors, i.e. cervical intraepithelial neoplasia grade 2 (CIN2), grade 3 (CIN3) and atypical glandular cells and treat these lesions at an early stage before evolving to cancer. These programs use two main types of cervical screening––cytology-based screening (also known as a smear test or Pap screen) and HPV DNA testing. The cytological screening identifies abnormal cells from the cervix that may develop into cancer if left untreated.[19] HPV testing is used to look for the presence of high-risk HPV types in cervical cells and to detect HPV infections that cause cell abnormalities sometimes before these are evident.[20]

A Cervical Screening Program in Japan was introduced for women of age 20 years old and up by Ministry of Health, Labor and Welfare (MHLW) in 2004. Eligible women are recommended to receive cervical screening test every other year. Screening rate is still low, about 35% to 40%[21] .

### Vaccination

HPV vaccines are designed to prevent HPV infection and HPV-related disease. Currently, there are two commercially available vaccines in Japan:

- Gardasil^®^ (MSD): a recombinant vaccine with protection against HPV 6, 11,16 and 18, indicated in girls and boys from 9 years old for the protection of premalignant genital lesions (cervical, vulvar and vaginal), premalignant anal lesions, cervical cancers, anal cancers and genital warts[22, 23]
- Cervarix^®^ (GlaxoSmithKline): a vaccine with protection against HPV 16 and 18, indicated in women and girls aged from 9 years old for the protection of premalignant genital (cervical, vulvar and vaginal) lesions and cervical cancer[24]

Gardasil^®^ and Cervarix^®^ have been shown to induce a strong immune response against the target HPV strains.[25] Both Gardasil^®^ and Cervarix^®^ were first approved in a 3-dose regimen.

Gardasil 9^®^ (MSD): a recombinant vaccine with protection against nine HPV types (6, 11, 16, 18, 31, 33, 45, 52 and 58) has been available in many countries since 2014 [26]. In Japan this 9-valent vaccine is licensed under the name Silgard9.

The 9-valent vaccine (Silgard9) is expected to provide coverage against most high-risk carcinogenic HPV types. Recent data have confirmed that types 16, 18, 31, 33, 45, 52 and 58 are amongst those most frequently detected.[27] In the per protocol analysis of a trial conducted on 14,215 women aged between 16 and 25 years comparing Silgard9 to Gardasil, Silgard9 was shown to offer a 96.6% reduction in the combined incidence of high-grade CIN 2/3+, VIN 2/3+ and VaIN 2/3+, caused by HPV types 31, 33, 45, 52 and 58. Persistent infections of 6-month or longer duration related to HPV 31, 33, 45, 52 or 58 were reduced by 96.0%. When the analysis was restricted to women not infected on day one, a significant 42% reduction compared to Gardasil for disease related to all HPV types was seen reflecting the benefit of reduced infection from the additional five types in Silgard9 (31, 33, 45, 52 and 58). Immunogenicity induced by the vaccine was very strong with virtually 100% seroconversion of all per protocol patients within 1 month of the third dose. Geometric mean titres were 50-fold higher for each of the five new HPV types for Silgard9 compared to Gardasil and similar for the four types in common [28-30]. Comparisons with historic placebo have also showed high efficacy against cervical disease caused by the nine types [31].

## Model-based health and economic evidence of HPV vaccination

In Japan, a few studies assessing the cost-effectiveness of HPV quadrivalent and bivalent vaccines have been published[32, 33] and Yamabe et.al[32] assessed the cost-effectiveness for 4-valent HPV vaccine by using the previous version of the current model. However, to our knowledge, no analysis of the cost-effectiveness for the 9-valent HPV vaccine has been performed yet.

## Study objectives

This report provides details on the model used to assess the health and economic outcomes of the implementing a universal 9-valent (HPV 6/11/16/18/31/33/45/52/58) vaccine program for girls aged 12 to 16 compared to the current clinical practice with a quadrivalent (HPV 6/11/16/18) vaccination of the same age cohort of girls in Japan. In addition, the model aims to assess the incremental public health impact at population level of a vaccination program with a 9-valent HPV vaccine in Japan compared to the current program with a quadrivalent HPV vaccine. Results of alternative input parameters are described in the sensitivity analyses.

# Methods

## Model description

The platform used to estimate the cost-effectiveness of vaccine against Human Papillomavirus is an integrated HPV disease transmission model that accounts for herd protection effects, with a 100-year time horizon. The model was first built to assess the cost-effectiveness of the original quadrivalent HPV vaccine against HPV types 6, 11, 16, and 18. The model, built in Mathematica, initially included two HPV-related diseases, cervical diseases (females) and genital warts (females and males). It was first published in the US and then adapted to many settings including Japan[32]. This model evaluated epidemiological and economic consequences of the quadrivalent prophylactic vaccine administration against HPV 6/11/16/18 with cervical cancer screening. The use of a dynamic model structure allows herd immunity to be taken into account (i.e. indirect protection of non-vaccinated people by the vaccinated people due to a reduction of virus circulation). A detailed description of the original model including the model structure (relating to the natural history of HPV-related infections), stratifications, input parameters (demographic and epidemiological), model calibration and validation, impact of vaccination and economic model was published in Elbasha et al. (2007).[2, 34]

In 2019, the model was updated in order to consider more up-to date published data on the natural history of HPV-related infections and diseases as well as Gardasil vaccine efficacy. Model structure was redefined to take all HPV-related diseases into account (i.e., cervical cancer, vaginal cancer, vulvar cancer, anal cancer, penile cancer, and the associated precancerous lesions, head & neck cancer (not otherwise specified), genital warts, and recurrent respiratory papillomatosis (juvenile- and adult-onset) and the option to vaccinate boys.^122^ It was also extended to investigate the 9-valent HPV vaccine, to evaluate the cost-effectiveness of vaccination to provide protection against HPV types 6, 11, 16, and 18 and 5 new HPV types: 31, 33, 45, 52, and 58.

In this model, the structure was formulated as a system of ordinary differential equations. These equations were programmed and numerically solved using the NDSolve function in Mathematica^®^ 12 (Wolfram Research, Champaign, IL). To extend the original model, compartments and differential equations were added to account for infections and diseases attributable to HPV genotypes 31, 33, 45, 52, and 58. Altogether, the model accounted for the transmission dynamics of all nine HPV types covered by 9vHPV.

This model version was presented in ACIP meeting in February 2019 and is used for the analyses presented in the present dossier. This model version was adapted to the Japan setting.

The model includes several modules that can be summarized as follows:

- Population characteristics: demographics (i.e., population size), and behavior (i.e., sexual activity through sexual partnership)
- Screening strategies: screening coverage rate, cytological test and colposcopy characteristics (for cervical and vaginal cancers only)
- Natural history of the disease/infection: herd immunity or transmission of infection
- Disease and patterns: disease management and/or clinical pathways (from diagnosis to treatment)
- Vaccination assumptions: such as vaccine efficacy, duration of protection, degree of protection, compliance/adherence
- Vaccination strategies: gender type, uptake/coverage rate
- The model allows to assess the impact of a bivalent, quadrivalent and 9-valent HPV vaccines
- Costs: Cost information on diagnosis and treatment, defined as an "episode of care" for a specific disease state. An "episode of care" is defined to begin with the diagnosis and end with the resolution of the case. It entails the costs of diagnosing and treating the case. Cost of vaccination including dose and administration
- Health utilities: utility values for the general population and for each disease stage of the different HPV-related diseases

The model structure is depicted in Figure 1.

*regression*

*clearance*

*infection*

*persistent infection*

*progression*

*infection*

*regression*

*recovery*

*transient*

*infection*

*clearance*

Recovered

without Seroconversion

Susceptible

Persistently Infected

Disease

Recovered

with Seroconversion

*waning*

*progression*

*recovery*

Infectious

*waning*

Figure 1: A simplified schematic diagram of the pre-vaccination model compartments for HPV infection and disease

## Model outcomes

Several epidemiological and economic output measures were used to assess the epidemiological impact and cost-effectiveness of the vaccination strategy:

- Diseases included in the model:
  - Cervical cancer
  - CIN 1, 2 and 3
  - Vaginal cancer
  - Vulvar cancer
  - Anal cancer
  - Genital warts
  - Penile cancer (HPV-associated disease not included in the label)
  - H&N cancer (HPV-associated disease not included in the label)
  - RRP (HPV associated disease not included in the label)
- Epidemiological outputs included:
  - Estimated HPV 16 / 18 / 31 / 33 / 45 / 52 / 58-related HPV infection prevalence among females and males
  - Estimated HPV 6/11-related HPV infection prevalence among females and males
  - Cases of HPV 16 / 18 / 31 / 33 / 45 / 52 / 58-related CIN, cervical cancers and cervical cancer deaths
  - Cases of HPV 16/18-related vaginal, vulvar, penile, anal and H&N cancers and cancer deaths
  - Cases of HPV 6/11-related genital warts cases, RRP and RRP deaths
- Economic outputs included total costs, survival, quality-adjusted survival, and incremental cost per quality-adjusted life year (QALY) ratios;

The total costs of each strategy included the cost of vaccination, cost of cytology screening, cost of following false-positive results, and total cost of managing detected precancerous lesions (CIN, VaIN, VIN), cervical cancer, vaginal and vulvar cancers, and genital warts.

The quality-adjusted survival time (i.e., QALYs) was measured by weighting the survival time by the health-utility weights (or quality-of-life adjustment weights) associated with each health state and then integrating the sum of all these adjusted health states over the planning horizon (i.e., 100 years).

The incremental cost-effectiveness ratio (ICER) was measured as the incremental cost difference between the two strategies divided by the incremental QALY difference between the two strategies.

## Model parameters

### Demographics

Population size (Table 1) and all-cause mortality rates (Table 2) were obtained from government statistics[35].

Table 1 Population Size by Gender

| **Gender Group** | **Population size** |
| --- | --- |
| Male | 65,167,269 |
| Female | 61,765,503 |
| Total | 126,932,772 |

Table 2: Annual all-cause mortality rates for the general population

| **Age group (years)** | **Males (percent)** | **Females (percent)** |
| --- | --- | --- |
| <1 | 0.092 | 0.087 |
| 1-8 | 0.013 | 0.011 |
| 9-11 | 0.008 | 0.005 |
| 12 | 0.008 | 0.004 |
| 13 | 0.013 | 0.01 |
| 14-17 | 0.021 | 0.009 |
| 18 | 0.034 | 0.014 |
| 19 | 0.034 | 0.015 |
| 20-24 | 0.046 | 0.019 |
| 25-26 | 0.046 | 0.023 |
| 27-29 | 0.049 | 0.024 |
| 30-34 | 0.06 | 0.031 |
| 35-39 | 0.077 | 0.043 |
| 40-44 | 0.115 | 0.071 |
| 45-49 | 0.187 | 0.108 |
| 50-54 | 0.3 | 0.165 |
| 55-59 | 0.487 | 0.236 |
| 60-64 | 0.802 | 0.348 |
| 65-69 | 1.322 | 0.547 |
| 70-74 | 2.007 | 0.839 |
| 75-79 | 3.207 | 1.485 |
| 80-84 | 5.686 | 2.981 |
| >85 | 14.933 | 10.758 |

### Sexual behavior data

Age specific sexual behavior data came from two key sources. For individuals under the age of 24 mean number of partners came from the reports of the Japanese Association for Sex Education[36]. For mean number of partners for individuals over 25 years and older, population distribution by sexual activity, and mean number of partners by activity, data was collected from the HIV & SEX in Japan Survey. These data are given in Table 3-Table 5.

Table 3: Percent of the population in each of the following sexual activity categories

| **Category** | **Male** | **Female** |
| --- | --- | --- |
| Low (mean number of sexual partners/year: ≤ 1) | 76.7% | 89.1% |
| Medium (mean number of sexual partners/year: 2-4) | 19.0% | 9.5% |
| High (mean number of sexual partners/year: 5+) | 4.20% | 1.30% |

Table 4: Mean number of sexual partners per year by activity category and gender

| **Sexual activity category** | **Males**  **(number)** | **Females (number)** |
| --- | --- | --- |
| Low (mean number of sexual partners per year: 0-1) | 1.0 | 1.0 |
| Medium (mean number of sexual partners per year: 2-4) | 3.1 | 2.6 |
| High (mean number of sexual partners per year: 5+) | 5.0 | 5.0 |

Table 5: Mean number of sexual partners per year by age group and gender

| **Age Group** | **Male** | **Female** |
| --- | --- | --- |
| **13-14** | 1.28 | 1.18 |
| **15-17** | 1.08 | 1.09 |
| **18-24** | 1.10 | 1.08 |
| **25-34** | 1.70 | 1.13 |
| **35-44** | 1.72 | 1.05 |
| **45-54** | 1.17 | 1.04 |
| **55<** | 1.17 | 1.04 |
| **13-14** | 1.28 | 1.18 |
| **15-17** | 1.08 | 1.09 |
| **18-24** | 1.10 | 1.08 |
| **25-34** | 1.70 | 1.13 |
| **35-44** | 1.72 | 1.05 |
| **45-54** | 1.17 | 1.04 |
| **55<** | 1.17 | 1.04 |

The amount of sexual mixing among members of different age cohorts (a value between 0 and 1 with 0 representing no mixing, and 1 representing maximum mixing) and the amount of sexual mixing among members of different sexual activity groups required in the model were extracted from two sources. The mixing between age groups comes from the Japanese Association for Sex Education, and the mixing among activity groups, and in older age groups comes from the technical report accompanying the Manuscript “Impact of Vaccinating Boys and Men against HPV in the United States".[1] Sexual mixing values are reported in Table 6.

Table 6: Sexual mixing

| **Ages** | **Sexual mixing** |
| --- | --- |
| Among members of different age cohort | |
| Between debut and cessation | 0.40 |
| After cessation | 0.10 |
| Among members of different sexual activity groups | 0.50 |

## Screening parameters

### Cervical cancer screening information

Information on the percent of females who receive a follow-up screening test was gleaned from the Japan cancer society[37]. The percentage of women who will never receive a gynecological screening came from the research from a collaboration between MTI Ltd. and Think Pearl[38]. In this data, among women older than 50 years 16.7% had never had a gynecological screening in their lifetime. This number was chosen to represent the percentage of women who will never get screened within their lifetime. These screening parameters are summarized in Table 7.

Setting the annual screening rate by age presented some unique challenges for Japan. An overview of the national life basic survey provided the proportion of women screened in the last year that is found in Table 8. The percent of women who are screened in the last year represents the best data for the model, which screens continuously over time. However, given the low rates of regular screening in Japan, it was found that this measurement was not a sufficient proxy for regular screening for females in their 20’s and 30’s. If we assumed that this rate represented regular screening, then the model was unable to reproduce the incidence of cervical cancer for females in their 20’s, suggesting that the screening rate was too high. To account for this, we scaled down the screening rate for females in their 20’s and 30’s by a proportion that was calibrated to best match the data for HPV16 related cancer. This proportion was then imputed to the other HPV types, since there is no expectation that screening rates will differ among populations with different HPV types. This scaling value was found to be .2498. To validate this value, we compared this to the results of the screening study from the Japan Cancer Society[37]. The reported annual screening rate is then scaled down to .2650*.2498=.0662. Over the course of their 20’s, assuming approximately equal population sizes and that 100%-6.62%= 93.38% are not screened in a given year, we expect that will be the proportion of females who are never screened in their 20’s to be the average of the expected proportions unscreened for each age:

$$\frac{1}{10}\sum_{n=1}^{10} {.9338}^{n}= .6991$$

From the Japan Cancer Society, it was suggested that 82.3% of females go unexamined in their 20’s. A similar calculation for screening for ages 30-39 shows that, assuming .6991 are leaving their 20’s unscreened, the model predicts .3536 to be the proportion of women in their 30’s who are never examined. This compares to an estimated .653 from the Japan Cancer Society. Hence, we are somewhat overestimating screening (that is, our estimate of the proportion of women who are never screened are underestimates). It is worth noting that, when compared to the study from the MTI Ltd. and Think Pearl[38], our estimates of the proportion of women never screened in their 20’s and 30’s are overestimates. The annual screening probabilities used in the model are presented in Table 8.

Table 7: Cervical cancer screening information Cervical

| **Cancer screening information** | **Value females (percent)** |
| --- | --- |
| Percent of females receiving a follow-up screening test after abnormal cytology result | 83.8 |
| Percent of females never receiving a gynecological screening in their lifetime. | 16.7 |

Table 8: Percentage of females screened for cervical cancer in the past year

| **Age group (years)** | **Value Females (percent)** | **Scaled Values** |
| --- | --- | --- |
| <1 | 0.00% | 0.00% |
| 1-8 | 0.00% | 0.00% |
| 9-11 | 0.00% | 0.00% |
| 12 | 0.00% | 0.00% |
| 13 | 0.00% | 0.00% |
| 14-17 | 0.00% | 0.00% |
| 18 | 0.00% | 0.00% |
| 19 | 0.00% | 0.00% |
| 20-24 | 26.50% | 6.62% |
| 25-26 | 26.50% | 6.62% |
| 27-29 | 26.50% | 6.62% |
| 30-34 | 51.60% | 12.89% |
| 35-39 | 51.60% | 12.89% |
| 40-44 | 52.40% | 52.40% |
| 45-49 | 52.40% | 52.40% |
| 50-54 | 45.70% | 45.70% |
| 55-59 | 45.70% | 45.70% |
| 60-64 | 33.30% | 33.30% |
| 65-69 | 33.30% | 33.30% |
| 70-74 | 21.50% | 21.50% |
| 75-79 | 21.50% | 21.50% |
| 80-84 | 7.90% | 7.90% |
| >85 | 7.90% | 7.90% |

Table 8 summarizes the values of the parameter related to the diagnostic performance of cytological screening and colposcopy for cervical disease and/or CIN stage. The specificity and sensitivity of cytological screening and colposcopy for cervical disease are kept the same as the US data.

Table 9: Diagnostic performance of screening tests by cervical disease (International)

| **Parameter** | **Cervical disease** | **CIN 1** | **CIN 2** | **CIN 3** |
| --- | --- | --- | --- | --- |
| Cytology specificity | 0.94 | Not considered | | |
| Colposcopy sensitivity | 0.96 |  |  |  |
| Colposcopy specificity | 0.48 |  |  |  |
| Cytology sensitivity | Not considered | 0.28 | 0.59 | 0.59 |

Source: Elbasha and Dasbach[39]

Because the Japan has no vaginal cancer screening program, the percentage of females receiving regular vaginal cancer screening will be set to 0. However, the model does account for yearly screening of vaginal cancer survivors. While this does incur some costs, due to the low incidence of vaginal cancer, this has no significant impact on results.

## Natural history of disease

Tables 9–17 show the calibrated values of model parameters (see Section 9) , in particular those that are most relevant to the dynamics of HPV infection e.g. transmission and HPV infection progress, recovery, and natural immunity. Values of these different parameters are similar to US values or were estimated through a calibration process.

Table 10: Probability of transmitting HPV infection per sexual partnership, by anatomical site, sex and HPV genotype

| **Site** | **Transmission** | **HPV 16** | **HPV 18** | **HPV 6** | **HPV 11** | **HPV type 31, 33, 45, 52 or 58** |
| --- | --- | --- | --- | --- | --- | --- |
| Cervical | To males (scale) | .2534,  (.2301,.3275) | .3194,  (.3193,.3376) | - | - | .3218,  (.2951,.3228) |
|  | To females | .2875,  (.2460,.3814) | .3898,  (.3842,.3958) | - | - | .4646,  (.4585,.4689) |
| Vaginal | To males (scale) | .6086,  (.2759, .7308) | .9009,  (.9007,.9029) | - | - | .3621,  (.3287,.5007) |
|  | To females | .3151,  (.2810, .4571) | .3885,  (.3884, .3959) | - | - | .3261,  (.1697, .3346) |
| Vulvar | To males (scale) | .2556,  (.2554,.7244) | .0580 | - | - | .5128 |
|  | To females | .4210,  (.1834,.4211) | .5344 | - | - | .2189 |
| Anal | To males (scale) | .4664,  (.2892,.4966) | .4895,  (.3050,.5622) | - | - | .1881 |
|  | To females | .1347,  (.1329,.2118) | .2141,  (.1999,.3449) | - | - | .0258 |
| Head and Neck | To males (scale) | .4750,  (.3465,.5420) | 1 | - | - | .4573,  (.4531,.4574) |
|  | To females | .2059,  (.1965,.2124) | .0660 | - | - | .1852,  (.1839,.1853) |
| Penile | To males (scale) | .4382,  (.1708,.5686) | .3223,  (.3200,.4484) | - | - | 0.1689,  (.0936,.6510) |
|  | To females | .2160,  (.1067,.3421) | .1888,  (.1483,.1899) | - | - | .4280,  (.0071,.4281) |
| Genital Warts | To males | - | - | .8310,  (.6943,.8311) | .7602,  (.2823,.7603) | - |
|  | To females | - | - | 2017,  (.1842,.2237) | .2715,  (.2674,.7068 | - |

Table 11: Recurrence rate of treated CIN by stage

| **Stage** | **Rate** |
| --- | --- |
| CIN 1 | 0.05 |
| CIN 2 | 0.05 |
| CIN 3 | 0.05 |

Table 12: Rate of cancer progression (Same for all diseases)

| **Progression** | **Rate** |
| --- | --- |
| Local to regional | 0.1 |
| Regional to distant | 0.3 |

Table 13: Parameters of natural history of cervical disease,

| **Parameters (References)** | **HPV 16** | **HPV 18** | **HPV type 31, 33, 45, 52 or 58** |
| --- | --- | --- | --- |
| Fraction of persistent cervical HPV infections* | .1828,  (.1735, .2048) | .1977,  (.1838, .2069) | .1075,  (.1000,.1149) |
| Clearance rate of cervical HPV infections* | | |  |
| Male | .2709, (.2355,.4469) | .6090,  (.6051, .6303) | .8275,  (.8196, .8277) |
| Female | .6736,  (.6324, 1.1312) | 1.8056,  (1.7042,1.8457) | .5029,  (.4693, .5030) |
| Fraction of people seroconvert following a cervical HPV infection* | | |  |
| Male | .1131,  (.0975, .1186) | .1088,  (.0986, .1090) | .1222,  (.1213, .12247) |
| Female | .5714,  (.4740, .6441) | .5803,  (.5594, .5804) | .6398,  (.6390, .6554) |
| Degree of protection against cervical HPV infections provided by natural immunity following seroconversion* | | |  |
| Male | .1701,  (.1390, .2952) | .1816,  (.1802, .1908) | .2300,  (.2174, .2817) |
| Female | .2910,  (.2602, .2911) | .3885,  (.3884, .4096) | .3587,  (.2884, .3642) |
| *From model calibration | | |  |
|  | | | |

Table 14:Parameters for Natural History of Vaginal Disease

| **Parameters (References)** | **HPV 16** | **HPV 18** | **HPV type 31, 33, 45, 52 or 58** |
| --- | --- | --- | --- |
| Fraction of persistent vaginal HPV infections* | .0510,  (.0316, .0772) | .0144,  (.0130, .0150) | 0.0844,  (.0788, .1786) |
| Clearance rate of vaginal HPV infections** | | |  |
| Male | 0.486582 | 0.486582 | 0.486582 |
| Female | 0.395504 | 0.395504 | 0.395504 |
| Fraction of people seroconvert following a vaginal HPV infection* | | |  |
| Male | 0.0808,  (.0724, .0889) | .0520, (.0520, .0530) | .0897,  (.0784, .0952) |
| Female | .6678,  (.5247, .6680) | .7000,  (.7000, .7000) | .6316,  (.5667, .6317) |
| Degree of protection against vaginal HPV infections provided by natural immunity following seroconversion* | | |  |
| Male | .2039,  (.0797, .2681) | .1213,  (.0921, .1214) | .1711,  (.1468, .2085) |
| Female | .2706,  (.2705,.4019) | .4517,  (.4510, .4518) | .4046,  (.3549, .4123) |
| *From Model calibration  ** From Model calibration for the US | | | |

Table 15: Parameters for Natural History of Vulvar Disease

| **Parameters (References)** | **HPV 16** | **HPV 18** | **HPV type 31, 33, 45, 52 or 58** |
| --- | --- | --- | --- |
| Fraction of persistent vulvar HPV infections* | .0823,  (.0803, .1925) | 0.2500 | 0.1419 |
| Clearance rate of vulvar HPV infections** | | |  |
| Male | 0.583899 | 0.583899 | 0.583899 |
| Female | 0.474606 | 0.474606 | 0.474606 |
| Fraction of people seroconvert following a vulvar HPV infection* | | |  |
| Male | .0759,  (.0686,.0890) | .0677 | .0520 |
| Female | .4926,  (.4852,.5000) | .6590 | .7940 |
| Degree of protection against vulvar HPV infections provided by natural immunity following seroconversion* | | |  |
| Male | .1813,  (.0665,.1814) | .1652 | .2911 |
| Female | .3207,  (.2952,.3500) | .2382 | .4945 |
| *From Model calibration  ** From Model calibration for the US | | | |

Table 16: Natural History Parameters for Anal Disease

| **Parameters (References)** | **HPV 16** | **HPV 18** | **HPV type 31, 33, 45, 52 or 58** |
| --- | --- | --- | --- |
| Fraction of persistent Anal HPV infections* | | | |
| Male | .1301,  (.0724,.1302) | .0782,  (.0528,.1646) | .2500 |
| Female | .0910,  (.0628,.1701) | .1248,  (.0333,.1721) | 0.1187 |
| Clearance rate of anal HPV infections** | | |  |
| Male | 0.520000 | 0.882000 | 0.630000 |
| Female | 0.234000 | 0.882000 | 0.630000 |
| Fraction of people seroconvert following an anal HPV infection* | | |  |
| Male | .1323,  (.0856,.1403) | .1288,  (.0133, .1945) | .0600 |
| Female | .4775,  (.4095,.6511) | .4625,  (.3263,.7044) | .2539 |
| Degree of protection against anal HPV infections provided by natural immunity following seroconversion* | | |  |
| Male | .2443,  (.2057,.3194) | .4350,  (.1478,.4351) | .0451 |
| Female | .4942,  (.2863,.7015) | .8094,  (.1838,.8095) | .1388 |
| *From Model calibration  ** From Model calibration for the US | | | |

Table 17: Natural History Parameters for Head and Neck Cancer

| **Parameters (References)** | **HPV 16** | **HPV 18** | **HPV type 31, 33, 45, 52 or 58** |
| --- | --- | --- | --- |
| Fraction of persistent head and neck HPV infections* | | | |
| Male | .1258,  (.1152,.1726) | .2312 | .0925,  (.0924,.0945) |
| Female | .0898,  (.0862,.0989) | .2402 | .1007,  (.0997,.1012) |
| Clearance rate of head and neck HPV infections** | | |  |
| Male | 0.520000 | 0.630000 | 0.630000 |
| Female | 0.520000 | 0.630000 | 0.630000 |
| Fraction of people seroconvert following a head and neck HPV infection* | | |  |
| Male | .1008,  (.0858,.1067) | .0520 | .0929,  (.0917,.0944) |
| Female | .5797,  (.5433,.6484) | .7182 | .5551,  (.5525, .5552) |
| Degree of protection against head and neck HPV infections provided by natural immunity following seroconversion* | | |  |
| Male | .1665,  (.1527,.1930) | .0385 | .2077,  (.2068, .2085) |
| Female | .3621,  (.3432,.3726) | .2001 | .3429,  (.3428,.3456) |
| *From Model calibration  ** From Model calibration for the US | | | |

Table 18: Natural history Parameters for Penile Cancer

| **Parameters (References)** | **HPV 16** | **HPV 18** | **HPV type 31, 33, 45, 52 or 58** |
| --- | --- | --- | --- |
| Fraction of persistent penile HPV infections* | | | |
| Male | .0760,  (.0751,.1246) | .1106,  (.0940,.1260) | .0551,  (.0406,.1910) |
| Female | .1325,  (.0759,.1526) | .1321,  (.0866,.1464) | .0983,  (.0457,.2181) |
| Clearance rate of penile HPV infections** | | |  |
| Male | 0.520000 | 0.630000 | 0.630000 |
| Female | 0.520000 | 0.630000 | 0.630000 |
| Fraction of people seroconvert following a penile HPV infection* | | |  |
| Male | .0808,  (.0766,.0982) | .0826,  (.0732,.0874) | .0696,  (.0584,.1016) |
| Female | .5984,  (.4868,.6468) | .5702,  (.5603,.6269) | .6496,  (.5164, .7259) |
| Degree of protection against penile HPV infections provided by natural immunity following seroconversion* | | |  |
| Male | .1892,  (.0997, 0.2179) | .1797,  (.1166,.1819) | .1927,  (.0460, .3300) |
| Female | .3559, (.3119,.4118) | .3722,  (.3129, .3723) | .2226,  (.2225, .4216) |
| *From Model calibration  ** From Model calibration for the US | | | |

# Treatment Patterns

The female population receiving hysterectomy over the course of 1 year was estimated from the Survey of Medical Care Activities in Public Health Insurance[40]. These are presented in Table 19.

Table 19: Women receiving hysterectomy over the course of 1 year

| **Age group (years)** | **Value Females (percent)** |
| --- | --- |
| <1 | 0.000% |
| 1-8 | 0.000% |
| 9-10 | 0.000% |
| 11-12 | 0.000% |
| 13-14 | 0.000% |
| 15-17 | 0.000% |
| 18 | 0.000% |
| 19 | 0.000% |
| 20-24 | 0.000% |
| 25-26 | 0.002% |
| 27-29 | 0.002% |
| 30-34 | 0.013% |
| 35-39 | 0.063% |
| 40-44 | 0.261% |
| 45-49 | 0.405% |
| 50-54 | 0.194% |
| 55-59 | 0.060% |
| 60-64 | 0.045% |
| 65-69 | 0.036% |
| 70-74 | 0.029% |
| 75-79 | 0.022% |
| 80-84 | 0.016% |
| >85 | 0.005% |

The parameters related to the percent of precancerous lesions (Cervical Intraepithelial Neoplasia (CIN), Vaginal Intraepithelial Neoplasia (VaIN) and Vulvar Intraepithelial Neoplasia (VIN)) as well as Carcinoma In Situ (CIS) which are treated were estimated through previous model calibrations. This also applies to the parameters related to the percentage of females with cancer recognizing their symptoms and seeking treatment. Table 19 summarizes the values of the parameter related to the percent of precancerous lesions (CIN and VaIN) as well as CIS which are treated by stage.

Table 20: Treatment patterns of all stages of diseases

| **Cervical cancer stage** | **Percent seeking treatment** | **Vaginal/Vulvar cancer stage** | **Percent seeking treatment** | **Anal cancer stage** | **Value  Males (percent)** | **Value  Females (percent)** |
| --- | --- | --- | --- | --- | --- | --- |
| Local disease | 4 | Local disease | 6.5 | Local disease | 3 | 3 |
| Regional disease | 18 | Regional disease | 95 | Regional disease | 9 | 9 |
| Distant disease | 95 | Distant disease | 95 | Distant disease | 95 | 95 |

| **Penile cancer stage** | **Value  Males (percent)** | **Head & Neck cancer stage** | **Value  Males (percent)** | **Value  Females (percent)** |
| --- | --- | --- | --- | --- |
| Local disease | 1.3 | Local disease | 25 | 25 |
| Regional disease | 95 | Regional disease | 65 | 60 |
| Distant disease | 95 | Distant disease | 95 | 95 |

| **Cervical disease stage** | **Percent treated after detection** | **Vaginal Disease Stage** | **Percent treated after detection** |
| --- | --- | --- | --- |
| CIN 1 | 50 | VaIN 1 | 30 |
| CIN 2 | 100 | VaIN 2/3 | 100 |
| CIN 3 | 100 | CIS | 100 |
| CIS | 100 |  |  |

# Cancer Mortality

The model requires HPV-related cancers associated mortality rates stratified by age and stage (local, regional, distant). Cervical cancer related mortality was estimated from Japan-specific 5-year survival data[41]. However, since the model contains a separate compartment for cancer survivors, we recognize that any rate computed from survivor data will span both the cancer compartment and the survivor compartment. Since local cervical cancer mortality data was available for calibration. We took a basic estimate of age-specific death rates:

$$deathrate(i)=\ln\left( survival(i) \right)/5$$

And scaled them to best match the mortality data. This was done for HPV-16 related cancers, and the deaths rates were imputed to the other types, since there is no evidence to suggest that cervical cancer severity is based on HPV type.

Mortality data for the other cancers was computed from UK survival data (from EUROCARE 5[42]), since no local data on mortality was available.

Table 21: Annual cancer-associated mortality by site, age and stage

| **Cancer type** | **Age group (years)** | **Mortality rate** | | |
| --- | --- | --- | --- | --- |
|  |  | **Local Cancer** | **Regional Cancer** | **Distant Cancer** |
| Cervical cancer | 0-14 | 0.000 | 0.000 | 0.000 |
|  | 15-29 | 0.002 | 0.025 | 0.095 |
|  | 30-54 | 0.003 | 0.032 | 0.095 |
|  | 55-64 | 0.006 | 0.029 | 0.100 |
|  | 65+ | 0.011 | 0.037 | 0.117 |
| Vaginal cancer | 0-14 | 0 | 0 | 0 |
|  | 15-44 | 0.021 | 0.036 | 0.064 |
|  | 45-54 | 0.034 | 0.058 | 0.103 |
|  | 55-64 | 0.053 | 0.091 | 0.163 |
|  | 65-74 | 0.072 | 0.124 | 0.220 |
|  | >75 | 0.125 | 0.216 | 0.384 |
| Vulvar cancer | 0-14 | 0 | 0 | 0 |
|  | 15-44 | 0.017 | 0.036 | 0.078 |
|  | 45-54 | 0.027 | 0.058 | 0.125 |
|  | 55-64 | 0.042 | 0.091 | 0.198 |
|  | 65-74 | 0.057 | 0.124 | 0.267 |
|  | >75 | 0.099 | 0.216 | 0.466 |
| Anal cancer (Females) | 0-14 | 0 | 0 | 0 |
|  | 15-54 | 0.038 | 0.084 | 0.144 |
|  | 55-64 | 0.041 | 0.092 | 0.157 |
|  | 65-74 | 0.056 | 0.124 | 0.213 |
|  | >75 | 0.101 | 0.224 | 0.386 |
| Anal cancer (Males) | 0-14 | 0 | 0 | 0 |
|  | 15-44 | 0.043 | 0.096 | 0.165 |
|  | 45-54 | 0.048 | 0.106 | 0.183 |
|  | 55-64 | 0.052 | 0.115 | 0.198 |
|  | 65-74 | 0.065 | 0.144 | 0.248 |
|  | >75 | 0.107 | 0.237 | 0.407 |
| Penile cancer | 0-14 | 0 | 0 | 0 |
|  | 15-44 | 0.011 | 0.052 | 0.115 |
|  | 45-54 | 0.010 | 0.049 | 0.107 |
|  | 55-64 | 0.013 | 0.064 | 0.140 |
|  | 65-74 | 0.020 | 0.093 | 0.205 |
|  | >75 | 0.042 | 0.199 | 0.438 |
| Head & neck cancer (Females) | 0-14 | 0 | 0 | 0 |
|  | 15-44 | 0.040 | 0.058 | 0.072 |
|  | 45-54 | 0.063 | 0.091 | 0.114 |
|  | 55-64 | 0.083 | 0.120 | 0.149 |
|  | 65-74 | 0.099 | 0.143 | 0.179 |
|  | >75 | 0.165 | 0.239 | 0.298 |
| Head & neck cancer (Males) | 0-14 | 0 | 0 | 0 |
|  | 15-44 | 0.056 | 0.081 | 0.101 |
|  | 45-54 | 0.078 | 0.114 | 0.142 |
|  | 55-64 | 0.104 | 0.151 | 0.188 |
|  | 65-74 | 0.141 | 0.204 | 0.255 |
|  | >75 | 0.193 | 0.279 | 0.349 |

# Vaccine Properties

The prophylactic efficacy of the vaccine or vaccine degree of protection is based on clinical trial data. [30, 43-47] The duration of protection against HPV genotypes contained in the vaccines is assumed to be lifelong. No cross-protection effect was considered in this analysis.

## Efficacy against infection vs efficacy against disease

The model considers different efficacy values as the property of the model includes a degree of protection against infection and a degree of protection against disease given a breakthrough infection. In the model it is further assumed that these "breakthrough" infections are transmissible.

## Efficacy against non-cervical cancers

The efficacy against anal, head and neck, penile and RRP diseases is conferred through protection against infection only.

## Efficacy for partial vaccine series completion

Results from a recent systematic review (Markowitz, et al.[48]) were used to estimate the efficacy of partial series completion. Series completion for ages under 15 years is two doses and the efficacy estimates are presented in Table 24. The efficacy for one dose is assumed to be 65% of the series completion efficacy.

Vaccines should have a very similar clinical effect between the US and UK, and thus the following variables are the same for all countries:

- The existence of herd immunity
- The relative effectiveness of the vaccine if fewer than the full regimen of three doses are received
- Duration of protection
- Relative risk of people with breakthrough genital HPV infections transmitting the infection to their partners
- Efficacy of the vaccine against HPV infection
- Degree of protection of the vaccine against HPV infections becoming persistent

Vaccine efficacy assumptions are reported in Table 24 for HPV-related cancers and in Table 25 for HPV-related genital warts.

Table 22: Vaccine efficacy assumptions (international) for HPV-related cancers

| **Vaccine assumptions** | **HPV 16** | **HPV 18** | **HPV 31, 33, 45, 52 and 58** |
| --- | --- | --- | --- |
|  |  |  |  |
| Cervical cancer | | | |
| Male* | 0.411 | 0.411 | 0.411 |
| Female | 0.76 | 0.76 | 0.76 |
| Degree of protection of the vaccine against cervical HPV infections becoming persistent | 0.988 | 0.988 | 0.988 |
| Degree of protection of the vaccine against HPV-related CIN | 0.97 | 0.97 | 0.97 |
| Vaginal and vulvar cancers | | | |
| Vaccine efficacy for preventing vaginal/vulvar HPV infections: |  |  |  |
| Male* | 0.411 | 0.621 | 0.621 |
| Female | 0.76 | 0.963 | 0.963 |
| Degree of protection of the vaccine against vaginal/vulvar HPV infections becoming persistent | 0.988 | 0.984 | 0.984 |
| Degree of protection of the vaccine against HPV-related /VaIN/VIN | 1 | 1 | 1 |
| Anal cancers | | | |
| Vaccine efficacy for preventing anal HPV16/18 infections |  |  |  |
| Male | 0.762 | 1 | 0.762 |
| Female | 0.762 | 1 | 0.762 |
| Degree of protection of the vaccine against anal HPV infections becoming persistent |  |  |  |
| Male | 0.938 | 0.999 | 0.938 |
| Female | 0.938 | 0.999 | 0.938 |
| Degree of protection of the vaccine against HPV -related AIN | 0.655 | 1 | 0.655 |
| H&N cancers | | | |
| Vaccine efficacy for preventing H&N infections |  |  |  |
| Male | 0.411 | 0.621 | 0.621 |
| Female | 0.760 | 0.963 | 0.963 |
| Degree of protection of the vaccine against H&N infections becoming persistent |  |  |  |
| Male | 0.787 | 0.96 | 0.96 |
| Female | 0.988 | 0.984 | 0.984 |
| Degree of protection of the vaccine against HPV-related H&N neoplasia | 0 | 0 | 0 |
| Penile cancer | | | |
| Vaccine efficacy for preventing Penile HPV infections |  |  |  |
| Male | 0.411 | 0.621 | 0.621 |
| Female | 0.760 | 0.963 | 0.963 |
| Degree of protection of the vaccine against penile HPV16/18 infections becoming persistent |  |  |  |
| Degree of protection of the vaccine against HPV-related PIN | 0.787 | 0.960 | 0.960 |

*Preventing male genital infections through male vaccination is assumed to prevent transmission of genital infections to females.

**Preventing female genital infections through vaccination is assumed to prevent transmission of genital infections to males. CIN: *Cervical intraepithelial neoplasia, VaIN: vaginal intraepithelial neoplasia, VIN: vulval intraepithelial neoplasia, AIN: anal intraepithelial neoplasia, PIN: penile intraepithelial neoplasia*

Giuliano et al[45] for males; Garland et al.[49] and Palefsky et al.[47] for females.

Table 23: Vaccine efficacy assumption (international) for genital warts

| **Vaccine assumptions** | **HPV 6** | **HPV 11** |
| --- | --- | --- |
| Vaccine efficacy against HPV 6/11 infection | | |
| Females | 0.761 | 0.761 |
| Males | 0.49 | 0.57 |
| Degree of protection of the vaccine against HPV 6/11-related genital warts | | |
| Females | 0.989 | 1 |
| Males | 0.843 | 0.909 |
| Degree of protection of the vaccine against HPV 6/11-related CIN 1 | 1 | 1 |

Source: Giuliano et al[45] for males; Garland et al.[49] and Palefsky et al.[47] for females.

# Vaccination Strategy

While Japan has had some history of HPV vaccination, we consider the timeframe of their broad usage to be sufficiently short (2010 to June 2013)[50] so as to not have meaningful impact on the resumption of a vaccine program. Thus, in our scenarios, we assumed that no historical vaccination has occurred.

Vaccination strategies are implemented in the model with an age-specific uptake, in which a set percentage of individuals are vaccinated as they age into the next compartment. All vaccination strategies included a ramp up period of five years to the target vaccination rate in the primary cohort: 60% →80%→90%→95%→100%, expressed as percentage of the target vaccination coverage rate (VCR). For the catchup cohorts, a similar ramp up was used, but only over four years: 60% →80%→90%→100%. In baseline scenarios, the primary cohort is 12-year-old girls, and girls aged 13-16 “catch up” for four years and cease. While it is understood that over the long term there will be vaccines given out in these age groups, there is no existing data to suggest how compliance would be distributed across these other age groups.

The vaccine is given in multiple doses spread over a certain period. However, for ease of implementation we assume that all doses are given in a single event. Given that a large proportion of girls being vaccinated are inoculated before the onset of sexual activity, such an implementation will have no impact on results.

Adherence to follow up doses is modeled by assuming that proportions of individuals only get 1,2, or 3 doses during their vaccination event. In the baseline scenario complete adherence was modeled (everyone who gets vaccinated gets three doses), and the proportions were varied for sensitivity analyses.


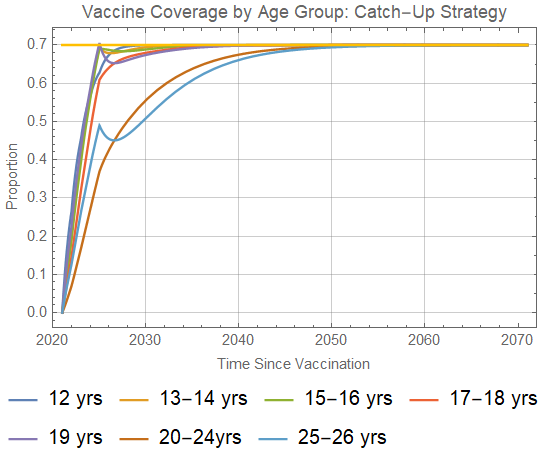


Figure 2. Vaccine coverage

# Costs

All costs were estimates from the 2020 National health Insurance Fee Schedule.

## Cost of vaccine administration

The administration cost per dose is ¥3,718.

## Cost of vaccination

In baseline scenarios, the cost of the 9-valent HPV vaccine was ¥20,000 per dose.

## Cost per episode of care

All costs per episode of care are reported in Table 24.

Table 24: Costs of diagnosing and treating diseases caused by HPV infection

| **Parameter** | **Cost (¥)** |
| --- | --- |
| **- CIN 1** | 76,000 |
| **- CIN 2** | 276,400 |
| **- CIN 3, CIS** | 276,400 |
| **- Cervical cancer, local disease*** | 2,181,900 |
| **- Cervical cancer, regional disease*** | 2,914,300 |
| **- Cervical cancer, distant disease*** | 4,020,800 |
| **- VaIN 1** | 76,000.0 |
| **- VaIN 2** | 533,700.0 |
| **- VaIN 3, CIS** | 533,700.0 |
| **- Vaginal cancer, local disease*** | 991,400.0 |
| **- Vaginal cancer, regional disease*** | 1,823,400.0 |
| **- Vaginal cancer, distant disease*** | 1,977,400.0 |
| **- Vulvar cancer, local disease*** | 818,500.0 |
| **- Vulvar cancer, regional disease*** | 1,694,800.0 |
| **- Vulvar cancer, distant disease*** | 1,847,200.0 |
| **- Penile cancer, local disease*** | 647,000.0 |
| **- Penile cancer, regional disease*** | 1,165,000.0 |
| **- Penile cancer, distant disease*** | 1,249,600.0 |
| **- Anal cancer, local disease*** | 1,183,000.0 |
| **- Anal cancer, regional disease*** | 1,809,200.0 |
| **- Anal cancer, distant disease*** | 2,659,300.0 |
| **- Head & Neck cancer, local disease*** | 1,169,110.0 |
| **- Head & Neck cancer, regional disease*** | 3,695,570.0 |
| **- Head & Neck cancer, distant disease*** | 5,794,276.0 |
| **- Genital warts** | 11,356.0 |
| **- Recurrent respiratory papillomatosis** | 1,144,296.0 |

* Disease stages can be related to the traditional Tumour-Node-Metastasis (TNM) classification system as followed: - "Local disease" corresponds to stages I and II TNM classification, i.e., localized primary tumour; "Regional disease" corresponds to stage III TNM classification system, i.e., metastasis to regional lymph nodes; "Distant disease" corresponds to stage IV TNM classification system, i.e., distant metastatic disease.

## Cost of screening and diagnostic tests

The costs of screening and diagnostic tests were also gleaned from the 2020 National health Insurance Fee Schedule[51]. The different costs are reported in Table 25.

Table 25: Screening and diagnostic tests for cervical and vaginal cancers

| **Parameter** | **Value (¥)** |
| --- | --- |
| Screening (cytology) and office visit | 3730.0 |
| Colposcopy | 5,100.0 |
| Biopsy | 7,000.0 |

# Health-Related Quality of Life

A discount rate for both costs and benefits of 2% was used and was varied for sensitivity analyses.

Age-specific health utility values in the healthy population were based on a UK-specific catalogue of EQ-5D scores from Sullivan et al.[52] shown in tables below.

Table 26: Age-specific utilities in healthy population

| **Age group (year)** | **Male Utility** | **Female Utility** |
| --- | --- | --- |
| 1-17 | 0.947 | 0.946 |
| 18-34 | 0.957 | 0.933 |
| 35-44 | 0.948 | 0.917 |
| 45-54 | 0.936 | 0.921 |
| 55-64 | 0.896 | 0.881 |
| 65+ | 0.853 | 0.808 |

*Unit: number between 0 and 1*

Table 27: Utilities in population with HPV-related diseases

| **Condition** | **Value** |
| --- | --- |
| CIN1, VaIN1, VIN1 | 0.91 |
| CIN2+, CIS, VaIN2+, VIN2+ | 0.87 |
| Cervical/Vaginal/Vulvar/Anal/H&N/Penile Cancer, Local | 0.76 |
| Cervical/Vaginal/Vulvar/Anal/H&N/Penile Cancer, Regional | 0.67 |
| Cervical/Vaginal/Vulvar/Anal/H&N/Penile Cancer, Distant | 0.48 |
| Cervical/Vaginal/Vulvar/Anal/H&N/Penile Cancer, Survivor | 0.76 |
| Genital Warts | 0.91 |
| RRP | 0.79 |

Sources: * calculated based on Sullivan et al. (2011)[52], Dominiak-Felden et al. (2013)[53] and Chadha et al. (2010)[53]

*Unit: number between 0 and 1*

# Calibration Process

## Data sources

The model was calibrated utilizing CIN prevalence, cancer incidence data, HPV type attribution, genital warts incidence, and RRP incidence. Mortality data for cervical cancer was also included in the calibration targets. Cervical cancer data (incidence and mortality) was obtained from the National Cancer Center in Japan[13], and we used the average incidence from 2011 to 2015. Incidence data for other cancers was obtained from GLOBOCAN[11]. Where available, the National level data was used. When only prefecture specific data was available, we used Aichi Prefecture, assuming that it was representative for all of Japan. Collected data is given in Table 33-.

Table 28: Cancer Incidence (cases per 100,000) Data for Females

| **Female** | **Cervical** | **Vaginal** | **Vulvar** | **Anal** | **Oropharynx** |
| --- | --- | --- | --- | --- | --- |
| 15-19 | 0.04 | 0 | 0 | 0 | 0.00 |
| 20-24 | 1.53 | 0 | 0 | 0 | 0.00 |
| 25-29 | 10.22 | 0 | 0 | 0 | 0.00 |
| 30-34 | 21.64 | 0.1 | 0 | 0 | 0.00 |
| 35-39 | 27.32 | 0 | 0.1 | 0.1 | 0.00 |
| 40-44 | 31.54 | 0.3 | -- | 0.1 | 0.5 |
| 45-49 | 27.47 | 0.4 | -- | 0.8 | 0.5 |
| 50-54 | 23.16 | 0.5 | -- | 0.3 | 1.1 |
| 55-59 | 20.24 | 0.4 | 0.8 | 1 | 1.5 |
| 60-64 | 20.17 | 0.5 | 0.7 | 0.9 | 1.7 |
| 65-69 | 18.12 | 0.4 | 1.7 | 1 | 1.7 |
| 70-74 | 17.11 | 1.1 | 2.4 | 1.4 | 1.7 |
| 75-79 | 17.44 | 1 | 2.6 | 2.3 | 2.1 |
| 80-84 | 16.97 | 1.3 | 4.3 | 2 | 2.1 |
| >85 | 18.2 | 1.2 | 7.7 | 2.4 | 2.1 |

Table 29: Cancer Incidence (cases per 100,000) Data for Males

| **Male** | **Penile** | **Anal** | **Oropharynx** |
| --- | --- | --- | --- |
| 15-19 | 0 | 0 | 0 |
| 20-24 | 0 | 0 | 0 |
| 25-29 | 0 | 0 | 0 |
| 30-34 | 0 | 0.1 | 0 |
| 35-39 | 0.1 | 0 | 0 |
| 40-44 | 0.1 | 0 | 0.3 |
| 45-49 | 0.4 | 0.5 | 0.9 |
| 50-54 | 0.2 | 0.3 | 2.3 |
| 55-59 | 0.6 | 1 | 4.4 |
| 60-64 | 0.8 | 1.1 | 7.9 |
| 65-69 | 1.3 | 2.7 | 11.2 |
| 70-74 | 2.7 | 1.2 | 12 |
| 75-79 | 1.7 | 1.5 | 10 |
| 80-84 | 3.5 | 3.2 | 10 |
| >85 | 3.5 | 5.2 | 10 |

Table 30 Cervical Cancer Mortality Data

| **Female** | **Deaths per 100,000** |
| --- | --- |
| 15-19 | 0 |
| 20-24 | 0.06 |
| 25-29 | 0.47 |
| 30-34 | 1.51 |
| 35-39 | 2.67 |
| 40-44 | 3.67 |
| 45-49 | 5.40 |
| 50-54 | 6.45 |
| 55-59 | 5.75 |
| 60-64 | 5.61 |
| 65-69 | 5.95 |
| 70-74 | 6.23 |
| 75-79 | 7.26 |
| 80-84 | 8.50 |
| >85 | 11.92 |

HPV type attribution was obtained from several studies depending on the disease. For cervical cancers and neoplasia, Japan-specific attribution was obtained from Sakamoto et al[17] . Other cancer attributions were obtained from a series of meta-analyses[54-58], focusing on Asia-specific attributions where available. Attribution for genital warts data was obtained from a separate study[59]. For simplicity, it was assumed that all cases of JORRP are caused by HPV6 since no specific data was available for JORRP attribution. JORRP incidence data from the US (0.73 per 100,000 children under the age of 18) was used.[60]

Table 31: HPV Type Attributions for Male Disease

| **Male** | **HPV 6** | **HPV 11** | **HPV 16** | **HPV 18** | **HPV type 31, 33, 45, 52 or 58** |
| --- | --- | --- | --- | --- | --- |
| Penile | 0 | 0 | 0.0921 | 0.0206 | 0.0025 |
| Head and Neck | 0 | 0 | 0.2067 | 0.0045 | 0.0022 |
| Anal | 0 | 0 | 0.6147 | 0.0276 | 0.0107 |
| Genital Warts | .7409 | .1591 | 0 | 0 | 0 |

Table 32: HPV Type Attribution for Female Disease

| **Female** | **HPV 6** | **HPV 11** | **HPV 16** | **HPV 18** | **HPV type 31, 33, 45, 52 or 58** |
| --- | --- | --- | --- | --- | --- |
| Cervical | 0 | 0 | 0.4870 | 0.1670 | 0.0458 |
| CIN 1 | 0.0460 | 0.0160 | 0.2040 | 0.0490 | 0.0440 |
| CIN 2+ | 0 | 0 | 0.3960 | 0.057 | 0.0714 |
| Vaginal | 0 | 0 | 0.4218 | 0.0370 | 0.0281 |
| Vulvar | 0 | 0 | 0.1530 | 0.0097 | 0.0058 |
| Anal | 0 | 0 | 0.6147 | 0.0276 | 0.0107 |
| Head and Neck | 0 | 0 | 0.2067 | 0.0045 | 0.0022 |
| Genital Warts | 0.6272 | 0.1347 | 0 | 0 | 0 |

Table 33: Genital Warts Incidence (cases per 100,000) in Japan

| Gender | Incidence |
| --- | --- |
| Male | 73.6 (23.3, 123.8) |
| Female | 49.2 (32.6, 77.6) |

# Calibration Results

The model is calibrated to pre-vaccination data, under the assumption that HPV infections and disease incidence has reached an equilibrium prior to the rollout of the vaccine. The variance for the prevalence was determined through the Central limit theorem, and the computation for the variance of the incidence comes from Boyle and Parkin (1991).[61]

The model was fit using Bayesian History Matching, following the excellent tutorial of Adrianakis et al[62]. For large models, such as ours, calibration is achieved through the construction of Gaussian Process emulators of the model. Such emulators make a statistical estimation of the model outputs using training data from the parameter space. Due to the Bayesian nature of the construction, these estimates have variance estimates of their own. These are combined with the variance of the data to rule out portions of the space that are implausible. That is to say, if the variance of the emulator estimate, and the variance of the data are insufficient to explain the difference between the data and emulator output, then such a point is designated as implausible.

Such a calibration method is done iteratively, and at each “wave” the space of non-implausible points becomes smaller, improving emulator estimates i.e. reducing emulator variance. The algorithm is stopped when there is no significant decrease in the size of the non-implausible space. For each wave, an approximately uniform sample of the non-implausible region must be generated. We adapted a simple genetic algorithm[63] in which the sample of training points used at the beginning of the wave are evolved into a sample of the new non-implausible space as follows:

1. The implausibility is evaluated at each of the N points in the sample. This gives the “fitness” of each point, where smaller implausibility represents higher fitness. If the implausibility was less than 2, it was assigned a fitness of 2, so that every non-implausible point is considered equally “fit”.
2. For each point in the sample a mate is chosen at random, with every other point weighted by fitness.
3. Once a mate is chosen, a child is produced by uniformly sampling a point along the line between the two points. The implausibility is evaluated at this child point. If it is better than both of its parents, then it is added to the next generation. If it is less fit than a parent, the most fit parent is chosen to move on to the next generation, after mutation by a normal random walk.
4. Once this has been done for each point in the current generation, a new generation of N points is produced. The number of non-implausible points is counted, and if less than 90% of the samples are non-implausible, then we return to Step 1 with the new generation.

Such a sampling algorithm relies on accurate emulators and a sufficiently rich initial sample of the parameter space. The number of training points could be increased to improve the density of the sample, and validation points were used at each wave to compare the emulator outputs and the model outputs. “Bad” emulators can be excluded at any wave, so as to prevent the propagation of implausible points. Standard diagnostics[64] were used to assess emulator performance, and to help direct efforts for improvement.

The net result of the above methods is a set of non-implausible points, but these do not form the final parameter set, since it would be inaccurate to assume that all such non-implausible points are equally likely. Hence, precisely as in Adrianakis et al.[62], the non-implausible space was resampled to produce estimates of the posterior distribution. The point with the highest likelihood was assigned as the maximum likelihood estimator (MLE). For our primary baseline scenarios, 100 points were sampled from the posterior to explore variability. For scenarios run for the purpose of sensitivity, we considered only the MLE, since extensive running of the model was computationally prohibitive.

Calibrated outcomes are shown below in Figure 2-Figure 15. In some cases, the low incidence led to the calibration admitting a disease-free state. In the few cases that this occurred, we have used only the MLE for our runs. Variation in parameters would only produce marginal changes in the outcomes, since the incidence of these cancers are so low. For some models, the posterior likelihood was so tightly peaked at the MLE that when weighted resampling on non-implausible points was done, the MLE was sampled for most of the draws. This is seen in the plot as a sparse few trajectories against the data. A denser sampling scheme could produce a diversity of points, but they would still be very close the MLE. As such, the results would be, essentially, the same.


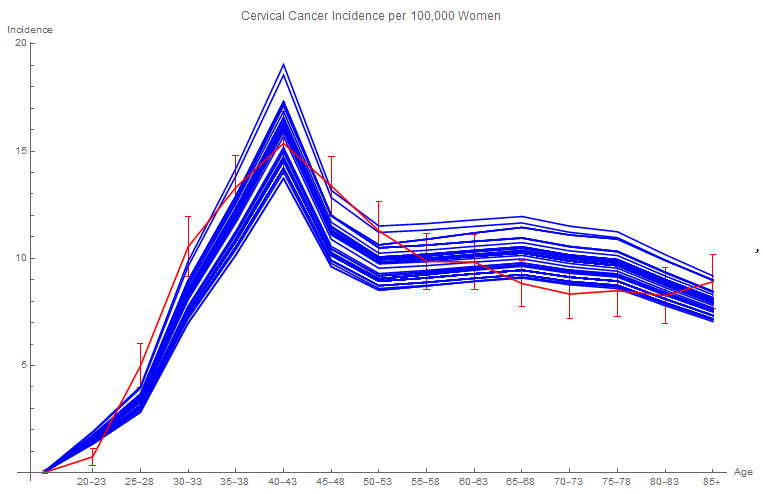

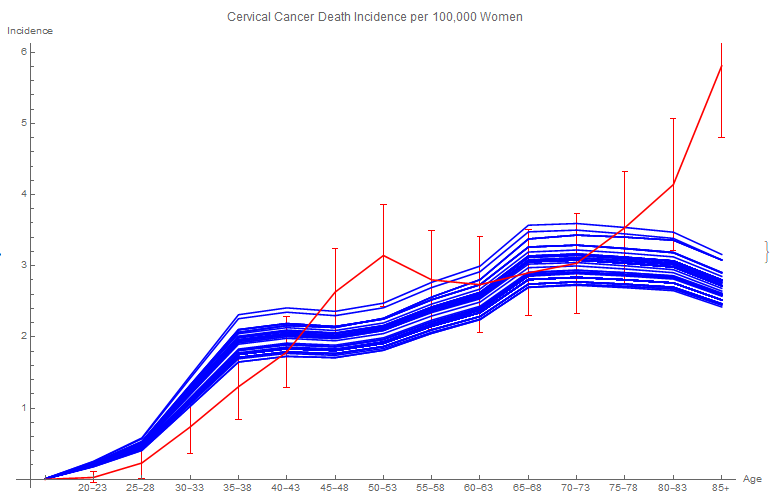

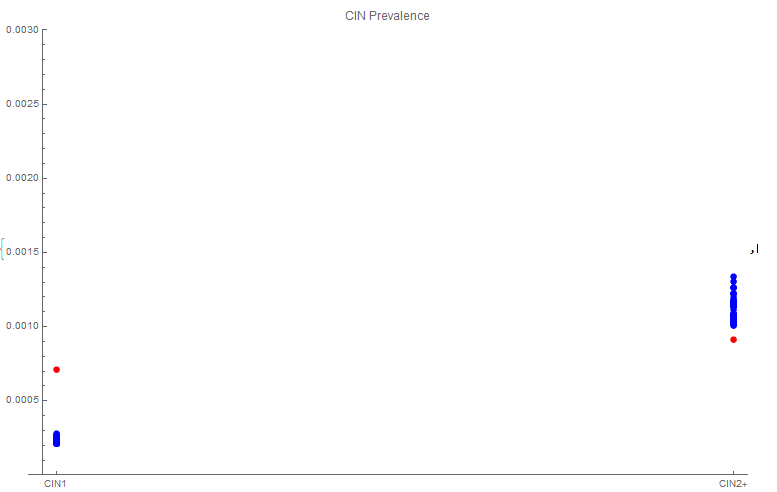


Figure 3 HPV16 related cervical disease, calibrated fits. Red represents the data, blue the output of the model from 100 posterior draws


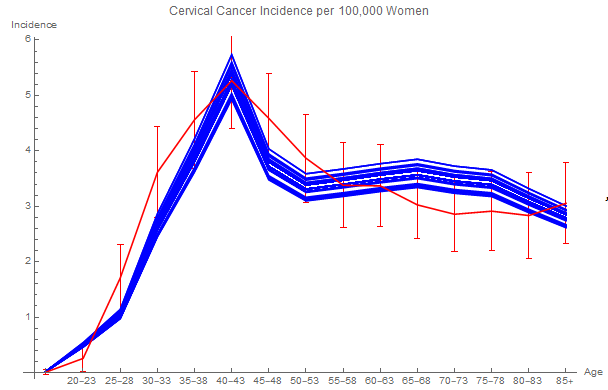

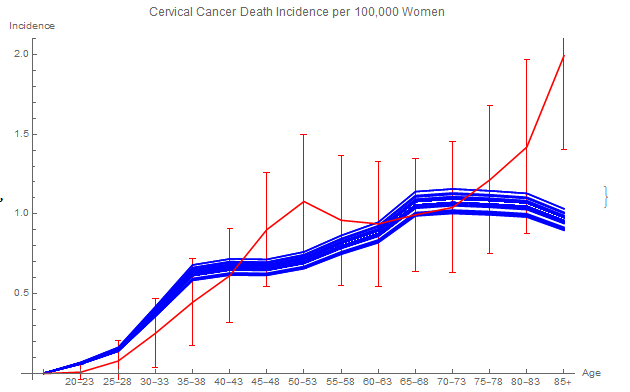

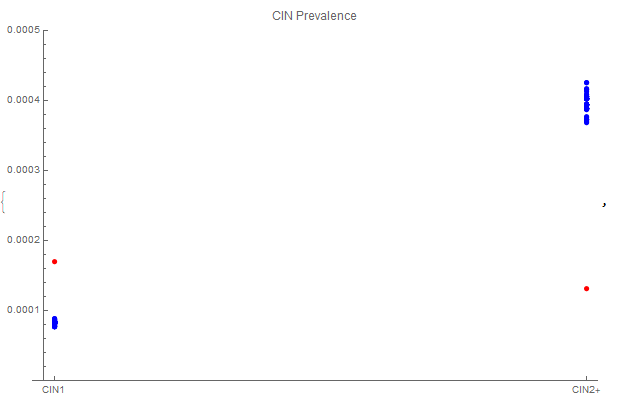


Figure 4 HPV18- related cervical disease, calibrated fits. Red represents the data, blue the output of the model from 100 posterior draws. The dramatic overestimation of CIN2+ prevalence is investigated in sensitivity analyses (Section 11)


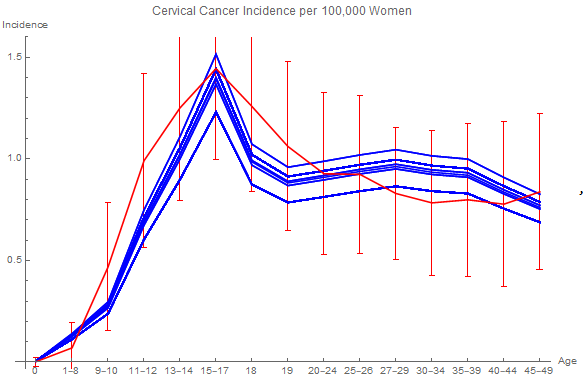

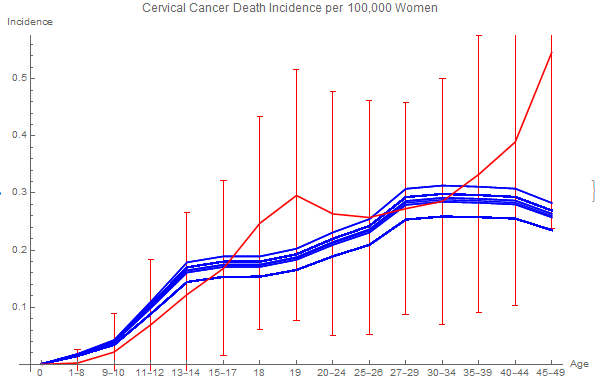

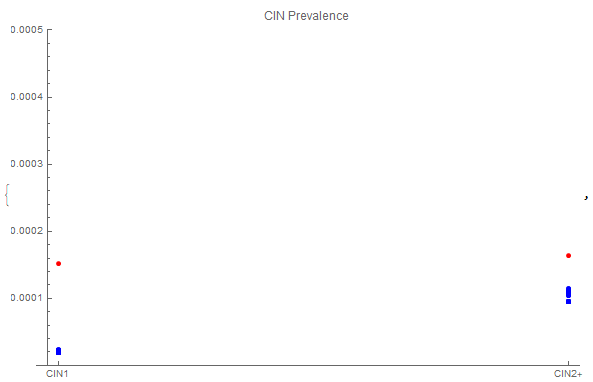


Figure 5 HPV31 related cervical disease, calibrated fits. Red represents the data, blue the output of the model from 100 posterior draws.


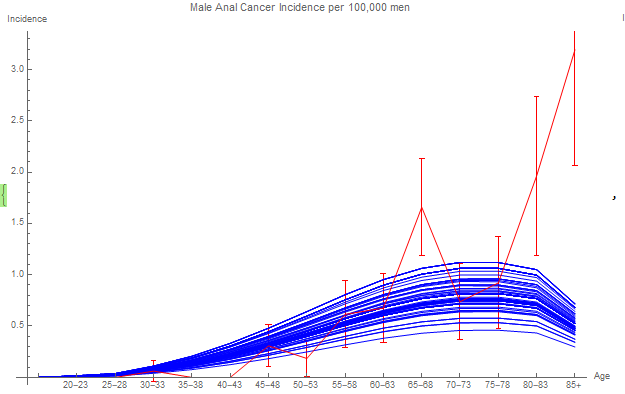

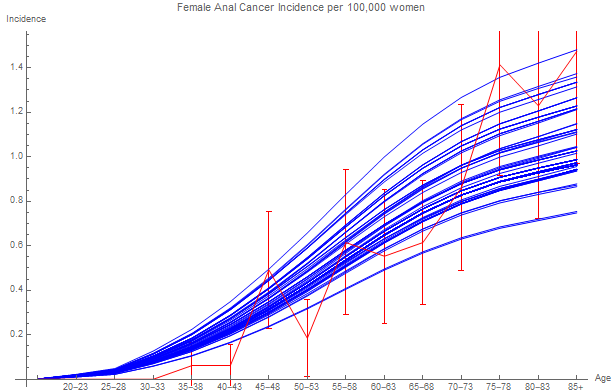


Figure 6 HPV16 related anal cancer for males and females, calibrated fits. Red represents the data, blue the output of the model from 100 posterior draws.


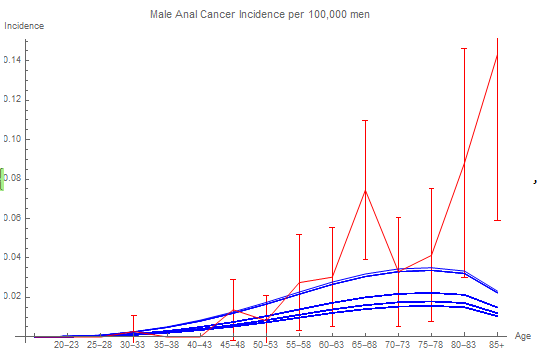

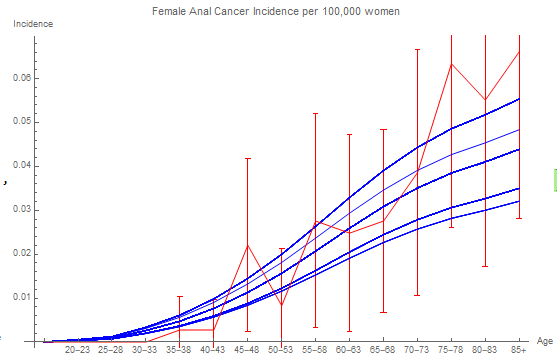


Figure 7 HPV18 related anal cancer for men and women, calibrated fits. Red represents the data, blue the output of the model from 100 posterior draws. Sparsity is due to repeatedly sampling parameter sets. The likelihood here was tightly peaked, so when weighted resampling of parameter sets was done to create an approximate posterior, the MLE was repeatedly sampled most of the time.


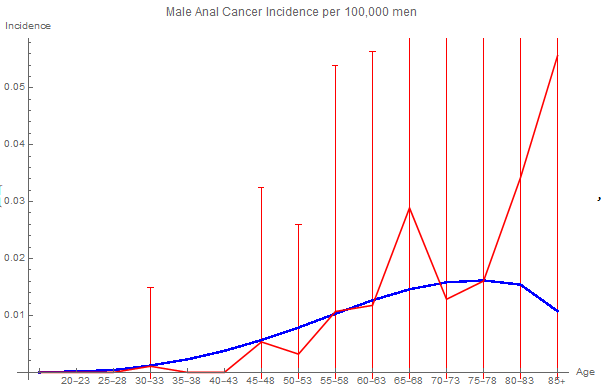

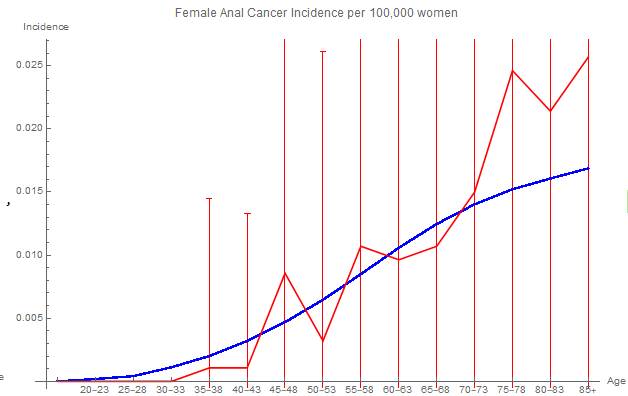


Figure 8 HPV31+ related anal cancer in men and women. Due to the relatively small incidence, the model calibration would admit the disease-free state. In this case we opted to just apply the MLE, which is what is plotted here.


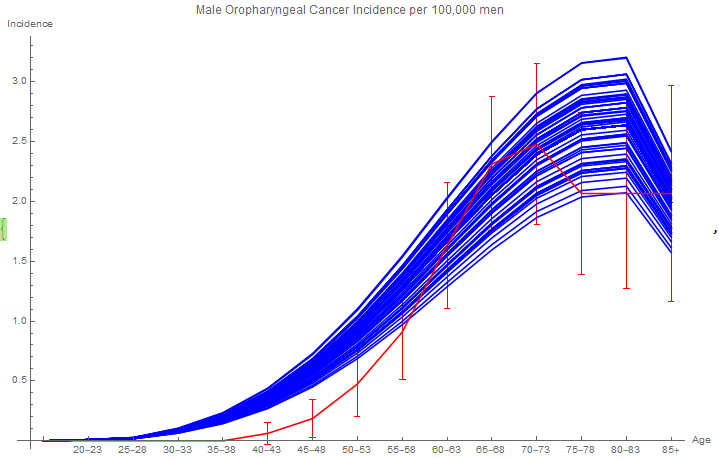

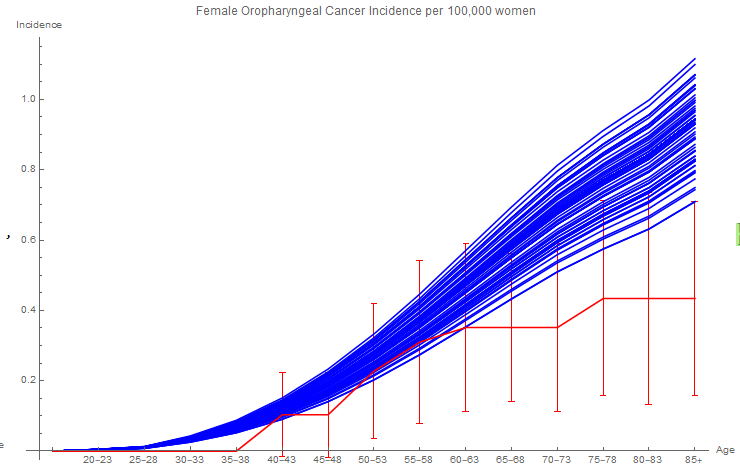


Figure 9 HPV16 related oropharyngeal cancer among men and women, calibrated fits. Red represents the data, blue the output of the model from 100 posterior draws


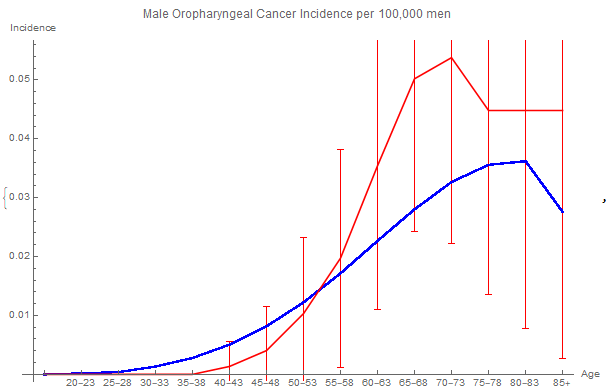

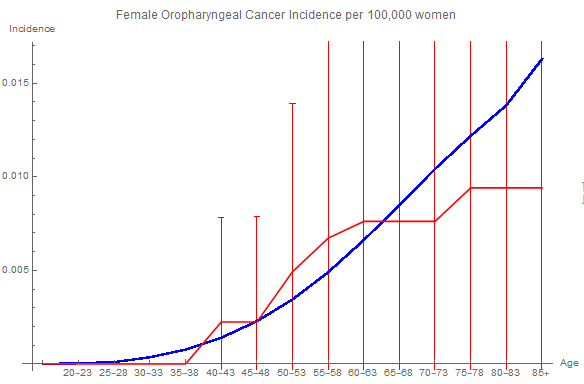


Figure 10 HPV18 related oropharyngeal cancer in males and females, calibrated fit. Due to the relatively small incidence, the model calibration would admit the disease-free state. In this case we opted to just use the MLE, which is what is plotted here.


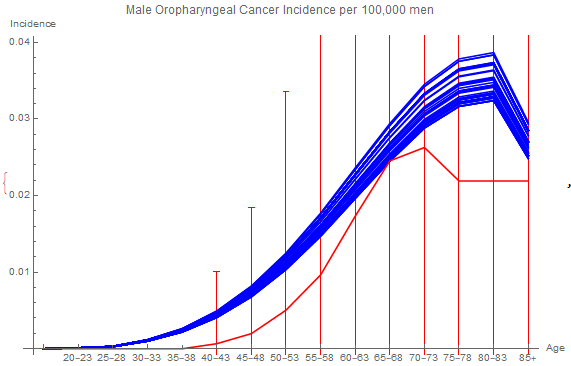

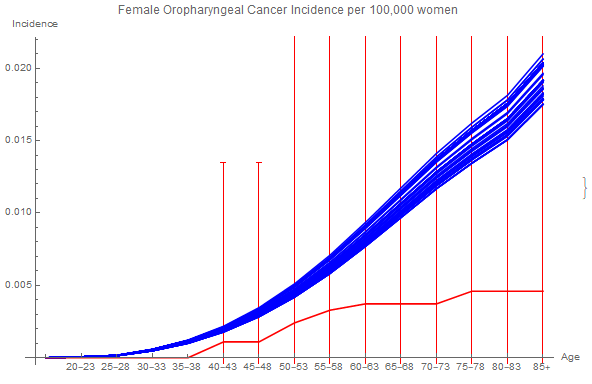


Figure 11 HPV31 related oropharyngeal cancer in males and females, calibrated fits. Red is the data, blue are the model outputs from 100 posterior draws.


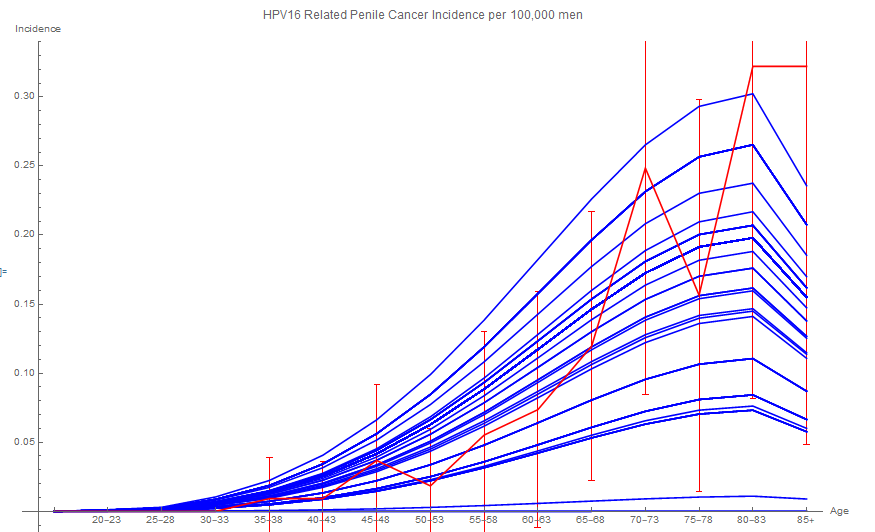

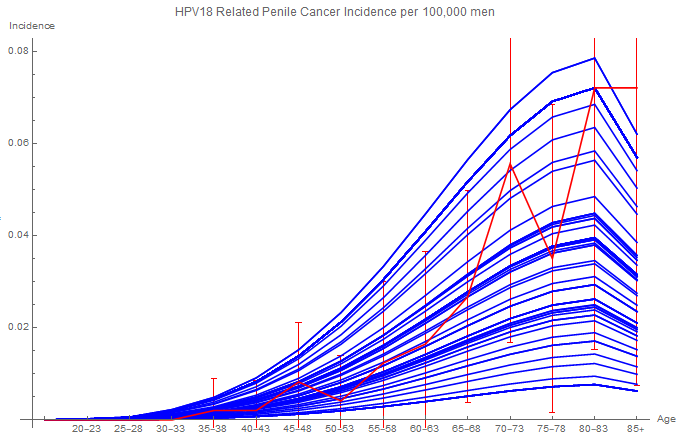

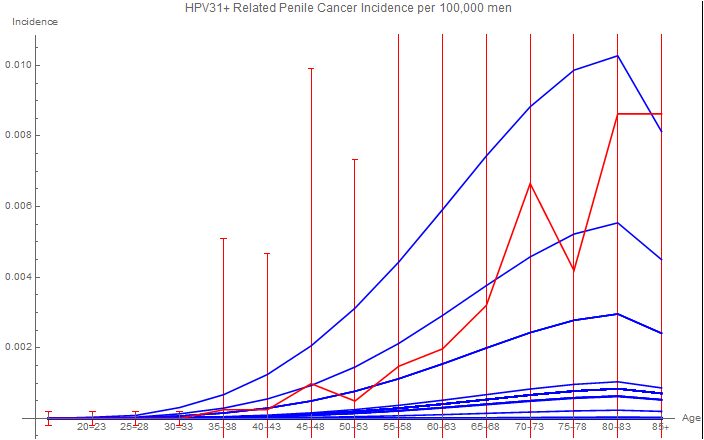


Figure 12 HPV related penile cancer in men. Red is the data, blue are the model outputs from 100 posterior draws.


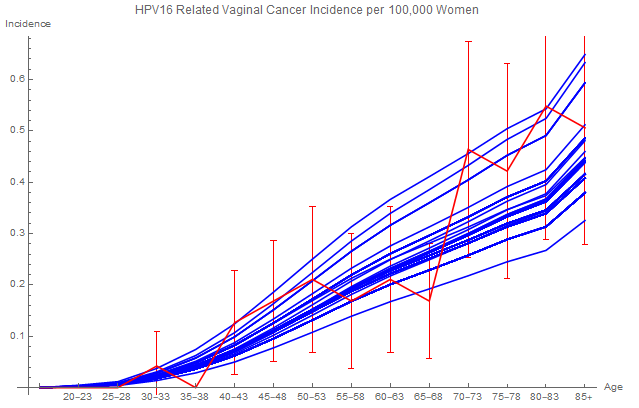

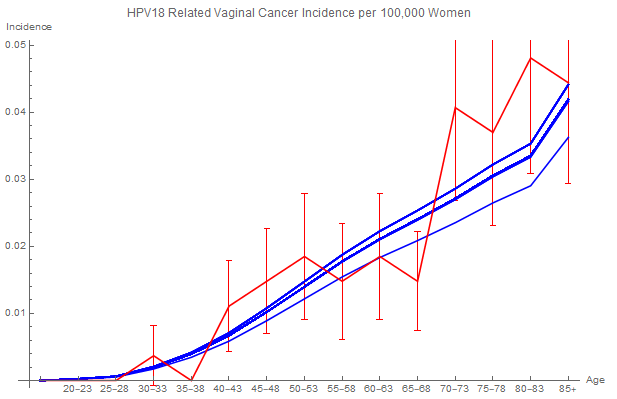

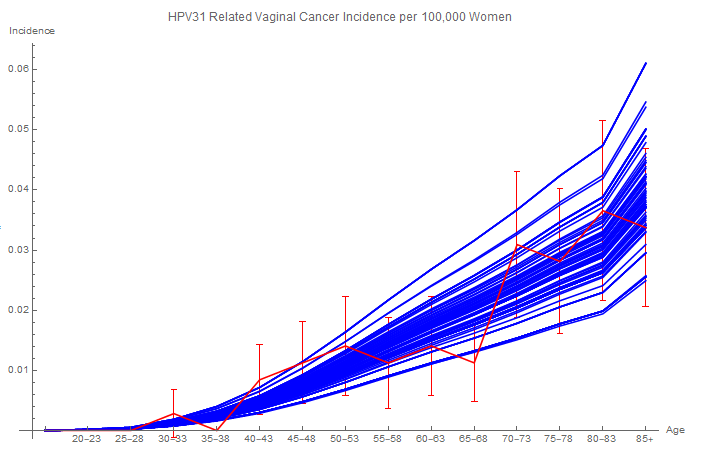


Figure 13 HPV related vaginal cancer. Red is the data, and blue is the output of the model for 100 posterior draws. Sparsity in the middle figure is due to a tightly peaked likelihood, so that weighted resampling predominately drew the MLE.


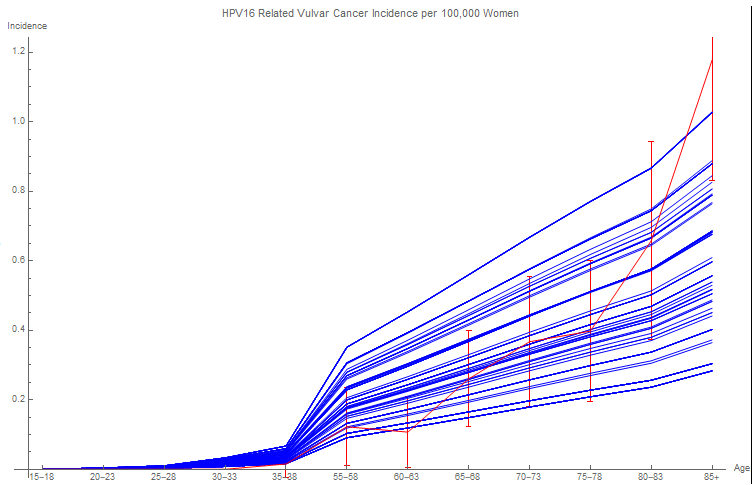


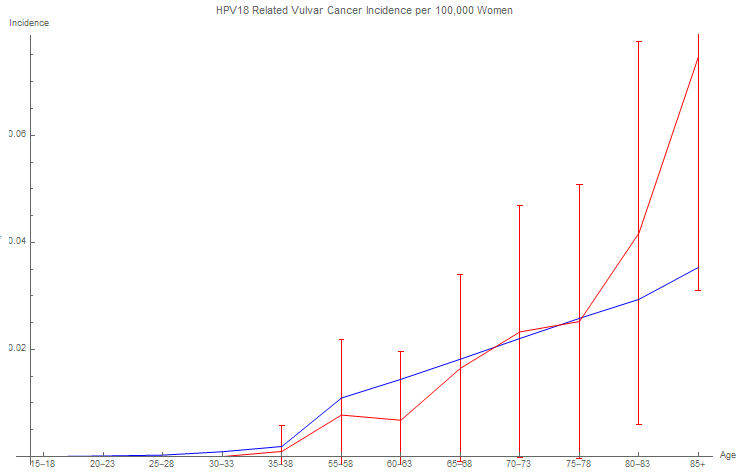

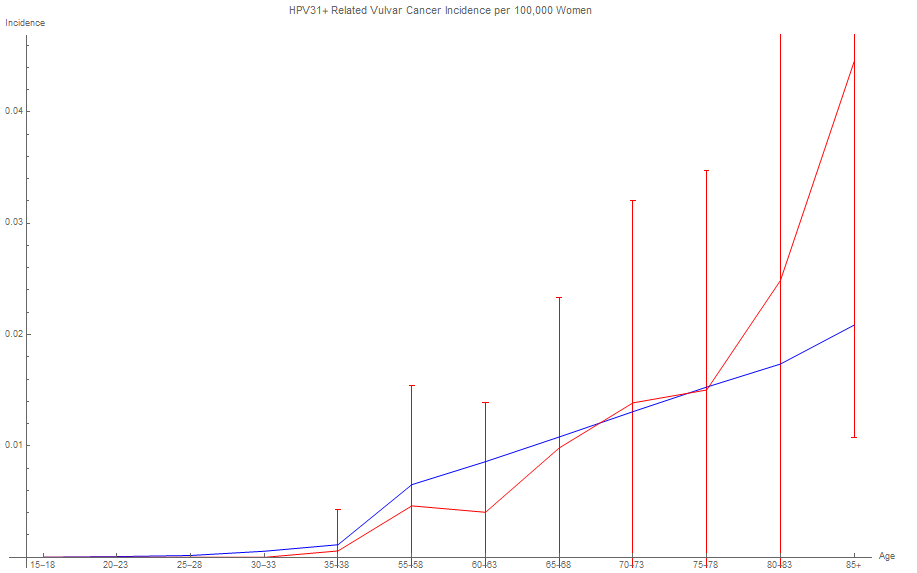


Figure 14 HPV related vulvar cancer in women. Red is the data, and blue is the output of the model for 100 posterior draws. Due to the relatively small incidence, the model calibration would admit the disease-free state for HPV18 and HPV31+. In this case we opted to just use the MLE, which is what is plotted here.


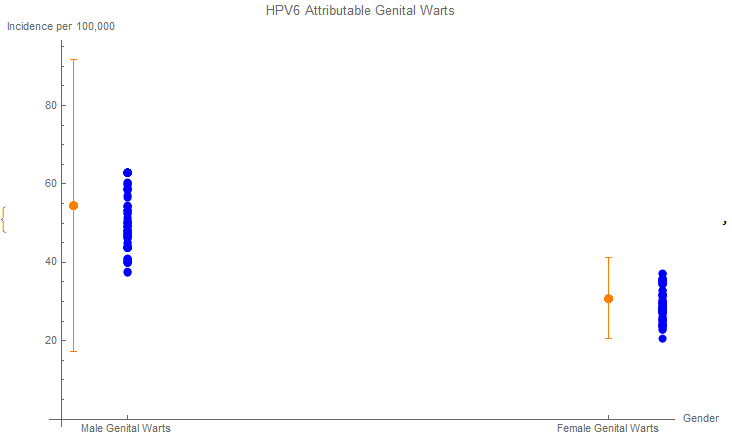


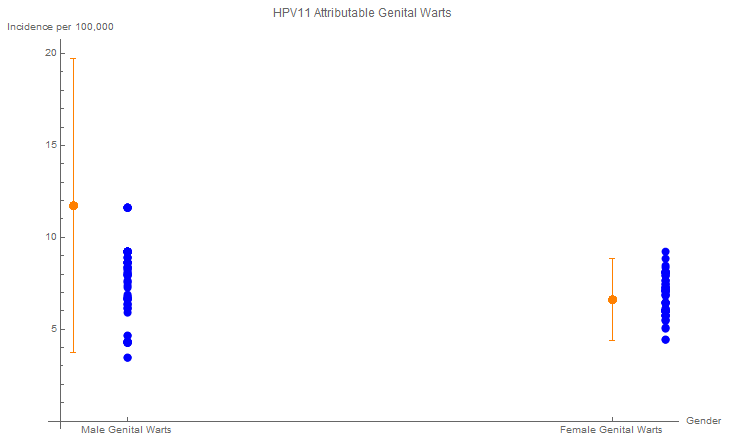


Figure 15 HPV 6 and 11 attributable genital warts. Data is in orange, and the output from the model for 100 posterior draws in in blue.


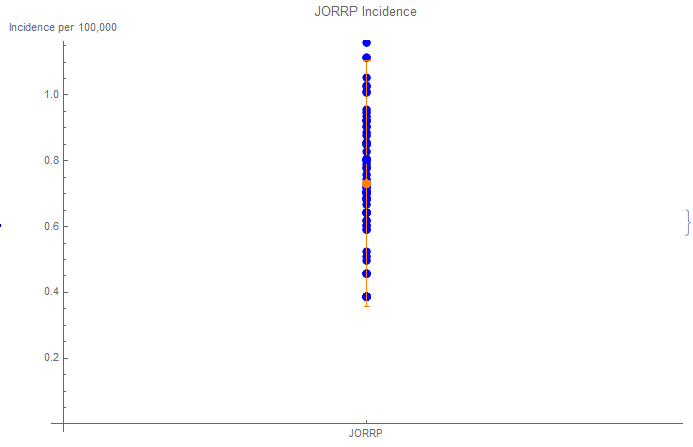


Figure 16 JORRP incidence. Data is in orange, and model output for 100 posterior draws is shown in blue.

# Sensitivity Analyses

A deterministic sensitivity analysis was run on various scenario-related parameters described below. For each variable, we present the per capita Costs and QALYs, and the subsequent ICER. These results are also computed incrementally adding in each disease of interest, to clearly elucidate the contribution of each disease to the ICER.

Before detailing the results of each sensitivity scenario, these analyses are summarized in two tornado diagrams that focus on ICER sensitivity when the included diseases are cervical disease and genital warts, and all diseases of interest. The tornado diagram for the ICERs including only cervical disease and genital warts can be found in Figure 16. The tornado diagram for the ICERs including all diseases are in Figure 17.

Figure 17 ICER sensitivities when only considering cervical disease and genital warts.

Figure 18 ICER sensitivities when all diseases are included.

## Baseline Scenario

For the purpose of sensitivity, the model was run with altered values from the baseline scenario and the change in the ICER was investigated. The baseline scenario consisted of the following:

1. 5 year ramp up to 70% vaccine uptake in the primary cohort (12-year-old girls), and a four year ramp up to 50% uptake in the catch-up cohorts (13-26, depending on the strategy).
2. Adherence to the number of doses (3) is 100% in all cohorts.
3. Vaccine gives lifelong duration of protection.
4. Utilities are as given in the tables above.
5. Disease costs are as given above.
6. Vaccine price is ¥20,000 per dose.
7. Discount rate is 2%.

The results for the baseline scenario are given in Table 34.

Table 34 Costs/QALYs and ICERs for the baseline scenario.

| Cervical Only | | |  |
| --- | --- | --- | --- |
| Strategy | Cost (¥) | QALY | ICER (¥/QALY) |
| No Vaccination | 34650.19655 | 39.71734089 | -- |
| 4VHPV, GIRLS ONLY | 40403.67831 | 39.72160332 | 1,349,812.61 |
| 9VHPV, GIRLS ONLY | 43713.54673 | 39.72325346 | Weakly Dominated |
| 9VHPV, GIRLS ONLY + CATCHUP | 44810.37395 | 39.72420021 | 1,696,912.71 |
| Cervical + GW | | |  |
| Strategy | Cost | QALY | ICER |
| No Vaccination | 34957.90853 | 39.71584041 | -- |
| 4VHPV, GIRLS ONLY | 40526.74054 | 39.72099155 | 1,081,087.30 |
| 9VHPV, GIRLS ONLY | 43836.60896 | 39.72264169 | Weakly Dominated |
| 9VHPV, GIRLS ONLY + CATCHUP | 44909.6928 | 39.72370238 | 1,616,830.37 |
| Cerivcal+GW+6/11CIN | | |  |
| Strategy | Cost | QALY | ICER |
| No Vaccination | 34986.71266 | 39.71582205 | -- |
| 4VHPV, GIRLS ONLY | 40539.07547 | 39.72098361 | 1,075,714.09 |
| 9VHPV, GIRLS ONLY | 43848.94388 | 39.72263375 | Weakly Dominated |
| 9VHPV, GIRLS ONLY + CATCHUP | 44919.7602 | 39.72369588 | 1,615,135.93 |
| Cervical+GW+6/11CIN+RRP | | |  |
| Strategy | Cost | QALY | ICER |
| No Vaccination | 35025.59731 | 39.71567476 | -- |
| 4VHPV, GIRLS ONLY | 40553.55994 | 39.7208781 | 1,062,387.36 |
| 9VHPV, GIRLS ONLY | 43863.42836 | 39.72252824 | Weakly Dominated |
| 9VHPV, GIRLS ONLY + CATCHUP | 44931.23897 | 39.72359651 | 1,610,382.18 |
| All Disease | | |  |
| Strategy | Cost | QALY | ICER |
| No Vaccination | 36455.41521 | 39.71149928 | -- |
| 4VHPV, GIRLS ONLY | 41827.43034 | 39.71700699 | 975,362.74 |
| 9VHPV, GIRLS ONLY | 45126.23263 | 39.7186848 | Weakly Dominated |
| 9VHPV, GIRLS ONLY + CATCHUP | 46159.69993 | 39.71983025 | 1,534,491.90 |

## Removal of CIN2+

The impact of the overestimation of CIN2+ prevalence given in the model needs to be considered. To make sure that such an overestimation is not qualitatively changing the results of the baseline scenario, we ran a scenario where no costs are incurred by treatment of CIN2+, and there is no quality of life decrement associated with having the condition. All other values are held the same as in the baseline scenario. Hence, in this scenario CIN2+ is not contributing anything to the economic analysis. Obviously, such a scenario is not realistic, but we are presenting it here to show that the overestimation in the model is not affecting the qualitative results regarding cost-effectiveness. These results are presented in Table 35. While the ICERs have gone up, they are still well below the ¥5,000,000 per QALY threshold of cost-effectiveness for Japan.

Table 35 Costs, QALYs, and ICERs for the baseline scenario without CIN2+.

| Cervical Only | | |  |
| --- | --- | --- | --- |
| Strategy | Cost (¥) | QALY | ICER (¥/QALY) |
| No Vaccination | 31798.23399 | 39.71867 | -- |
| 4VHPV, GIRLS ONLY | 38458.64485 | 39.72251 | 1,735,318.55 |
| 9VHPV, GIRLS ONLY | 42131.52595 | 39.72399 | WEAKLY DOMINATED |
| 9VHPV, GIRLS ONLY + CATCHUP | 43407.04873 | 39.72485 | 2,111,288.05 |
| Cervical + GW | | |  |
| Strategy | Cost | QALY | ICER |
| No Vaccination | 32086.13171 | 39.71717 | -- |
| 4VHPV, GIRLS ONLY | 38573.2763 | 39.7219 | 1,372,966.89 |
| 9VHPV, GIRLS ONLY | 42246.15741 | 39.72338 | WEAKLY DOMINATED |
| 9VHPV, GIRLS ONLY + CATCHUP | 43499.50151 | 39.72435 | 2,004,597.05 |
| Cerivcal+GW+6/11CIN | | |  |
| Strategy | Cost | QALY | ICER |
| No Vaccination | 32095.12158 | 39.71716 | -- |
| 4VHPV, GIRLS ONLY | 38577.18045 | 39.72189 | 1,369,435.37 |
| 9VHPV, GIRLS ONLY | 42250.06155 | 39.72337 | WEAKLY DOMINATED |
| 9VHPV, GIRLS ONLY + CATCHUP | 43502.70283 | 39.72435 | 2,003,357.04 |
| Cervical+GW+6/11CIN+RRP | | |  |
| Strategy | Cost | QALY | ICER |
| No Vaccination | 32134.00624 | 39.71701 | -- |
| 4VHPV, GIRLS ONLY | 38591.66492 | 39.72178 | 1,352,344.66 |
| 9VHPV, GIRLS ONLY | 42264.54603 | 39.72327 | WEAKLY DOMINATED |
| 9VHPV, GIRLS ONLY + CATCHUP | 43514.1816 | 39.72425 | 1,997,151.87 |
| All Disease | | |  |
| Strategy | Cost | QALY | ICER |
| No Vaccination | 33563.82414 | 39.71283 | -- |
| 4VHPV, GIRLS ONLY | 39865.53532 | 39.71791 | 1,240,611.38 |
| 9VHPV, GIRLS ONLY | 43527.3503 | 39.71942 | WEAKLY DOMINATED |
| 9VHPV, GIRLS ONLY + CATCHUP | 44742.64256 | 39.72048 | 1,897,988.90 |

## Vaccine Doses Adherence

In this scenario, the baseline scenario was changed by assuming that the percentage of people who got a second does after their first, and a third dose after their second were lowered from 100% to 90%, as well as to 80%. While the values of the ICERs change in these scenarios, there is no impact on the cost effectiveness results. These can be seen in Table 36 and Table 37. The smaller ICERs are due largely to the decrease in cost due to less vaccine doses being given, while there is not a significant drop in effectiveness of the strategies.

Table 36 Costs, QALYs, and ICERs when adherence to 2 and 3 doses is lowered to 90%.

| Cervical Only | | |  |
| --- | --- | --- | --- |
| Strategy | Cost (¥) | QALY | ICER (¥/QALY) |
| No Vaccination | 34650.2 | 39.71734 | -- |
| 4VHPV, GIRLS ONLY | 39654.84 | 39.72146 | 1,216,314.31 |
| 9VHPV, GIRLS ONLY | 42559.19 | 39.72307 | Weakly Dominated |
| 9VHPV, GIRLS ONLY + CATCHUP | 43506.34 | 39.72399 | 1,519,177.83 |
| Cervical + GW | | |  |
| Strategy | Cost | QALY | ICER |
| No Vaccination | 34957.91 | 39.71584 | -- |
| 4VHPV, GIRLS ONLY | 39785.23 | 39.72081 | 971,775.28 |
| 9VHPV, GIRLS ONLY | 42689.58 | 39.72242 | Weakly Dominated |
| 9VHPV, GIRLS ONLY + CATCHUP | 43613.72 | 39.72345 | 1,447,088.15 |
| Cerivcal+GW+6/11CIN | | |  |
| Strategy | Cost | QALY | ICER |
| No Vaccination | 34986.71 | 39.71582 | -- |
| 4VHPV, GIRLS ONLY | 39798.14 | 39.7208 | 966,616.45 |
| 9VHPV, GIRLS ONLY | 42702.48 | 39.72241 | Weakly Dominated |
| 9VHPV, GIRLS ONLY + CATCHUP | 43624.41 | 39.72345 | 1,445,487.48 |
| Cervical+GW+6/11CIN+RRP | | |  |
| Strategy | Cost | QALY | ICER |
| No Vaccination | 35025.6 | 39.71567 | -- |
| 4VHPV, GIRLS ONLY | 39813.46 | 39.72069 | 954,169.00 |
| 9VHPV, GIRLS ONLY | 42717.8 | 39.7223 | Weakly Dominated |
| 9VHPV, GIRLS ONLY + CATCHUP | 43636.81 | 39.72335 | 1,441,137.74 |
| All Disease | | |  |
| Strategy | Cost | QALY | ICER |
| No Vaccination | 36455.42 | 39.7115 | -- |
| 4VHPV, GIRLS ONLY | 41094.82 | 39.71681 | 874,129.76 |
| 9VHPV, GIRLS ONLY | 43988.52 | 39.71844 | Weakly Dominated |
| 9VHPV, GIRLS ONLY + CATCHUP | 44874.68 | 39.71956 | 1,372,811.13 |

Table 37 Costs, QALYs, and ICERS when adherence to 2 and 3 doses is lowered to 80%.

| Cervical Only | | |  |
| --- | --- | --- | --- |
| Strategy | Cost (¥) | QALY | ICER (¥/QALY) |
| No Vaccination | 34650.2 | 39.71734 | -- |
| 4VHPV, GIRLS ONLY | 38965.84 | 39.72131 | 1,087,914.88 |
| 9VHPV, GIRLS ONLY | 41494.2 | 39.72288 | WEAKLY DOMINATED |
| 9VHPV, GIRLS ONLY + CATCHUP | 42303.76 | 39.72378 | 1,349,890.63 |
| Cervical + GW | | |  |
| Strategy | Cost | QALY | ICER |
| No Vaccination | 34957.91 | 39.71584 | -- |
| 4VHPV, GIRLS ONLY | 39104.66 | 39.72062 | 867,758.67 |
| 9VHPV, GIRLS ONLY | 41633.03 | 39.72219 | WEAKLY DOMINATED |
| 9VHPV, GIRLS ONLY + CATCHUP | 42420.57 | 39.7232 | 1,286,104.45 |
| Cerivcal+GW+6/11CIN | | |  |
| Strategy | Cost | QALY | ICER |
| No Vaccination | 34986.71 | 39.71582 | -- |
| 4VHPV, GIRLS ONLY | 39118.22 | 39.72061 | 862,827.17 |
| 9VHPV, GIRLS ONLY | 41646.59 | 39.72218 | WEAKLY DOMINATED |
| 9VHPV, GIRLS ONLY + CATCHUP | 42432 | 39.72319 | 1,284,605.31 |
| Cervical+GW+6/11CIN+RRP | | |  |
| Strategy | Cost | QALY | ICER |
| No Vaccination | 35025.6 | 39.71567 | -- |
| 4VHPV, GIRLS ONLY | 39134.5 | 39.7205 | 851,253.48 |
| 9VHPV, GIRLS ONLY | 41662.87 | 39.72207 | WEAKLY DOMINATED |
| 9VHPV, GIRLS ONLY + CATCHUP | 42445.46 | 39.72309 | 1,280,666.82 |
| All Disease | | |  |
| Strategy | Cost | QALY | ICER |
| No Vaccination | 36455.42 | 39.7115 | -- |
| 4VHPV, GIRLS ONLY | 40423.01 | 39.7166 | 777,574.25 |
| 9VHPV, GIRLS ONLY | 42941.14 | 39.7182 | WEAKLY DOMINATED |
| 9VHPV, GIRLS ONLY + CATCHUP | 43692.34 | 39.71928 | 1,219,280.91 |

## Duration of Protection

In the baseline scenario we assumed that the duration of the protection that is imparted by the vaccine is lifelong. To understand how this assumption impacts the economic results, we re-ran the baseline scenario with the duration of protection lowered, based on the dosage. So, in this scenario, a single dose imparts protection for 10 year, 2 doses 20 years, and 3 doses 30 years. The results are presented in Table 38.

Table 38 Costs, QALYs, and ICERs when the duration of protection is lowered.

| Cervical Only | | |  |
| --- | --- | --- | --- |
| Strategy | Cost (¥) | QALY | ICER (¥/QALY) |
| No Vaccination | 34650.19655 | 39.71734 | -- |
| 4VHPV, GIRLS ONLY | 41125.78241 | 39.72062 | 1,975,179.25 |
| 9VHPV, GIRLS ONLY | 44617.35669 | 39.722 | WEAKLY DOMINATED |
| 9VHPV, GIRLS ONLY + CATCHUP | 45762.75269 | 39.72287 | 2,059,350.09 |
| Cervical + GW | | |  |
| Strategy | Cost | QALY | ICER |
| No Vaccination | 34957.90853 | 39.71584 | -- |
| 4VHPV, GIRLS ONLY | 41309.4291 | 39.71972 | 1,638,409.85 |
| 9VHPV, GIRLS ONLY | 44801.00338 | 39.72109 | WEAKLY DOMINATED |
| 9VHPV, GIRLS ONLY + CATCHUP | 45928.17538 | 39.72206 | 1,974,613.28 |
| Cerivcal+GW+6/11CIN | | |  |
| Strategy | Cost | QALY | ICER |
| No Vaccination | 34986.71266 | 39.71582 | -- |
| 4VHPV, GIRLS ONLY | 41327.32764 | 39.71971 | 1,632,695.35 |
| 9VHPV, GIRLS ONLY | 44818.90192 | 39.72108 | WEAKLY DOMINATED |
| 9VHPV, GIRLS ONLY + CATCHUP | 45944.32063 | 39.72205 | 1,972,927.37 |
| Cervical+GW+6/11CIN+RRP | | |  |
| Strategy | Cost | QALY | ICER |
| No Vaccination | 35025.59731 | 39.71567 | -- |
| 4VHPV, GIRLS ONLY | 41348.75757 | 39.71959 | 1,615,607.29 |
| 9VHPV, GIRLS ONLY | 44840.33185 | 39.72096 | WEAKLY DOMINATED |
| 9VHPV, GIRLS ONLY + CATCHUP | 45963.27943 | 39.72193 | 1,967,595.38 |
| All Disease | | |  |
| Strategy | Cost | QALY | ICER |
| No Vaccination | 36455.41521 | 39.7115 | -- |
| 4VHPV, GIRLS ONLY | 42672.29095 | 39.71562 | 1,507,121.56 |
| 9VHPV, GIRLS ONLY | 46155.94105 | 39.71702 | WEAKLY DOMINATED |
| 9VHPV, GIRLS ONLY + CATCHUP | 47252.22001 | 39.71805 | 1,887,830.24 |

## Varied Vaccine Uptake in the Primary Cohort

For this scenario we altered the baseline scenario by changing the uptake in the primary cohort. First, we decreased in to 60%, and increased it to 100%. The results are included in Table 39-Table 40.

Table 39 costs, QALYs, and ICERs when the uptake in the primary cohort is lowered to 60%

| Cervical Only | | |  |
| --- | --- | --- | --- |
| Strategy | Cost (¥) | QALY | ICER (¥/QALY) |
| No Vaccination | 34650.19655 | 39.71734 | -- |
| 4VHPV, GIRLS ONLY | 39402.51241 | 39.7213 | 1,198,947.42 |
| 9VHPV, GIRLS ONLY | 42155.6417 | 39.72287 | WEAKLY DOMINATED |
| 9VHPV, GIRLS ONLY + CATCHUP | 43227.36983 | 39.72392 | 1,464,918.18 |
| Cervical + GW | | |  |
| Strategy | Cost | QALY | ICER |
| No Vaccination | 34957.90853 | 39.71584 | -- |
| 4VHPV, GIRLS ONLY | 39539.05918 | 39.72063 | 957,068.23 |
| 9VHPV, GIRLS ONLY | 42292.18847 | 39.72219 | WEAKLY DOMINATED |
| 9VHPV, GIRLS ONLY + CATCHUP | 43337.79931 | 39.72336 | 1,388,291.41 |
| Cerivcal+GW+6/11CIN | | |  |
| Strategy | Cost | QALY | ICER |
| No Vaccination | 34986.71266 | 39.71582 | -- |
| 4VHPV, GIRLS ONLY | 39552.4356 | 39.72062 | 951,904.22 |
| 9VHPV, GIRLS ONLY | 42305.56489 | 39.72218 | WEAKLY DOMINATED |
| 9VHPV, GIRLS ONLY + CATCHUP | 43348.67565 | 39.72336 | 1,386,572.01 |
| Cervical+GW+6/11CIN+RRP | | |  |
| Strategy | Cost | QALY | ICER |
| No Vaccination | 35025.59731 | 39.71567 | -- |
| 4VHPV, GIRLS ONLY | 39568.52688 | 39.72051 | 939,530.28 |
| 9VHPV, GIRLS ONLY | 42321.65616 | 39.72208 | WEAKLY DOMINATED |
| 9VHPV, GIRLS ONLY + CATCHUP | 43361.45224 | 39.72325 | 1,381,959.25 |
| All Disease | | |  |
| Strategy | Cost | QALY | ICER |
| No Vaccination | 36455.41521 | 39.7115 | -- |
| 4VHPV, GIRLS ONLY | 40857.37932 | 39.71661 | 861,404.03 |
| 9VHPV, GIRLS ONLY | 43600.34514 | 39.7182 | WEAKLY DOMINATED |
| 9VHPV, GIRLS ONLY + CATCHUP | 44603.41496 | 39.71946 | 1,313,380.02 |

Table 40 Costs, QALYs, and ICERs when the uptake in the primary cohort is raised to 80%

| Cervical Only | | |  |
| --- | --- | --- | --- |
| Strategy | Cost (¥) | QALY | ICER (¥/QALY) |
| No Vaccination | 34650.19655 | 39.71734089 | -- |
| 4VHPV, GIRLS ONLY | 41433.58279 | 39.72184239 | 1,506,917.99 |
| 9VHPV, GIRLS ONLY | 45303.5521 | 39.72356019 | WEAKLY DOMINATED |
| 9VHPV, GIRLS ONLY + CATCHUP | 46417.37694 | 39.72442555 | 1,929,337.76 |
| Cervical + GW | | |  |
| Strategy | Cost | QALY | ICER |
| No Vaccination | 34957.90853 | 39.71584041 | -- |
| 4VHPV, GIRLS ONLY | 41548.12948 | 39.72127228 | 1,213,249.92 |
| 9VHPV, GIRLS ONLY | 45418.09879 | 39.72299009 | WEAKLY DOMINATED |
| 9VHPV, GIRLS ONLY + CATCHUP | 46510.44161 | 39.7239585 | 1,847,319.39 |
| Cerivcal+GW+6/11CIN | | |  |
| Strategy | Cost | QALY | ICER |
| No Vaccination | 34986.71266 | 39.71582205 | -- |
| 4VHPV, GIRLS ONLY | 41559.81115 | 39.72126476 | 1,207,688.20 |
| 9VHPV, GIRLS ONLY | 45429.78046 | 39.72298256 | WEAKLY DOMINATED |
| 9VHPV, GIRLS ONLY + CATCHUP | 46520.06375 | 39.72395229 | 1,845,655.00 |
| Cervical+GW+6/11CIN+RRP | | |  |
| Strategy | Cost | QALY | ICER |
| No Vaccination | 35025.59731 | 39.71567476 | -- |
| 4VHPV, GIRLS ONLY | 41573.27995 | 39.72116119 | 1,193,432.04 |
| 9VHPV, GIRLS ONLY | 45443.24926 | 39.72287899 | WEAKLY DOMINATED |
| 9VHPV, GIRLS ONLY + CATCHUP | 46530.81196 | 39.7238543 | 1,840,820.82 |
| All Disease | | |  |
| Strategy | Cost | QALY | ICER |
| No Vaccination | 36455.41521 | 39.71149928 | -- |
| 4VHPV, GIRLS ONLY | 42833.95359 | 39.71731621 | 1,096,548.42 |
| 9VHPV, GIRLS ONLY | 46692.07856 | 39.71906373 | WEAKLY DOMINATED |
| 9VHPV, GIRLS ONLY + CATCHUP | 47747.41368 | 39.72011169 | 1,757,646.37 |

Table 41 Costs, QALYs, and ICERs when the uptake in the primary cohort is increased to 100%

| Cervical Only | | |  |
| --- | --- | --- | --- |
| Strategy | Cost (¥) | QALY | ICER (¥/QALY) |
| No Vaccination | 34650.19655 | 39.71734 | -- |
| 4VHPV, GIRLS ONLY | 43541.98494 | 39.72219 | 1,832,308.16 |
| 9VHPV, GIRLS ONLY | 48533.31425 | 39.72401 | WEAKLY DOMINATED |
| 9VHPV, GIRLS ONLY + CATCHUP | 49663.79404 | 39.72475 | 2,390,341.93 |
| Cervical + GW | | |  |
| Strategy | Cost | QALY | ICER |
| No Vaccination | 34957.90853 | 39.71584 | -- |
| 4VHPV, GIRLS ONLY | 43646.44219 | 39.72167 | 1,489,675.72 |
| 9VHPV, GIRLS ONLY | 48637.7715 | 39.72349 | WEAKLY DOMINATED |
| 9VHPV, GIRLS ONLY + CATCHUP | 49750.03636 | 39.72432 | 2,304,669.37 |
| Cerivcal+GW+6/11CIN | | |  |
| Strategy | Cost | QALY | ICER |
| No Vaccination | 34986.71266 | 39.71582 | -- |
| 4VHPV, GIRLS ONLY | 43657.34372 | 39.72167 | 1,483,724.04 |
| 9VHPV, GIRLS ONLY | 48648.67304 | 39.72348 | WEAKLY DOMINATED |
| 9VHPV, GIRLS ONLY + CATCHUP | 49759.1739 | 39.72432 | 2,303,029.34 |
| Cervical+GW+6/11CIN+RRP | | |  |
| Strategy | Cost | QALY | ICER |
| No Vaccination | 35025.59731 | 39.71567 | -- |
| 4VHPV, GIRLS ONLY | 43669.60638 | 39.72156 | 1,467,583.66 |
| 9VHPV, GIRLS ONLY | 48660.93569 | 39.72338 | WEAKLY DOMINATED |
| 9VHPV, GIRLS ONLY + CATCHUP | 49769.124 | 39.72422 | 2,298,019.26 |
| All Disease | | |  |
| Strategy | Cost | QALY | ICER |
| No Vaccination | 36455.41521 | 39.7115 | -- |
| 4VHPV, GIRLS ONLY | 44908.38324 | 39.71776 | 1,349,381.02 |
| 9VHPV, GIRLS ONLY | 49886.59865 | 39.71962 | WEAKLY DOMINATED |
| 9VHPV, GIRLS ONLY + CATCHUP | 50966.10948 | 39.72052 | 2,200,880.77 |

## Utilities

We varied the utilities to understand their impact on the ICERs. In two separate scenarios the healthy utilities were decreased by 20%, and the utilities associated with disease were increased by 20%. Both adjustments tended to raise the ICER. In the first case, because by decreasing the healthy utilities, less are lost due to death by disease. In the second, by bringing the disease utilities up we are assuming that disease is less impactful to quality of life. However, neither of these changes significantly changed the result. Healthy utilities were increased by 20%, to a max of 1, and the disease utilities decreased by 20%.

Table 42 Costs, QALYs, and ICERs when the healthy utilities are decreased by 20%.

| Cervical Only | | |  |
| --- | --- | --- | --- |
| Strategy | Cost (¥) | QALY | ICER (¥/QALY) |
| No Vaccination | 34650.19656 | 31.77387 | -- |
| 4VHPV, GIRLS ONLY | 40403.67832 | 31.77728 | 1,687,266.12 |
| 9VHPV, GIRLS ONLY | 43713.54674 | 31.7786 | WEAKLY DOMINATED |
| 9VHPV, GIRLS ONLY + CATCHUP | 44810.37396 | 31.77936 | 2,121,144.00 |
| Cervical + GW | | |  |
| Strategy | Cost | QALY | ICER |
| No Vaccination | 34957.90853 | 31.77267 | -- |
| 4VHPV, GIRLS ONLY | 40526.74055 | 31.77679 | 1,351,358.56 |
| 9VHPV, GIRLS ONLY | 43836.60897 | 31.77811 | WEAKLY DOMINATED |
| 9VHPV, GIRLS ONLY + CATCHUP | 44909.69281 | 31.77896 | 2,021,034.88 |
| Cerivcal+GW+6/11CIN | | |  |
| Strategy | Cost | QALY | ICER |
| No Vaccination | 34986.71266 | 31.77266 | -- |
| 4VHPV, GIRLS ONLY | 40539.07547 | 31.77679 | 1,344,641.25 |
| 9VHPV, GIRLS ONLY | 43848.94389 | 31.77811 | WEAKLY DOMINATED |
| 9VHPV, GIRLS ONLY + CATCHUP | 44919.7602 | 31.77896 | 2,018,917.96 |
| Cervical+GW+6/11CIN+RRP | | |  |
| Strategy | Cost | QALY | ICER |
| No Vaccination | 35025.59732 | 31.77254 | -- |
| 4VHPV, GIRLS ONLY | 40553.55994 | 31.7767 | 1,327,983.68 |
| 9VHPV, GIRLS ONLY | 43863.42837 | 31.77802 | WEAKLY DOMINATED |
| 9VHPV, GIRLS ONLY + CATCHUP | 44931.23897 | 31.77888 | 2,012,980.23 |
| All Disease | | |  |
| Strategy | Cost | QALY | ICER |
| No Vaccination | 36455.41522 | 31.7692 | -- |
| 4VHPV, GIRLS ONLY | 41827.43034 | 31.77361 | 1,219,204.47 |
| 9VHPV, GIRLS ONLY | 45126.23264 | 31.77495 | WEAKLY DOMINATED |
| 9VHPV, GIRLS ONLY + CATCHUP | 46159.69994 | 31.77586 | 1,918,117.10 |

Table 43 Costs, QALYs, and ICERS when the disease utilities are increased 20%

| Cervical Only | | |  |
| --- | --- | --- | --- |
| Strategy | Cost (¥) | QALY | ICER (¥/QALY) |
| No Vaccination | 34650.19656 | 39.730616 | -- |
| 4VHPV, GIRLS ONLY | 40403.67831 | 39.73277158 | 2,669,111.69 |
| 9VHPV, GIRLS ONLY | 43713.54665 | 39.73360317 | WEAKLY DOMINATED |
| 9VHPV, GIRLS ONLY + CATCHUP | 44810.37397 | 39.73409412 | 3,331,970.78 |
| Cervical + GW | | |  |
| Strategy | Cost | QALY | ICER |
| No Vaccination | 34957.90853 | 39.730616 | -- |
| 4VHPV, GIRLS ONLY | 40526.74054 | 39.73277158 | 2,583,450.38 |
| 9VHPV, GIRLS ONLY | 43836.60888 | 39.73360317 | WEAKLY DOMINATED |
| 9VHPV, GIRLS ONLY + CATCHUP | 44909.69282 | 39.73409412 | 3,314,018.05 |
| Cerivcal+GW+6/11CIN | | |  |
| Strategy | Cost | QALY | ICER |
| No Vaccination | 34986.71266 | 39.730616 | -- |
| 4VHPV, GIRLS ONLY | 40539.07546 | 39.73277158 | 2,575,810.11 |
| 9VHPV, GIRLS ONLY | 43848.9438 | 39.73360317 | WEAKLY DOMINATED |
| 9VHPV, GIRLS ONLY + CATCHUP | 44919.76022 | 39.73409412 | 3,312,303.53 |
| Cervical+GW+6/11CIN+RRP | | |  |
| Strategy | Cost | QALY | ICER |
| No Vaccination | 35025.59732 | 39.73049305 | -- |
| 4VHPV, GIRLS ONLY | 40553.55993 | 39.73267678 | 2,531,434.57 |
| 9VHPV, GIRLS ONLY | 43863.42827 | 39.73350837 | WEAKLY DOMINATED |
| 9VHPV, GIRLS ONLY + CATCHUP | 44931.23899 | 39.73400374 | 3,299,019.09 |
| All Disease | | |  |
| Strategy | Cost | QALY | ICER |
| No Vaccination | 36455.41522 | 39.72738513 | -- |
| 4VHPV, GIRLS ONLY | 41827.43033 | 39.7297866 | 2,236,971.07 |
| 9VHPV, GIRLS ONLY | 45126.23255 | 39.7306374 | WEAKLY DOMINATED |
| 9VHPV, GIRLS ONLY + CATCHUP | 46159.69994 | 39.73118878 | 3,089,664.16 |

Table 44 Costs, QALYs, and ICERs when the healthy utilities are increased by 20%

| Cervical Only | | |  |
| --- | --- | --- | --- |
| Strategy | Cost (¥) | QALY | ICER (¥/QALY) |
| No Vaccination | 34650.19653 | 43.30880932 | -- |
| 4VHPV, GIRLS ONLY | 40403.67831 | 43.31360346 | 1,200,107.98 |
| 9VHPV, GIRLS ONLY | 43713.54672 | 43.31546155 | WEAKLY DOMINATED |
| 9VHPV, GIRLS ONLY + CATCHUP | 44810.37397 | 43.31653788 | 1,501,727.72 |
| Cervical + GW | | |  |
| Strategy | Cost | QALY | ICER |
| No Vaccination | 34957.9085 | 43.30721324 | -- |
| 4VHPV, GIRLS ONLY | 40526.74054 | 43.31295235 | 970,331.61 |
| 9VHPV, GIRLS ONLY | 43836.60896 | 43.31481044 | WEAKLY DOMINATED |
| 9VHPV, GIRLS ONLY + CATCHUP | 44909.69282 | 43.31600799 | 1,434,379.47 |
| Cerivcal+GW+6/11CIN | | |  |
| Strategy | Cost | QALY | ICER |
| No Vaccination | 34986.71263 | 43.30719328 | -- |
| 4VHPV, GIRLS ONLY | 40539.07547 | 43.31294371 | 965,556.00 |
| 9VHPV, GIRLS ONLY | 43848.94388 | 43.3148018 | WEAKLY DOMINATED |
| 9VHPV, GIRLS ONLY + CATCHUP | 44919.76021 | 43.31600091 | 1,432,903.96 |
| Cervical+GW+6/11CIN+RRP | | |  |
| Strategy | Cost | QALY | ICER |
| No Vaccination | 35025.59729 | 43.30703362 | -- |
| 4VHPV, GIRLS ONLY | 40553.55994 | 43.31282841 | 953,953.39 |
| 9VHPV, GIRLS ONLY | 43863.42836 | 43.31468651 | WEAKLY DOMINATED |
| 9VHPV, GIRLS ONLY + CATCHUP | 44931.23898 | 43.31589216 | 1,428,863.80 |
| All Disease | | |  |
| Strategy | Cost | QALY | ICER |
| No Vaccination | 36455.41518 | 43.30219312 | -- |
| 4VHPV, GIRLS ONLY | 41827.43034 | 43.30833578 | 874,541.87 |
| 9VHPV, GIRLS ONLY | 45126.23263 | 43.31022573 | WEAKLY DOMINATED |
| 9VHPV, GIRLS ONLY + CATCHUP | 46159.69994 | 43.31152035 | 1,360,391.80 |

Table 45 Costs, QALYs, and ICERS when the disease utilities are decreased 20%

| Cervical Only | | |  |
| --- | --- | --- | --- |
| Strategy | Cost (¥) | QALY | ICER (¥/QALY) |
| No Vaccination | 34650.19655 | 39.70343965 | -- |
| 4VHPV, GIRLS ONLY | 40403.67832 | 39.71001182 | 875,430.58 |
| 9VHPV, GIRLS ONLY | 43713.54669 | 39.71256199 | WEAKLY DOMINATED |
| 9VHPV, GIRLS ONLY + CATCHUP | 44810.37393 | 39.7140047 | 1,103,638.17 |
| Cervical + GW | | |  |
| Strategy | Cost | QALY | ICER |
| No Vaccination | 34957.90853 | 39.69890717 | -- |
| 4VHPV, GIRLS ONLY | 40526.74055 | 39.7081639 | 601,598.38 |
| 9VHPV, GIRLS ONLY | 43836.60891 | 39.71071407 | WEAKLY DOMINATED |
| 9VHPV, GIRLS ONLY + CATCHUP | 44909.69278 | 39.71250097 | 1,010,578.30 |
| Cerivcal+GW+6/11CIN | | |  |
| Strategy | Cost | QALY | ICER |
| No Vaccination | 34986.71266 | 39.69885401 | -- |
| 4VHPV, GIRLS ONLY | 40539.07547 | 39.7081409 | 597,870.86 |
| 9VHPV, GIRLS ONLY | 43848.94384 | 39.71069107 | WEAKLY DOMINATED |
| 9VHPV, GIRLS ONLY + CATCHUP | 44919.76017 | 39.71248214 | 1,009,086.65 |
| Cervical+GW+6/11CIN+RRP | | |  |
| Strategy | Cost | QALY | ICER |
| No Vaccination | 35025.59731 | 39.69868238 | -- |
| 4VHPV, GIRLS ONLY | 40553.55994 | 39.70802467 | 591,713.32 |
| 9VHPV, GIRLS ONLY | 43863.42831 | 39.71057484 | WEAKLY DOMINATED |
| 9VHPV, GIRLS ONLY + CATCHUP | 44931.23895 | 39.71237376 | 1,006,573.36 |
| All Disease | | |  |
| Strategy | Cost | QALY | ICER |
| No Vaccination | 36455.41521 | 39.69343935 | -- |
| 4VHPV, GIRLS ONLY | 41827.43034 | 39.70317263 | 551,922.15 |
| 9VHPV, GIRLS ONLY | 45126.23258 | 39.70575893 | WEAKLY DOMINATED |
| 9VHPV, GIRLS ONLY + CATCHUP | 46159.69991 | 39.70765621 | 966,253.71 |

## Discount Rates

Discount rates were varied to understand their impact on the ICER. In addition to the baseline scenario value of 2% we ran them at 0% and 4%. The results are shown below Table 45 and Table 46.

Table 46 Cost, QALYS, and ICERs when the discount rate is raised to 4%

| Cervical Only | | |  |
| --- | --- | --- | --- |
| Strategy | Cost (¥) | QALY | ICER (¥/QALY) |
| No Vaccination | 19894.68418 | 22.80402542 | -- |
| 4VHPV, GIRLS ONLY | 24124.63843 | 22.80523066 | 3,509,620.73 |
| 9VHPV, GIRLS ONLY | 26398.65976 | 22.80569992 | WEAKLY DOMINATED |
| 9VHPV, GIRLS ONLY + CATCHUP | 27722.49888 | 22.80607144 | 4,279,231.15 |
| Cervical + GW | | |  |
| Strategy | Cost | QALY | ICER |
| No Vaccination | 20071.35944 | 22.8031639 | -- |
| 4VHPV, GIRLS ONLY | 24221.77993 | 22.80474923 | 2,618,016.35 |
| 9VHPV, GIRLS ONLY | 26495.80126 | 22.80521849 | WEAKLY DOMINATED |
| 9VHPV, GIRLS ONLY + CATCHUP | 27802.91242 | 22.80566948 | 3,891,495.70 |
| Cerivcal+GW+6/11CIN | | |  |
| Strategy | Cost | QALY | ICER |
| No Vaccination | 20087.89756 | 22.80315336 | -- |
| 4VHPV, GIRLS ONLY | 24231.52489 | 22.80474297 | 2,606,692.45 |
| 9VHPV, GIRLS ONLY | 26505.54622 | 22.80521223 | WEAKLY DOMINATED |
| 9VHPV, GIRLS ONLY + CATCHUP | 27811.11348 | 22.80566419 | 3,885,704.29 |
| Cervical+GW+6/11CIN+RRP | | |  |
| Strategy | Cost | QALY | ICER |
| No Vaccination | 20110.22349 | 22.8030688 | -- |
| 4VHPV, GIRLS ONLY | 24243.13173 | 22.80467313 | 2,576,094.43 |
| 9VHPV, GIRLS ONLY | 26517.15305 | 22.80514238 | WEAKLY DOMINATED |
| 9VHPV, GIRLS ONLY + CATCHUP | 27820.5458 | 22.8055975 | 3,870,079.15 |
| All Disease | | |  |
| Strategy | Cost | QALY | ICER |
| No Vaccination | 20931.18834 | 22.80067131 | -- |
| 4VHPV, GIRLS ONLY | 25025.12745 | 22.80234604 | 2,444,532.10 |
| 9VHPV, GIRLS ONLY | 27296.37955 | 22.80282166 | WEAKLY DOMINATED |
| 9VHPV, GIRLS ONLY + CATCHUP | 28588.62167 | 22.80329885 | 3,740,011.14 |

Table 47 Cost, QALYS, and ICERs when the discount rate is raised to 3%

| Cervical Only | | |  |
| --- | --- | --- | --- |
| Strategy | Cost (¥) | QALY | ICER (¥/QALY) |
| No Vaccination | 25529.61834 | 29.26299566 | -- |
| 4VHPV, GIRLS ONLY | 30414.5537 | 29.26521486 | 2,201,214.51 |
| 9VHPV, GIRLS ONLY | 33110.87374 | 29.26607639 | WEAKLY DOMINATED |
| 9VHPV, GIRLS ONLY + CATCHUP | 34350.32477 | 29.26665828 | 2,726,703.85 |
| Cervical + GW | | |  |
| Strategy | Cost | QALY | ICER |
| No Vaccination | 25756.33479 | 29.26189013 | -- |
| 4VHPV, GIRLS ONLY | 30523.1987 | 29.26467566 | 1,711,293.81 |
| 9VHPV, GIRLS ONLY | 33219.51874 | 29.26553719 | WEAKLY DOMINATED |
| 9VHPV, GIRLS ONLY + CATCHUP | 34439.17177 | 29.2662136 | 2,546,245.17 |
| Cerivcal+GW+6/11CIN | | |  |
| Strategy | Cost | QALY | ICER |
| No Vaccination | 25777.55713 | 29.2618766 | -- |
| 4VHPV, GIRLS ONLY | 30534.10071 | 29.26466865 | 1,703,602.42 |
| 9VHPV, GIRLS ONLY | 33230.42076 | 29.26553018 | WEAKLY DOMINATED |
| 9VHPV, GIRLS ONLY + CATCHUP | 34448.21333 | 29.26620777 | 2,543,089.06 |
| Cervical+GW+6/11CIN+RRP | | |  |
| Strategy | Cost | QALY | ICER |
| No Vaccination | 25806.20662 | 29.26176808 | -- |
| 4VHPV, GIRLS ONLY | 30546.99129 | 29.26458427 | 1,683,405.74 |
| 9VHPV, GIRLS ONLY | 33243.31133 | 29.2654458 | WEAKLY DOMINATED |
| 9VHPV, GIRLS ONLY + CATCHUP | 34458.56202 | 29.26612769 | 2,534,345.40 |
| All Disease | | |  |
| Strategy | Cost | QALY | ICER |
| No Vaccination | 26859.68665 | 29.2586916 | -- |
| 4VHPV, GIRLS ONLY | 31523.87291 | 29.26165192 | 1,575,569.94 |
| 9VHPV, GIRLS ONLY | 34214.75332 | 29.26252651 | WEAKLY DOMINATED |
| 9VHPV, GIRLS ONLY + CATCHUP | 35410.77672 | 29.26324898 | 2,433,779.18 |

Table 48 Cost, QALYs, and ICERs when discount rate is decreased to 0%

| Cervical Only | | |  |
| --- | --- | --- | --- |
| Strategy | Cost (¥) | QALY | ICER (¥/QALY) |
| No Vaccination | 79604.54459 | 84.4734975 | -- |
| 4VHPV, GIRLS ONLY | 88169.72503 | 86.25214914 | 4,815.55 |
| 9VHPV, GIRLS ONLY | 93978.55543 | 86.93727212 | WEAKLY DOMINATED |
| 9VHPV, GIRLS ONLY + CATCHUP | 94422.43987 | 87.22123601 | 6,452.17 |
| Cervical + GW | | |  |
| Strategy | Cost | QALY | ICER |
| No Vaccination | 80311.47483 | 84.12877966 | -- |
| 4VHPV, GIRLS ONLY | 88336.03306 | 86.16910127 | 3,932.99 |
| 9VHPV, GIRLS ONLY | 94144.86347 | 86.85422426 | WEAKLY DOMINATED |
| 9VHPV, GIRLS ONLY + CATCHUP | 94552.80641 | 87.1556353 | 6,301.63 |
| Cerivcal+GW+6/11CIN | | |  |
| Strategy | Cost | QALY | ICER |
| No Vaccination | 80377.64875 | 84.12456188 | -- |
| 4VHPV, GIRLS ONLY | 88352.54137 | 86.16803726 | 3,902.61 |
| 9VHPV, GIRLS ONLY | 94161.37178 | 86.85316024 | WEAKLY DOMINATED |
| 9VHPV, GIRLS ONLY + CATCHUP | 94565.80132 | 87.15479539 | 6,296.64 |
| Cervical+GW+6/11CIN+RRP | | |  |
| Strategy | Cost | QALY | ICER |
| No Vaccination | 80466.98144 | 84.09072416 | -- |
| 4VHPV, GIRLS ONLY | 88371.74118 | 86.14900325 | 3,840.47 |
| 9VHPV, GIRLS ONLY | 94180.57159 | 86.83412623 | WEAKLY DOMINATED |
| 9VHPV, GIRLS ONLY + CATCHUP | 94580.60349 | 87.13722831 | 6,282.84 |
| All Disease | | |  |
| Strategy | Cost | QALY | ICER |
| No Vaccination | 83751.67881 | 83.13151917 | -- |
| 4VHPV, GIRLS ONLY | 90941.62828 | 85.33799435 | 3,258.57 |
| 9VHPV, GIRLS ONLY | 96699.82297 | 86.036637 | WEAKLY DOMINATED |
| 9VHPV, GIRLS ONLY + CATCHUP | 96977.37921 | 86.37070091 | 5,844.59 |

## Price

The price of 9vHPV was increased and decreased by 10% to assess its impact on the ICER. The results are given below in Table 47 and Table 48.

Table 49 Costs, QALYs, and ICERs when the price is increased 10%

| Cervical Only | | |  |
| --- | --- | --- | --- |
| Strategy | Cost (¥) | QALY | ICER (¥/QALY) |
| No Vaccination | 34650.19655 | 39.7173409 | -- |
| 4VHPV, GIRLS ONLY | 40403.67831 | 39.7216033 | 1,349,812.89 |
| 9VHPV, GIRLS ONLY | 44822.0172 | 39.7232535 | WEAKLY DOMINATED |
| 9VHPV, GIRLS ONLY + CATCHUP | 46060.4129 | 39.7242002 | 2,178,275.88 |
| Cervical + GW | | |  |
| Strategy | Cost | QALY | ICER |
| No Vaccination | 34957.90853 | 39.71584041 | -- |
| 4VHPV, GIRLS ONLY | 40526.74054 | 39.72099155 | 1,081,086.85 |
| 9VHPV, GIRLS ONLY | 44945.07943 | 39.72264169 | WEAKLY DOMINATED |
| 9VHPV, GIRLS ONLY + CATCHUP | 46159.73175 | 39.72370238 | 2,077,954.93 |
| Cerivcal+GW+6/11CIN | | |  |
| Strategy | Cost | QALY | ICER |
| No Vaccination | 34986.71266 | 39.71582205 | -- |
| 4VHPV, GIRLS ONLY | 40539.07547 | 39.72098361 | 1,075,713.00 |
| 9VHPV, GIRLS ONLY | 44957.41436 | 39.72263375 | WEAKLY DOMINATED |
| 9VHPV, GIRLS ONLY + CATCHUP | 46169.79914 | 39.72369588 | 2,076,016.82 |
| Cervical+GW+6/11CIN+RRP | | |  |
| Strategy | Cost | QALY | ICER |
| No Vaccination | 35025.59731 | 39.71567476 | -- |
| 4VHPV, GIRLS ONLY | 40553.55994 | 39.7208781 | 1,062,386.94 |
| 9VHPV, GIRLS ONLY | 44971.89883 | 39.72252824 | WEAKLY DOMINATED |
| 9VHPV, GIRLS ONLY + CATCHUP | 46181.27791 | 39.72359651 | 2,070,226.67 |
| All Disease | | |  |
| Strategy | Cost | QALY | ICER |
| No Vaccination | 36455.41521 | 39.71149928 | -- |
| 4VHPV, GIRLS ONLY | 41827.43034 | 39.71700699 | 975,363.58 |
| 9VHPV, GIRLS ONLY | 46234.70311 | 39.7186848 | WEAKLY DOMINATED |
| 9VHPV, GIRLS ONLY + CATCHUP | 47409.73887 | 39.71983025 | 1,977,258.54 |

Table 50 Costs, QALYs, and ICERs when the price is decreased 10%

| Cervical Only | | |  |
| --- | --- | --- | --- |
| Strategy | Cost (¥) | QALY | ICER (¥/QALY) |
| No Vaccination | 34650.1966 | 39.71734089 | -- |
| 4VHPV, GIRLS ONLY | 40403.6783 | 39.72160332 | 1,349,812.89 |
| 9VHPV, GIRLS ONLY | 42605.0762 | 39.72325346 | WEAKLY DOMINATED |
| 9VHPV, GIRLS ONLY + CATCHUP | 43560.3349 | 39.72420021 | 1,215,554.47 |
| Cervical + GW | | |  |
| Strategy | Cost | QALY | ICER |
| No Vaccination | 34957.90853 | 39.71584041 | -- |
| 4VHPV, GIRLS ONLY | 40526.74054 | 39.72099155 | 1,081,086.85 |
| 9VHPV, GIRLS ONLY | 42728.13848 | 39.72264169 | WEAKLY DOMINATED |
| 9VHPV, GIRLS ONLY + CATCHUP | 43659.6538 | 39.72370238 | 1,155,700.82 |
| Cerivcal+GW+6/11CIN | | |  |
| Strategy | Cost | QALY | ICER |
| No Vaccination | 34986.71266 | 39.71582205 | -- |
| 4VHPV, GIRLS ONLY | 40539.07547 | 39.72098361 | 1,075,713.00 |
| 9VHPV, GIRLS ONLY | 42740.4734 | 39.72263375 | WEAKLY DOMINATED |
| 9VHPV, GIRLS ONLY + CATCHUP | 43669.72119 | 39.72369588 | 1,154,251.85 |
| Cervical+GW+6/11CIN+RRP | | |  |
| Strategy | Cost | QALY | ICER |
| No Vaccination | 35025.59731 | 39.71567476 | -- |
| 4VHPV, GIRLS ONLY | 40553.55994 | 39.7208781 | 1,062,386.94 |
| 9VHPV, GIRLS ONLY | 42754.95788 | 39.72252824 | WEAKLY DOMINATED |
| 9VHPV, GIRLS ONLY + CATCHUP | 43681.19996 | 39.72359651 | 1,150,541.63 |
| All Disease | | |  |
| Strategy | Cost | QALY | ICER |
| No Vaccination | 36455.41521 | 39.71149928 | -- |
| 4VHPV, GIRLS ONLY | 41827.43034 | 39.71700699 | 975,363.58 |
| 9VHPV, GIRLS ONLY | 44017.76215 | 39.7186848 | WEAKLY DOMINATED |
| 9VHPV, GIRLS ONLY + CATCHUP | 44909.66093 | 39.71983025 | 1,091,728.76 |

## Disease cost

The cost of disease treatment was varied as well, increasing and decreasing by 20%. See Table 49

Table 51 Costs, QALYs, and ICERs when the cost of disease is lowered 20%

| Cervical Only | | |  |
| --- | --- | --- | --- |
| Strategy | Cost (¥) | QALY | ICER (¥/QALY) |
| No Vaccination | 27720.15728 | 39.71734089 | -- |
| 4VHPV, GIRLS ONLY | 34065.23655 | 39.72160332 | 1,488,606.40 |
| 9VHPV, GIRLS ONLY | 37599.90765 | 39.72325346 | WEAKLY DOMINATED |
| 9VHPV, GIRLS ONLY + CATCHUP | 38813.14159 | 39.72420021 | 1,828,306.92 |
| Cervical + GW | | |  |
| Strategy | Cost | QALY | ICER |
| No Vaccination | 27966.32686 | 39.71584041 | -- |
| 4VHPV, GIRLS ONLY | 34163.68633 | 39.72099155 | 1,203,103.95 |
| 9VHPV, GIRLS ONLY | 37698.35743 | 39.72264169 | WEAKLY DOMINATED |
| 9VHPV, GIRLS ONLY + CATCHUP | 38892.59667 | 39.72370238 | 1,744,448.43 |
| Cerivcal+GW+6/11CIN | | |  |
| Strategy | Cost | QALY | ICER |
| No Vaccination | 27989.37016 | 39.71582205 | -- |
| 4VHPV, GIRLS ONLY | 34173.55427 | 39.72098361 | 1,198,121.85 |
| 9VHPV, GIRLS ONLY | 37708.22537 | 39.72263375 | WEAKLY DOMINATED |
| 9VHPV, GIRLS ONLY + CATCHUP | 38900.65058 | 39.72369588 | 1,742,854.42 |
| Cervical+GW+6/11CIN+RRP | | |  |
| Strategy | Cost | QALY | ICER |
| No Vaccination | 28020.47789 | 39.71567476 | -- |
| 4VHPV, GIRLS ONLY | 34185.14185 | 39.7208781 | 1,184,750.85 |
| 9VHPV, GIRLS ONLY | 37719.81294 | 39.72252824 | WEAKLY DOMINATED |
| 9VHPV, GIRLS ONLY + CATCHUP | 38909.8336 | 39.72359651 | 1,738,037.17 |
| All Disease | | |  |
| Strategy | Cost | QALY | ICER |
| No Vaccination | 29164.33221 | 39.71149928 | -- |
| 4VHPV, GIRLS ONLY | 35204.23818 | 39.71700699 | 1,096,628.38 |
| 9VHPV, GIRLS ONLY | 38730.05637 | 39.7186848 | WEAKLY DOMINATED |
| 9VHPV, GIRLS ONLY + CATCHUP | 39892.60237 | 39.71983025 | 1,660,622.69 |

Table 52 Costs, QALYs, and ICERs when the cost of disease is raised 20%

| Cervical Only | | |  |
| --- | --- | --- | --- |
| Strategy | Cost (¥) | QALY | ICER (¥/QALY) |
| No Vaccination | 41580.23587 | 39.71734089 | -- |
| 4VHPV, GIRLS ONLY | 46742.12007 | 39.72160332 | 1,211,019.37 |
| 9VHPV, GIRLS ONLY | 49827.18579 | 39.72325346 | WEAKLY DOMINATED |
| 9VHPV, GIRLS ONLY + CATCHUP | 50807.60627 | 39.72420021 | 1,565,523.44 |
| Cervical + GW | | |  |
| Strategy | Cost | QALY | ICER |
| No Vaccination | 41949.49024 | 39.71584041 | -- |
| 4VHPV, GIRLS ONLY | 46889.79475 | 39.72099155 | 959,069.74 |
| 9VHPV, GIRLS ONLY | 49974.86047 | 39.72264169 | WEAKLY DOMINATED |
| 9VHPV, GIRLS ONLY + CATCHUP | 50926.7889 | 39.72370238 | 1,489,207.36 |
| Cerivcal+GW+6/11CIN | | |  |
| Strategy | Cost | QALY | ICER |
| No Vaccination | 41984.05519 | 39.71582205 | -- |
| 4VHPV, GIRLS ONLY | 46904.59666 | 39.72098361 | 953,304.14 |
| 9VHPV, GIRLS ONLY | 49989.66238 | 39.72263375 | WEAKLY DOMINATED |
| 9VHPV, GIRLS ONLY + CATCHUP | 50938.86977 | 39.72369588 | 1,487,414.30 |
| Cervical+GW+6/11CIN+RRP | | |  |
| Strategy | Cost | QALY | ICER |
| No Vaccination | 42030.71678 | 39.71567476 | -- |
| 4VHPV, GIRLS ONLY | 46921.97803 | 39.7208781 | 940,023.02 |
| 9VHPV, GIRLS ONLY | 50007.04375 | 39.72252824 | WEAKLY DOMINATED |
| 9VHPV, GIRLS ONLY + CATCHUP | 50952.6443 | 39.72359651 | 1,482,731.18 |
| All Disease | | |  |
| Strategy | Cost | QALY | ICER |
| No Vaccination | 43746.49826 | 39.71149928 | -- |
| 4VHPV, GIRLS ONLY | 48450.6225 | 39.71700699 | 854,098.76 |
| 9VHPV, GIRLS ONLY | 51522.40887 | 39.7186848 | WEAKLY DOMINATED |
| 9VHPV, GIRLS ONLY + CATCHUP | 52426.79745 | 39.71983025 | 1,408,364.64 |

# References

[1] E. H. Elbasha and E. J. Dasbach, "Impact of vaccinating boys and men against HPV in the United States," *Vaccine,* vol. 28, no. 42, pp. 6858-67, Oct 4 2010, doi: 10.1016/j.vaccine.2010.08.030.

[2] E. H. Elbasha, E. J. Dasbach, and R. P. Insinga, "Model for assessing human papillomavirus vaccination strategies," *Emerging infectious diseases,* vol. 13, no. 1, pp. 28-41, Jan 2007, doi: 10.3201/eid1301.060438.

[3] M. S. Longworth and L. A. Laimins, "Pathogenesis of human papillomaviruses in differentiating epithelia," *Microbiol.Mol.Biol.Rev.,* vol. 68, no. 2, pp. 362-372, 6/2004 2004, doi: 10.1128/MMBR.68.2.362-372.2004 [doi];68/2/362 [pii].

[4] "Genital warts," National Health Services, 1/6/2014 2015. [Online]. Available: <http://www.nhs.uk/conditions/genital_warts/pages/introduction.aspx>

[5] J. T. Schiller, P. M. Day, and R. C. Kines, "Current understanding of the mechanism of HPV infection," *Gynecol.Oncol.,* vol. 118, no. 1 Suppl, pp. S12-S17, 6/2010 2010, doi: S0090-8258(10)00290-8 [pii];10.1016/j.ygyno.2010.04.004 [doi].

[6] "Genital HPV infection - CDC fact sheet," Centers for Disease Control and Prevention, 2015 2015, vol. 2014. [Online]. Available: <http://www.cdc.gov/std/hpv/stdfact-hpv.htm>

[7] E. M. Smith, M. A. Parker, L. M. Rubenstein, T. H. Haugen, E. Hamsikova, and L. P. Turek, "Evidence for vertical transmission of HPV from mothers to infants," *Infect Dis Obstet Gynecol,* vol. 2010, p. 326369, 2010, doi: 10.1155/2010/326369.

[8] S. M. Lee *et al.*, "Risk of vertical transmission of human papillomavirus throughout pregnancy: a prospective study," *PLoS One,* vol. 8, no. 6, p. e66368, 2013, doi: 10.1371/journal.pone.0066368.

[9] C. R. UK. "Does HPV cause cancer?" Cancer Research UK. <https://www.cancerresearchuk.org/about-cancer/causes-of-cancer/infections-eg-hpv-and-cancer/does-hpv-cause-cancer> (accessed January 13, 2020).

[10] Centers for Disease Control and Prevention. "Human Papillomavirus (HPV) - HPV Vaccine Schedule and Dosing." Centers for Disease Control and Prevention. <https://www.cdc.gov/hpv/hcp/schedules-recommendations.html> (accessed 12/14/2019.

[11] F. Bray, J. Ferlay, I. Soerjomataram, R. L. Siegel, L. A. Torre, and A. Jemal, "Global cancer statistics 2018: GLOBOCAN estimates of incidence and mortality worldwide for 36 cancers in 185 countries," *CA Cancer J Clin,* vol. 68, no. 6, pp. 394-424, Nov 2018, doi: 10.3322/caac.21492.

[12] B. Serrano, M. Brotons, F. X. Bosch, and L. Bruni, "Epidemiology and burden of HPV-related disease," *Best practice & research. Clinical obstetrics & gynaecology,* vol. 47, pp. 14-26, Feb 2018, doi: 10.1016/j.bpobgyn.2017.08.006.

[13] "National Cancer Registry, Japan." <https://ganjoho.jp/reg_stat/statistics/stat/summary.html> (accessed June 25, 2020.

[14] B. F. C. M. M. L. P. M. Z. A. Z. R. F. J. editors, "Cancer Incidence in Five Continents, Vol XI (Electronic Version)," 2017. [Online]. Available: <http://ci5.iarc.fr>.

[15] F. Aubin *et al.*, "Human papillomavirus genotype distribution in external acuminata condylomata: a Large French National Study (EDiTH IV)," *Clin Infect.Dis.,* vol. 47, no. 5, pp. 610-615, 9/1/2008 2008, doi: 10.1086/590560 [doi].

[16] J. F. Menton, S. M. Cremin, L. Canier, M. Horgan, and L. J. Fanning, "Molecular epidemiology of sexually transmitted human papillomavirus in a self referred group of women in Ireland," *Virol.J.,* vol. 6, p. 112, 2009 2009, doi: 1743-422X-6-112 [pii];10.1186/1743-422X-6-112 [doi].

[17] J. Sakamoto *et al.*, "Single type infection of human papillomavirus as a cause for high-grade cervical intraepithelial neoplasia and invasive cancer in Japan," *Papillomavirus Research,* vol. 6, pp. 46-51, 2018.

[18] N. C. Institute. "HPV and Cancer." National Cancer Institute. <https://www.cancer.gov/about-cancer/causes-prevention/risk/infectious-agents/hpv-and-cancer> (accessed January 13, 2020).

[19] I. M. de Kok *et al.*, "Primary screening for human papillomavirus compared with cytology screening for cervical cancer in European settings: cost effectiveness analysis based on a Dutch microsimulation model," *BMJ,* vol. 344, p. e670, 2012 2012. [Online]. Available: <http://www.ncbi.nlm.nih.gov/pubmed/22391612>.

[20] "Pap and HPV Testing," National cancer institute, 9/1/2014 2014, vol. 2015. [Online]. Available: <http://www.cancer.gov/types/cervical/pap-hpv-testing-fact-sheet>

[21] "Overview of the National Life Basic Survey." <https://ganjoho.jp/reg_stat/statistics/dl_screening/index.html#a16> (accessed.

[22] European Medicine Agency - Committee for Medicinal Products for Human Use, "Gardasil human papillomavirus vaccine [types 6, 11, 16, 18] (recombinant, adsorbed) - Summary of opinion," European Medicine Agency, Committee for Medicinal Products for Human Use, 4/25/2014. [Online]. Available: <http://www.ema.europa.eu/ema/index.jsp?curl=pages/medicines/human/medicines/000703/smops/Positive/human_smop_000672.jsp&mid=WC0b01ac058001d127>

[23] "Gardasil, Summary of Product Characteristics," European Medicines Agency, 2015 2015. [Online]. Available: <http://www.ema.europa.eu/ema/index.jsp?curl=pages/medicines/human/medicines/000703/human_med_000805.jsp>

[24] "Cervarix, Summary of Product Characteristics," European Medicines Agency, 2015 2015. [Online]. Available: <http://www.ema.europa.eu/ema/index.jsp?curl=pages/medicines/human/medicines/000721/human_med_000694.jsp&mid=WC0b01ac058001d124>

[25] B. Romanowski, "Long term protection against cervical infection with the human papillomavirus: review of currently available vaccines," *Hum.Vaccin.,* vol. 7, no. 2, pp. 161-169, 2/2011 2011, doi: 13690 [pii].

[26] "Merck's Investigational 9-valent HPV Vaccine, V503, Prevented 97 Percent of Cervical, Vaginal and Vulvar Pre-cancers Caused by Five Additional HPV Types, in Phase III Study." <http://www.mercknewsroom.com/news-release/research-and-development-news/mercks-investigational-9-valent-hpv-vaccine-v503-prevente> (accessed 12/1/2014, 2014).

[27] N. Li, S. Franceschi, R. Howell-Jones, P. J. Snijders, and G. M. Clifford, "Human papillomavirus type distribution in 30,848 invasive cervical cancers worldwide: Variation by geographical region, histological type and year of publication," *Int.J.Cancer,* vol. 128, no. 4, pp. 927-935, 2/15/2011 2011, doi: 10.1002/ijc.25396 [doi].

[28] J. Cuzick, "Gardasil 9 joins the fight against cervix cancer," *Expert review of vaccines,* pp. 1-3, May 31 2015, doi: 10.1586/14760584.2015.1051470.

[29] W. K. Huh *et al.*, "Final efficacy, immunogenicity, and safety analyses of a nine-valent human papillomavirus vaccine in women aged 16–26 years: a randomised, double-blind trial," *The Lancet,* vol. 390, no. 10108, pp. 2143-2159, 2017.

[30] E. A. Joura *et al.*, "A 9-valent HPV vaccine against infection and intraepithelial neoplasia in women," (in eng), *The New England journal of medicine,* vol. 372, no. 8, pp. 711-23, Feb 19 2015, doi: 10.1056/NEJMoa1405044.

[31] A. R. Giuliano *et al.*, "Nine-valent HPV vaccine efficacy against related diseases and definitive therapy: comparison with historic placebo population," *Gynecologic oncology,* vol. 154, no. 1, pp. 110-117, 2019.

[32] K. Yamabe, P. K. Singhal, M. Abe, E. J. Dasbach, and E. H. Elbasha, "The cost-effectiveness analysis of a quadrivalent human papillomavirus vaccine (6/11/16/18) for females in Japan," *Value in health regional issues,* vol. 2, no. 1, pp. 92-97, 2013.

[33] N. Yamamoto *et al.*, "Introducing HPV vaccine and scaling up screening procedures to prevent deaths from cervical cancer in Japan: a cost‐effectiveness analysis," *BJOG: An International Journal of Obstetrics & Gynaecology,* vol. 119, no. 2, pp. 177-186, 2012.

[34] E. H. Elbasha, E. J. Dasbach, and R. P. Insinga, "Supplementary Online Appendix: A Technical Report Accompanying Manuscript: Model for assessing human papillomavirus vaccination strategies," *Emerg.Infect.Dis.,* vol. 13, no. 1, pp. 28-41, 1/2007 2007, doi: 10.3201/eid1301.060438.

[35] "Japanese Ministry of Health, Labor and Welfare. Demographic data." <https://www.mhlw.go.jp/english/> (accessed July 13, 2020).

[36] J. A. f. S. Education, "8th Survey Report on Youth Sexual Behavior: Junior High School Students, High School Students, and University Students in Japan," 2018.

[37] "The Japan Cancer Society, Survey Report on Cervical Cancer Screening " <http://www.cczeropro.jp/kenshin/img/result/result.pdf> (accessed June 22, 2020.

[38] "MTI Ltd., Think Pearl, Joint Survey “About Cervical Cancer”," 2016. [Online]. Available: <https://prtimes.jp/main/html/rd/p/000000289.000002943.html>.

[39] E. H. Elbasha and E. J. Dasbach, "Impact of vaccinating boys and men against HPV in the United States," *Vaccine,* vol. 28, no. 42, pp. 6858-6867, 10/4/2010 2010, doi: S0264-410X(10)01175-8 [pii];10.1016/j.vaccine.2010.08.030 [doi].

[40] "Statistics of Medical Care Activities in Public Health Insurance." <https://www.e-stat.go.jp/stat-search/files?page=1&layout=datalist&toukei=00450048&tstat=000001029602&cycle=7&tclass1=000001130155&tclass2=000001130439&tclass3=000001130440> (accessed July 8, 2020).

[41] A. Ioka, H. Tsukuma, W. Ajiki, and A. Oshima, "Influence of age on cervical cancer survival in Japan," *Japanese journal of clinical oncology,* vol. 35, no. 8, pp. 464-469, 2005.

[42] Istituto Nazionale Tumori, "Eurocare Survival of cancer patient in Europe," Istituto Nazionale Tumori, 2015. [Online]. Available: <http://www.eurocare.it/Database/tabid/77/Default.aspx>

[43] K. A. Ault, "Effect of prophylactic human papillomavirus L1 virus-like-particle vaccine on risk of cervical intraepithelial neoplasia grade 2, grade 3, and adenocarcinoma in situ: a combined analysis of four randomised clinical trials," (in eng), *Lancet (London, England),* vol. 369, no. 9576, pp. 1861-8, Jun 2 2007, doi: 10.1016/s0140-6736(07)60852-6.

[44] S. M. Garland *et al.*, "Quadrivalent vaccine against human papillomavirus to prevent anogenital diseases," (in eng), *The New England journal of medicine,* vol. 356, no. 19, pp. 1928-43, May 10 2007, doi: 10.1056/NEJMoa061760.

[45] A. R. Giuliano *et al.*, "Efficacy of quadrivalent HPV vaccine against HPV Infection and disease in males," (in eng), *The New England journal of medicine,* vol. 364, no. 5, pp. 401-11, Feb 3 2011, doi: 10.1056/NEJMoa0909537.

[46] E. A. Joura *et al.*, "Efficacy of a quadrivalent prophylactic human papillomavirus (types 6, 11, 16, and 18) L1 virus-like-particle vaccine against high-grade vulval and vaginal lesions: a combined analysis of three randomised clinical trials," (in eng), *Lancet (London, England),* vol. 369, no. 9574, pp. 1693-702, May 19 2007, doi: 10.1016/s0140-6736(07)60777-6.

[47] J. M. Palefsky *et al.*, "HPV vaccine against anal HPV infection and anal intraepithelial neoplasia," (in eng), *The New England journal of medicine,* vol. 365, no. 17, pp. 1576-85, Oct 27 2011, doi: 10.1056/NEJMoa1010971.

[48] L. E. Markowitz, M. Drolet, N. Perez, M. Jit, and M. Brisson, "Human papillomavirus vaccine effectiveness by number of doses: Systematic review of data from national immunization programs," *Vaccine,* vol. 36, no. 32 Pt A, pp. 4806-4815, Aug 6 2018, doi: 10.1016/j.vaccine.2018.01.057.

[49] S. M. Garland *et al.*, "Quadrivalent vaccine against human papillomavirus to prevent anogenital diseases," *New England Journal of Medicine,* vol. 356, no. 19, pp. 1928-1943, 2007, doi: 10.1056/NEJMoa061760.

[50] M. Sawada *et al.*, "HPV vaccination in Japan: results of a 3-year follow-up survey of obstetricians and gynecologists regarding their opinions toward the vaccine," *International journal of clinical oncology,* vol. 23, no. 1, pp. 121-125, 2018.

[51] "Japanese Ministry of Health Labor and Welfare. National Health Insurance fee schedule," Tokyo, Japan, 2020.

[52] P. W. Sullivan, J. F. Slejko, M. J. Sculpher, and V. Ghushchyan, "Catalogue of EQ-5D scores for the United Kingdom," *Med.Decis.Making,* vol. 31, no. 6, pp. 800-804, 11/2011 2011, doi: 0272989X11401031 [pii];10.1177/0272989X11401031 [doi].

[53] G. Dominiak-Felden, C. Cohet, S. Atrux-Tallau, H. Gilet, A. Tristram, and A. Fiander, "Impact of human papillomavirus-related genital diseases on quality of life and psychosocial wellbeing: results of an observational, health-related quality of life study in the UK," *BMC.Public Health,* vol. 13, p. 1065, 2013 2013, doi: 1471-2458-13-1065 [pii];10.1186/1471-2458-13-1065 [doi].

[54] L. Alemany *et al.*, "Human papillomavirus DNA prevalence and type distribution in anal carcinomas worldwide," *International journal of cancer,* vol. 136, no. 1, pp. 98-107, 2015.

[55] X. Castellsagué *et al.*, "HPV involvement in head and neck cancers: comprehensive assessment of biomarkers in 3680 patients," *Journal of the National Cancer Institute,* vol. 108, no. 6, p. djv403, 2016.

[56] L. Alemany *et al.*, "Role of human papillomavirus in penile carcinomas worldwide," *European urology,* vol. 69, no. 5, pp. 953-961, 2016.

[57] L. Alemany *et al.*, "Large contribution of human papillomavirus in vaginal neoplastic lesions: a worldwide study in 597 samples," *European journal of cancer,* vol. 50, no. 16, pp. 2846-2854, 2014.

[58] S. De Sanjosé *et al.*, "Worldwide human papillomavirus genotype attribution in over 2000 cases of intraepithelial and invasive lesions of the vulva," *European Journal of Cancer,* vol. 49, no. 16, pp. 3450-3461, 2013.

[59] F. Aubin *et al.*, "Human papillomavirus genotype distribution in external acuminata condylomata: a Large French National Study (EDiTH IV)," *Clinical Infectious Diseases,* vol. 47, no. 5, pp. 610-615, 2008.

[60] H. W. Chesson, D. U. Ekwueme, M. Saraiya, M. Watson, D. R. Lowy, and L. E. Markowitz, "Estimates of the annual direct medical costs of the prevention and treatment of disease associated with human papillomavirus in the United States," *Vaccine,* vol. 30, no. 42, pp. 6016-6019, 2012/09/14/ 2012, doi: <https://doi.org/10.1016/j.vaccine.2012.07.056>.

[61] P. Boyle and D. M. Parkin, "Cancer registration: principles and methods. Statistical methods for registries," *IARC Sci Publ,* no. 95, pp. 126-58, 1991. [Online]. Available: <https://www.ncbi.nlm.nih.gov/pubmed/1894318>.

[62] I. Andrianakis *et al.*, "Bayesian history matching of complex infectious disease models using emulation: a tutorial and a case study on HIV in Uganda," *PLoS Comput Biol,* vol. 11, no. 1, p. e1003968, 2015.

[63] J. McCall, "Genetic algorithms for modelling and optimisation," *Journal of computational and Applied Mathematics,* vol. 184, no. 1, pp. 205-222, 2005.

[64] L. S. Bastos and A. O’Hagan, "Diagnostics for Gaussian process emulators," *Technometrics,* vol. 51, no. 4, pp. 425-438, 2009.
